# Data‐Driven Fatigue Prediction of Superalloys: A Novel Strategy Integrating Transfer Learning and Partial Label Learning for Addressing Ambiguous Data

**DOI:** 10.1002/advs.202507362

**Published:** 2025-11-07

**Authors:** Haopeng Lv, Jiawei Yin, Dayong Wu, Ziyuan Rao, Chao Su, Jie Kang, Qian Wang, Haikun Ma, Huicong Dong, Yandong Wang, Ru Su

**Affiliations:** ^1^ School of Materials Science and Engineering Hebei University of Science and Technology Shijiazhuang Hebei 050018 P. R. China; ^2^ National Engineering Research Center of Light Alloy Net Forming Shanghai Jiao Tong University Shanghai 200240 P. R. China; ^3^ State Key Laboratory for Advanced Metals and Materials University of Science and Technology Beijing Beijing 100083 P. R. China; ^4^ Taiji Computer Co., Ltd. Beijing 100012 P. R. China

**Keywords:** ambiguous data, fatigue performance, machine learning, partial label learning, superalloy

## Abstract

Machine learning has emerged as a powerful tool for predicting material properties due to its efficiency and accuracy. However, challenges related to data integrity, particularly the presence of ambiguous data, have limited its broad application. In this work, a novel strategy is proposed that integrates partial label learning and transfer learning to accurately address ambiguous compositional data in predicting the fatigue performance of superalloys. Subsequently, key microstructural features are enriched through thermodynamic calculations based on the composition data, enhancing model interpretability by revealing composition‐microstructure‐property relationships. This approach not only achieves superior predictive accuracy but also exhibits robust generalization across experimental validation. Given the widespread presence of ambiguous data, this framework holds significant potential for broader applications in materials science.

## Graphical abstract

A novel machine learning strategy tackles ambiguous compositional data to predict superalloy fatigue. By integrating partial label with transfer learning and enriching features through thermodynamic calculations, this approach achieves superior accuracy and interpretability. This robust, generalizable framework pioneers a new path for materials discovery, effectively addressing the prevalent challenge of data ambiguity in advanced materials research.

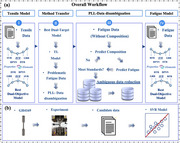

## 1 Introduction

Ni‐based superalloys are indispensable in various industrial applications due to their unique high‐temperature properties.^[^ ^1^, ^2^ ^]^ Fatigue‐induced fractures represent a primary failure mode during service and serve as key indicators for evaluating the quality and reliability of these materials. Therefore, accurate prediction of fatigue behavior is essential for the scientific design and reliable service of superalloys.^[^ ^3^ ^]^ However, accurately predicting fatigue performance across diverse alloy compositions remains challenging due to the complex composition‐processing‐microstructure‐property relationships. Recently, data‐driven machine learning (ML)^[^ ^4^, ^5^, ^6^ ^]^ methods have become powerful tools for predicting fatigue performance owing to their advantages in time efficiency and predictive accuracy.^[^ ^7^, ^8^, ^9^, ^10^, ^11^ ^]^ However, in practical applications, the absence and uncertainty of certain key features, such as composition and microstructure, from data sources such as previous publications often result in ambiguous data. This compromises data integrity, which in turn hinders the ability of the ML model to effectively learn the relationships between features and prediction targets. Consequently, this significantly reduces the accuracy, explainability, and generalization of the model.^[^ ^12^ ^,^ ^13^ ^]^

Confronted with ambiguous data, researchers have explored strategies such as incorporating physical constraints and advancing optimization algorithms to enhance the performance of models on small experimental datasets.^[^ ^14^, ^15^, ^16^, ^17^, ^18^, ^19^ ^]^ Despite these efforts, the generalization ability of current ML models is still circumscribed due to the scarcity of key features and an inadequate volume of data. To mitigate this, techniques like high‐throughput experiments and data imputation have also been introduced to enrich the dataset.^[^ ^20^, ^21^, ^22^ ^]^ However, the high costs associated with alloy manufacturing and fatigue testing make high‐throughput experiments time‐consuming and expensive, limiting their effectiveness in supplementing data.^[^ ^23^ ^]^ Additionally, due to the complexity of fatigue damage mechanisms in superalloys, data imputation through statistical interpolation cannot accurately replicate the nuances of actual data. As a result, these approaches have yet to fundamentally resolve the issues posed by ambiguous data.

Recent advancements in partial label learning (PLL), a new weakly supervised ML framework, offer innovative solutions for dealing with ambiguous data.^[^ ^24^ ^]^ PLL aims to resolve ambiguity by discerning correct instances from a pool of candidates. However, existing research has mostly focused on classification tasks, such as multimedia content analysis,^[^ ^25^ ^]^ human‐computer interaction^[^ ^26^ ^]^ and natural language processing,^[^ ^27^ ^]^ with limited exploration in the domain of regression problems. This limitation is particularly notable in materials science, where ambiguous data, such as processing temperatures, test parameters, and alloy compositions, often appear in the form of intervals or are missed.^[^ ^28^ ^]^ Traditional ML methods find it challenging to accurately extract the true data from these nearly infinite possibilities. Therefore, exploring the application of PLL in materials science, particularly in handling continuous ambiguous data, is crucial for resolving ambiguity and integrity issues, thereby enhancing the generalization ability of ML models.

**Figure 1:**
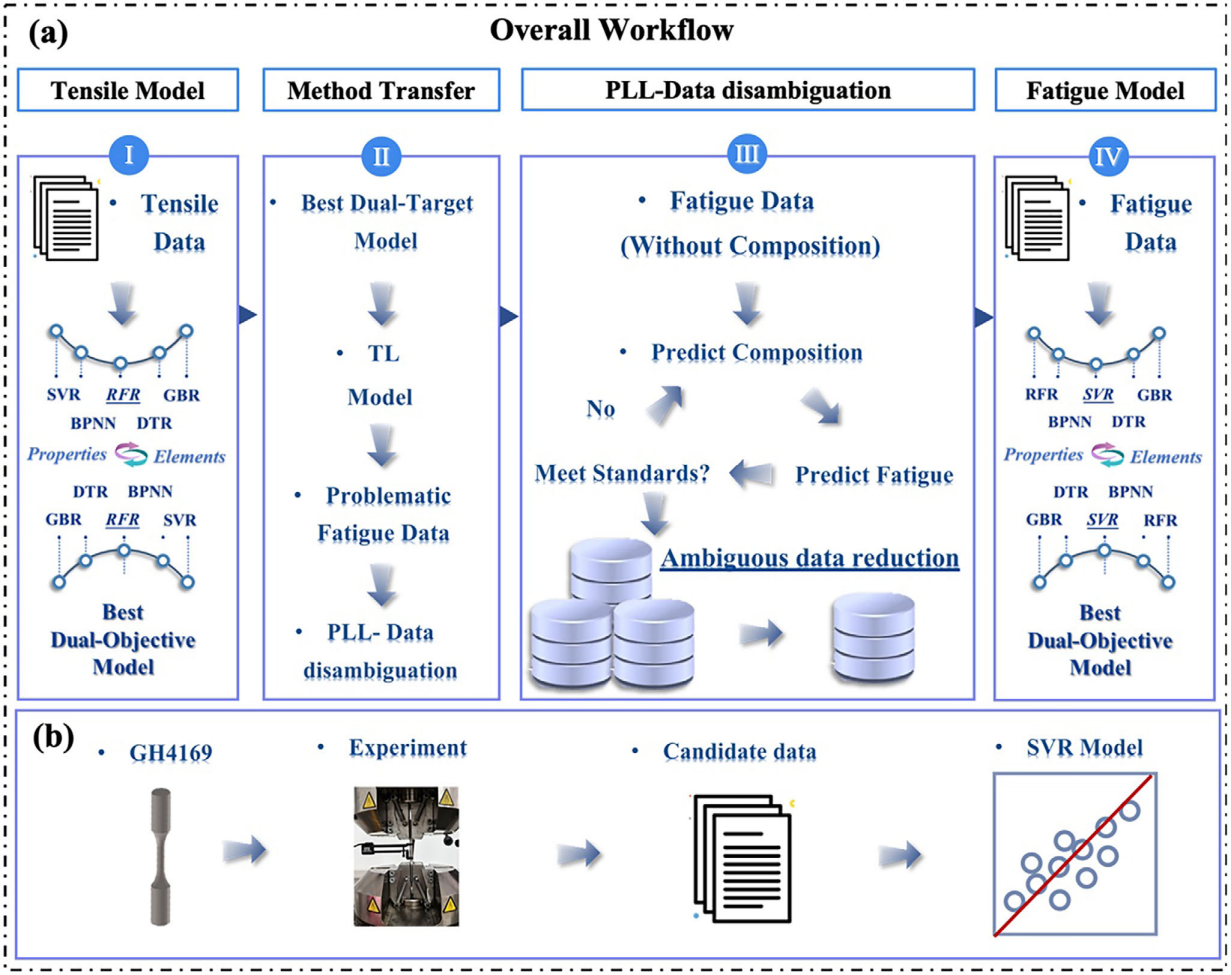
Overview of Methodology. a) A workflow framework for a novel approach combining PLL with TL for alloy composition mining and fatigue performance prediction. The framework is divided into four main steps: (I) Tensile properties and composition prediction; (II) TL method; (III) PLL method; (IV) Fatigue performance prediction. b) Experimental validation of the accuracy of the model in predicting fatigue performance. The specific steps include material preparation, fatigue testing, data collection, and model validation.

In this study, a new approach combining transfer learning (TL) and PLL is proposed to address the challenge of ambiguous data, particularly in the context of superalloy design, where alloy composition data are uncertain, as detailed in the workflow diagram shown in **Figure** **1**a. In cases where the labeling of fatigue stress and fatigue life data is incomplete, TL is used to facilitate knowledge sharing between tensile and fatigue performance, enabling the prediction of alloy compositions. Considering the complexity of actual alloy compositions, where each alloy composition instance has subtle differences, a PLL strategy combined with threshold‐setting methods was adopted. This approach ensures that the predicted alloy compositions are more reliable during the ambiguity resolution process. Subsequently, thermodynamic calculations are performed based on the accurately identified compositions for each alloy. These calculations supplement key physical properties in the dataset, such as the volume fraction of the precipitate phase and the solvus temperature of the precipitate phase, thereby enhancing the interpretability of the fatigue prediction model. Finally, the predicted fatigue performance is validated using 11 different alloys, with the relative errors of the predictions all falling within an acceptable range. Additionally, the SHapley Additive exPlanation (SHAP) method is employed to analyze the impact of chemical composition, heat treatment processes, and test parameters on fatigue performance. Given the ubiquity of ambiguous data in data collection, influenced by factors such as the confidentiality of research work, shallow research depth, and variations in research content, the generalized framework presented in this study is expected to facilitate the application of ML in other areas of materials science. Overall, the TL+PLL strategy introduced here consists of the following four key steps:

1. Basic process of tensile property prediction: forward performance prediction models (F‐P) and backward composition prediction models (B‐P) are formulated using multi‐objective support vector machine regression (SVR), random forest regression (RFR), decision tree regression (DTR), gradient boosted regression (GBR), and backpropagation neural network regression (BPNN) algorithms. Among these, the SVR and GBR algorithms do not support multi‐objective regression, so they must be wrapped with the MultiOutputRegressor to enable multi‐objective predictions.
2. TL Method: to address the challenge posed by ambiguous fatigue data and the difficulty of data collection, we first employed the structural transfer learning (STRUT) method described in Ref.[29] This approach transfers optimal RFR algorithms from the F‐P and B‐P models originally trained on the source domain (Tensile Database), to the target domain (Fatigue Database). The transferred models are fine‐tuned using unambiguous fatigue data collected from the literature. Since the source and target domains have different input features, the STRUT method incorporates a feature adaptation mechanism, which locally adjusts decision tree thresholds in the RFR algorithm to better align with the data distribution of the target domain.^[^ ^29^ ^]^ The effectiveness of the STRUT method and the relationship between the source and target domains are further analyzed and visualized in Section S2 and Figures S3–S9 (Supporting Information).
3. PLL method: In order to further eliminate ambiguous data and extract usable data, a cyclic optimization approach is employed to extract chemical compositions suitable for fatigue life modeling. This method ensures a one‐to‐one correspondence between composition and properties by continuously extracting alloy compositions. Each optimization cycle undergoes rigorous validation. If the difference between the predicted fatigue stress value and the actual value is below a predetermined threshold (*e_1_*), the chemical composition is considered satisfactory. Conversely, if the threshold is not reached, the prediction process proceeds to subsequent cycles until the new alloy composition meets the stringent requirements (see Figure 4a for a detailed description of the process).
4. Basic process of fatigue performance prediction: the application of the F‐P model allows accurate prediction of the tensile properties of fatigue compositions under specified test conditions, which are then carefully compiled into the fatigue database. Subsequently, V*_p_*, T*_p_*, E, G, and SFE are introduced into the model. Detailed microstructural information is provided in Section S8 and Table S17 (Supporting Information). Multi‐objective regression predictions are made using SVR, RFR, GBR, DTR, and BPNN algorithms, which help to determine the most appropriate regression model for fatigue stress and fatigue life.

In order to further verify the generalizability of the model, the GH4169 alloy was prepared. Figure 1b shows the experimental validation of the fatigue performance model. After collecting the experimental data of the GH4169 alloy samples, predictions were made using the optimal regression model.

## 2 Results

### 2.1 Tensile Properties Prediction and Composition Prediction

**Figure 2:**
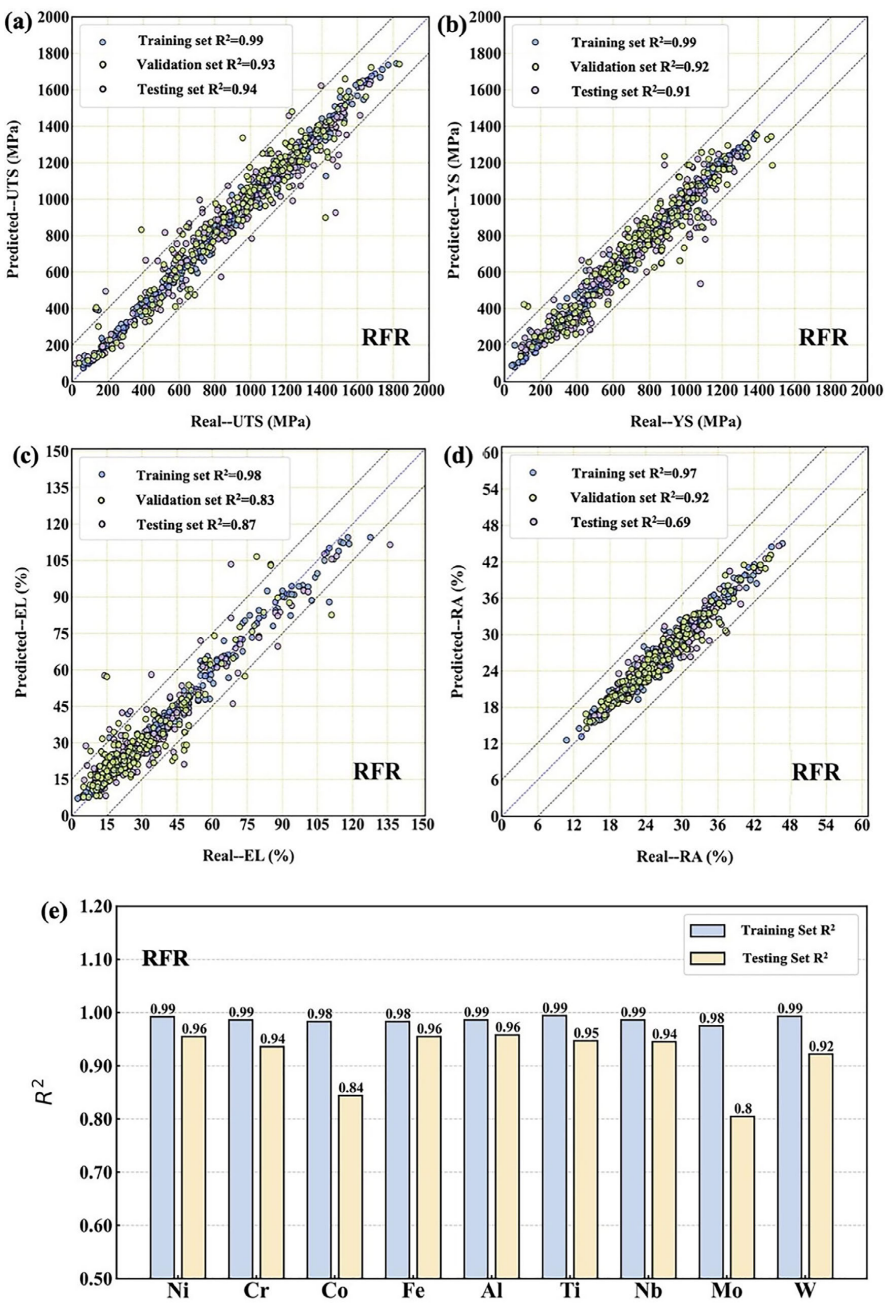
Model predictions overview for training, validation, and testing datasets: a) F‐P model predictions for UTS. b) YS. c) EL. d) RA. e) B‐P model predictions for Ni, Cr, Co, Fe, Al, Ti, Nb, Mo, and W in training and test datasets.

This study employs optimal algorithms to train the F‐P and B‐P models. Inputs to the F‐P include **Ni**, Cr, Co, Fe, Al, Ti, Nb, Mo, W, T, ST, STt, STat, Stat, AT, and At, with outputs comprising UTS, YS, EL, and RA. Inputs to the B‐P include UTS, YS, EL, RA, T, ST, STt, STat, Stat, AT, and At. Outputs comprise Ni, Cr, Co, Fe, Al, Ti, Nb, Mo, and W. Furthermore, the performance of each model is evaluated using the R^2^ and RMSE metrics across the training, validation, and testing sets to determine the optimal model choice. **Figure** **2**a–d present scatter plots comparing the predicted and actual values for UTS, YS, EL, and RA, with R^2^ values exceeding 0.80, demonstrating good predictive performance. Figure 2e presents the R^2^ values for the RFR algorithm for both the training and testing sets. In the B‐P model, the R^2^ values for all elements in the training set are above 0.9, while in the testing set, most elements maintain R^2^ values above 0.9, with only Mo and Co being slightly lower. The detailed results can be found in Section S1 (Supporting Information), which includes Figure S1 and Table S1 (Supporting Information). The relatively poor predictions for Co and Mo are thoroughly analyzed from both the data quality perspective and the functional roles of the elements in Ni‐based superalloys. A detailed discussion appears in Section S1 (Supporting Information), with supporting evidence illustrated in Figure S2 (Supporting Information). Nonetheless, the predictive performance with R^2^ > 0.8 still satisfies the requirements for expanding the dataset.^[^ ^30^, ^31^ ^]^ Given the superior accuracy of the RFR algorithm, it was selected to train the F‐P and B‐P models in this study.

### 2.2 Structural Transfer

**Figure 3:**
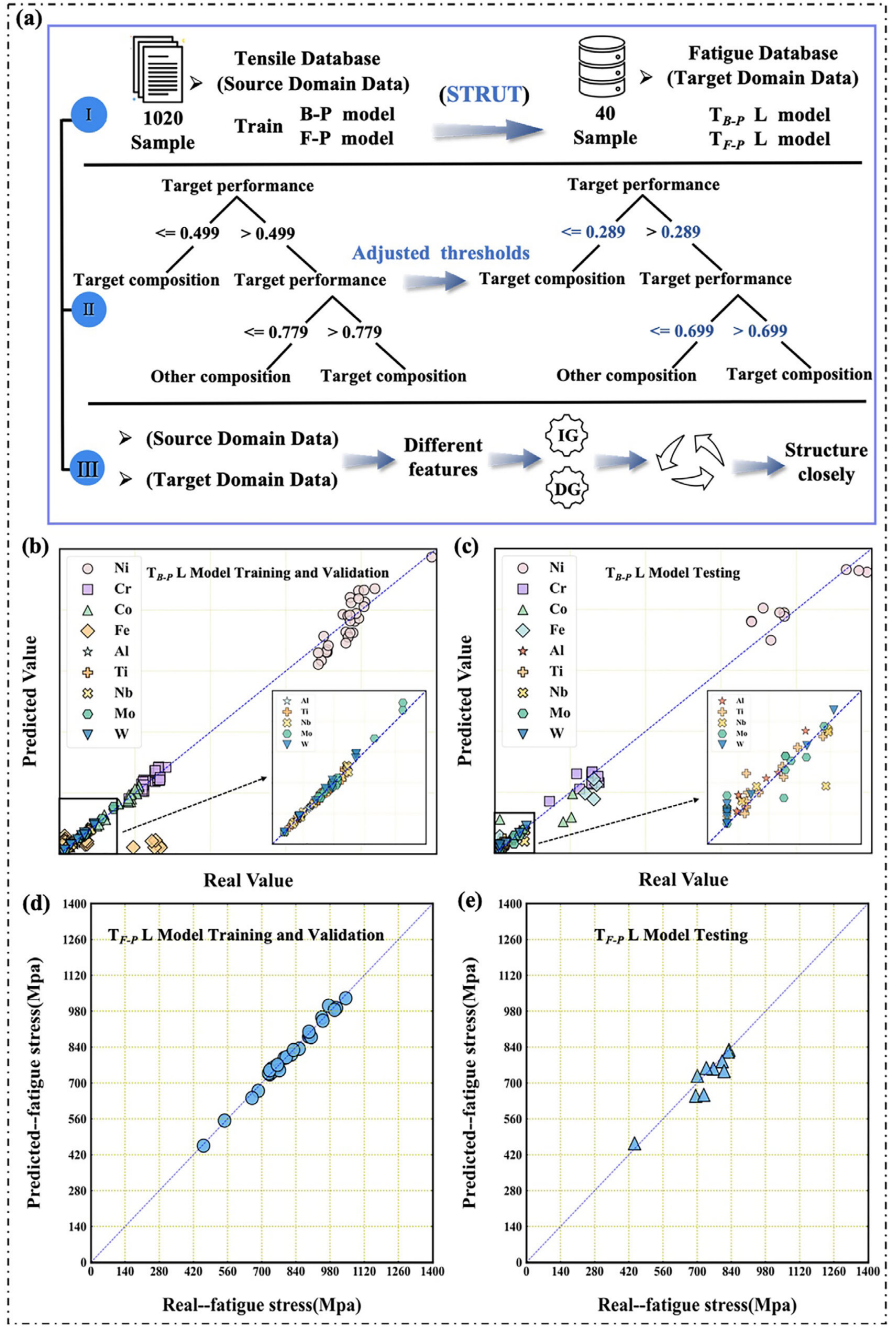
Comprehensive Flowchart of TL: a) Specific details of STRUT: (I) Detailed flow of STRUT; (II) A simplified example illustrating how STRUT optimizes threshold values in a decision tree model; (III) A diagram illustrating the process of structural STRUT for handling feature inconsistencies. b)‐e) Schematic representations of the training and validation results of the T*_B‐P_* L and T*_F‐P_* L models.

**Figure** **3**b presents a scatter plot comparing the predicted and actual values of the T*_B‐P_* L model across 30 sample sets, demonstrating that the model effectively fits the data and stabilizes the parameters. Detailed composition information is provided in Table S2 (Supporting Information). Figure 3c demonstrates that, after fixing the alloy composition, the model maintains good predictive performance on a new dataset. The MAPE values for Ni, Cr, Al, Ti, Nb, and W are close to 10%, demonstrating high prediction accuracy for these elements (for detailed alloy information, please refer to Figures S10 and S11 and Table S3, Supporting Information). The prediction accuracy for Fe, Co, and Mo is relatively lower, likely due to the lower initial R^2^ values and the fact that, in some alloy grades, the element values are zero, which the model failed to capture. Figure 3d,e presents the prediction results of the T*_F‐P_* L model on the training and validation sets. The scatter points are closely clustered around the diagonal, demonstrating good data fitting, and structural transfer significantly enhances the generalization ability and prediction accuracy of the model.

### 2.3 Data‐Aware Disambiguation Methods

The PLL framework proposed in this study aims to optimize noisy labels, improve data quality, and enhance the generalization ability of the model. It consists of four steps: alloy composition prediction, quality validation, composition optimization, and composition supervision. First, the T*_B‐P_* L model is used to predict alloy compositions, and quality validation is performed to filter reliable data. Subsequently, the maximum margin criterion and maximum likelihood criterion are applied to optimize the alloy compositions,^[^ ^32^, ^33^, ^34^, ^35^ ^]^ eliminating data uncertainties and ensuring the accuracy of the training data. In the alloy composition prediction phase, the T*_B‐P_* L model generates preliminary estimates. However, because the model is retrained only on a small fatigue dataset, the predictions can contain substantial errors, leading to lower accuracy in fatigue performance prediction (see Figure S12c,d, Supporting Information). Therefore, quality validation must be performed before proceeding to the composition optimization step to identify reliable data and provide a foundation for subsequent corrections. At this stage, the predicted alloy compositions are input into the T*_F‐P_* L model, and the maximum margin criterion is used to optimize the labels. By setting an error threshold (*e_1_* = 3%), the model can effectively distinguish between true labels and noisy labels, thereby improving generalization performance (detailed criteria are provided in Section S3 and Figure S13, Supporting Information). The prediction results are then divided into true labels (where the error is below the threshold) and noisy labels (where the error exceeds the threshold).

After completing quality validation, the process moves to the composition optimization stage. The goal is to gradually align the distribution of noisy‐labeled data with that of the true‐labeled data, thereby further refining the predicted compositions. The maximum margin criterion enlarges the decision boundary between true‐labeled data and noisy‐labeled data, enabling the model to more accurately distinguish between them. Meanwhile, the maximum likelihood criterion optimizes the conditional probability distribution of labels, ensuring their convergence toward the true labels and effectively reducing data noise. To achieve composition optimization, a Gaussian distribution model is introduced to quantify data uncertainty.^[^ ^36^, ^37^ ^]^ Specifically, the mean vector (μ) and covariance matrix ($\left(\Sigma \right)$) of the reliable data are calculated, and based on these, a perturbation vector Δ is constructed to adjust the distribution of the unreliable data. This perturbation vector is computed as follows^[^ ^38^ ^–^ ^41^ ^]^:

(1) $$\Delta =step\_size*\sum^{-1}\;\left(x-u\right)$$

where $\sum^{-1}$ is the inverse of the covariance matrix, *x* − μ represents the difference vector, and *step_size* is the key parameter controlling correction precision, ensuring that unreliable data gradually converges toward the true data distribution. Detailed *step_size* parameter settings are provided in Section S4 (Supporting Information) and illustrated in Figure S14 (Supporting Information). Additionally, to ensure that the adjustment direction consistently points toward the true data distribution, the Mahalanobis distance is introduced as a measurement criterion. As shown in Equation (2):

(2) $$D_{m}\left(x\right)=\sqrt{{\left(x-\mu \right)}^{T}\sum^{-1}\left(x-\mu \right)}$$

Here, *x* denotes the noisy label point to be computed, μ is the mean vector of the sample, $\sum^{-1}$ is the covariance matrix of the data, which describes the correlation between features, $\sum^{-1}$ is the inverse of the covariance matrix, T represents the transpose operation, and *x* − μ denotes the difference vector. The Mahalanobis distance fully considers the covariance between features, allowing it to effectively measure the relative position of multidimensional data points.^[^ ^38^, ^41^ ^]^

**Figure 4:**
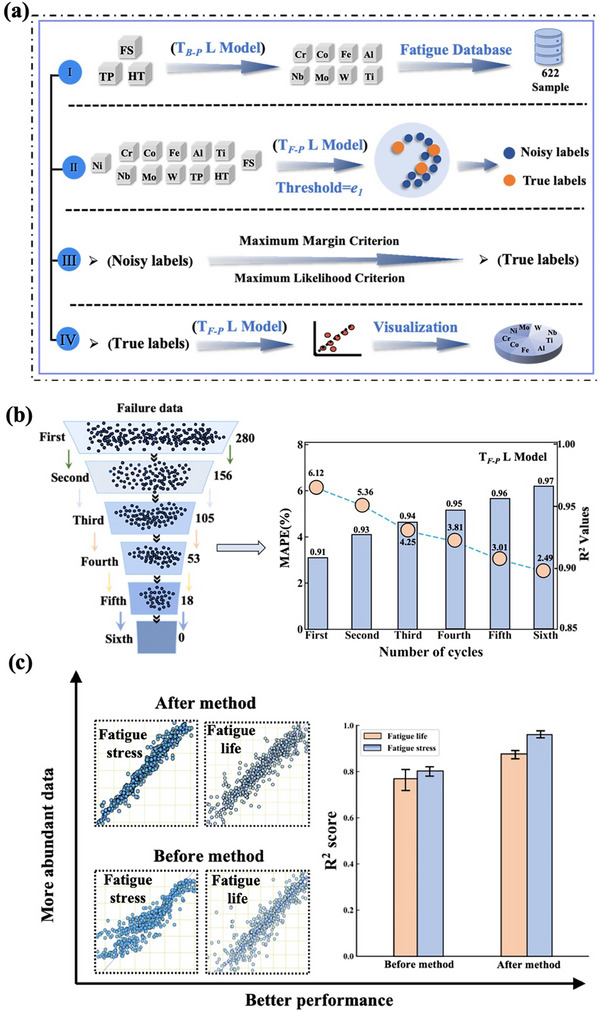
Flowchart of the PLL: a) Schematic of the data disambiguation process and the evolution of failed data after each optimization cycle, TP (T, Δε*_t_*, Δε*_e_*, Δε*_p_*), HT (ST, STt, Stat, AT, At), FS (Fatigue stress); b) Evolution of the MAPE and R^2^ values of the T*_F‐P_* L model across optimization cycles; c) Comparison of the MAPE and R^2^ values of the SVR model before and after the application of the PLL strategy.

The optimized noisy labels were transformed into new data samples and reintroduced into the T*_F‐P_* L model for validation and threshold determination. During this process, some noisy labels were successfully converted into true labels. As the optimization cycle progressed, more true labels were used for training, thereby improving the accuracy and stability of the model. **Figure** **4**b shows that as the number of validation cycles increased, the number of noisy labels decreased significantly, while the number of true labels gradually increased. Initially, there were 280 sets of noisy labels; after optimization, data ambiguity was reduced. By the sixth validation cycle, the number of noisy labels dropped to zero, indicating that the PLL framework significantly enhanced reliability. Figure 4b shows the changes in the MAPE and R^2^ values of the T*_F‐P_* L model during the optimization process, with MAPE decreasing from 6.12% to 2.49% and R^2^ improving from 0.91 to 0.97, demonstrating that the optimization significantly improved data quality and prediction accuracy. As shown in Figure 4c, after PLL disambiguation, the R^2^ value for fatigue stress increased from 0.91 to 0.97, and MAPE decreased from 6.12% to 2.89%. For fatigue life, the R^2^ value improved from 0.76 to 0.89, and MAPE decreased from 14.70% to 7.50%, further demonstrating the reliability of the PLL strategy. Detailed evaluation results for the other algorithmic models are provided in Figure S12a,b (Supporting Information).

Compared to stress data, fatigue life data exhibit higher uncertainty and scatter, which primarily arise from the following three aspects: I) microstructural differences, even under the same macroscopic composition and process conditions, microstructural variations persist in the alloys (grain sizes, phase distributions, and internal defects). These microstructural variations exert significantly greater influence on fatigue life than on stress properties.^[^ ^42^, ^43^ ^]^ II) Experimental variability: small changes in load control, environmental conditions (temperature), and specimen preparation in fatigue experiments may result in significant scatter in fatigue life data.^[^ ^42^ ^]^ III) Stochastic crack behavior: fatigue life is influenced by the stochastic nature of crack initiation and propagation processes, and this inherent randomness further exacerbates data scatter.^[^ ^44^ ^]^ To quantify the variability in fatigue life data, a statistical analysis was performed on the fatigue database. The results indicated that, under identical composition and test conditions, the standard deviation of fatigue life data was significantly higher than that of stress data. To quantify this, a statistical analysis was performed on the fatigue database. For samples with identical composition and testing conditions, the coefficient of variation for fatigue life was found to be ≈10.7, which is significantly higher than that for stress data (≈3.2). Detailed information is provided in Section S4 and Figure S15 (Supporting Information). This increased variability directly led to lower model accuracy in fatigue life prediction compared to stress prediction, as demonstrated by the higher MAPE values for fatigue life prediction.

To thoroughly evaluate the performance advantages of the TL+PLL method, systematic comparative experiments were conducted with three benchmark methods: the standalone transfer learning method, the standalone partially labeled learning method, and the standalone traditional linear interpolation method. The detailed experimental process is described in Section S4 and Figure S16 (Supporting Information). Meanwhile, to further highlight the advantages of the TL+PLL approach, we also compared it against simpler uncertainty quantification (UQ) baselines. The detailed experimental procedure is provided in Section S4 and Figure S17 (Supporting Information). Given that this study employs multiple models with multi‐feature inputs, many features exhibit obvious correlations. To rigorously validate the impact of eliminating feature correlations on the accuracy of the TL+PLL model, we designed and implemented a novel multi‐level experimental framework in the Supporting Information. This framework includes various feature‐selection and dimensionality‐reduction strategies to ensure the statistical robustness and generalizability of the TL+PLL method in subsequent modeling. Detailed information and processes are provided in Section S5 and Figures S18–S20 (Supporting Information). Finally, this study further discusses the model computational complexity and the sensitivity to hyperparameter choices in the PLL optimization process. The detailed experimental procedure is provided in Section S5 and Figures S21 and S22 (Supporting Information).

## 3 Discussion

### 3.1 Experimental Validation of the Model

To improve data quality and address potential issues, experimental validation of the F‐P model and the T*_F‐P_* L model was conducted before starting the cycle optimization. Alloy GH4169 was used for this validation, and detailed experimental procedures and conditions are provided in Section S6 and Tables S4 and S5 (Supporting Information). Compared to experimental values, the F‐P model predicted tensile strength with only a 2% error (Figure S23, Supporting Information), whereas the T*_F‐P_* L model achieved a 4% error for fatigue stress. The high accuracy of the latter is key to effectively supervising the optimization process; by providing reliable labels during training, it ensures that the alloy composition is precisely aligned with the target fatigue performance, thereby enhancing the reliability and accuracy of the optimization results.

To validate the generalization ability of the established ML model, 11 sets of Ni‐based superalloys used in engineering applications were selected for testing. Of these, 5 datasets are from published papers and 6 datasets are from actual fatigue tests, with the samples obtained under different test conditions, not included in the original training dataset of the ML model. Among the 5 alloys collected, alloy grades adapted to different engineering conditions were included, such as AD730, GH4720Li, GH4742, GH451, and GH4065. Actual fatigue experiments were performed using the GH4169 room‐temperature fatigue test and the GH4169g high‐temperature fatigue test, with details provided in Tables S6–S9 (Supporting Information). The 11 groups of superalloys were predicted using the trained SVR algorithmic model for UTS, YS, EL, and RA, respectively, under the corresponding experimental conditions. The comparison of the predicted values with the experimental values is shown in Figure S23c–f (Supporting Information). The specific relative errors are presented in **Table** **1**. It can be observed that the relative errors of UTS and YS are low, while those for EL and RA are higher. However, all errors fall within the accepted error range of 10%. This further demonstrates the ability of the developed tensile model to generalize to new data. Figure S23g,h (Supporting Information) shows the validation plots of the fatigue performance model. It can be seen from the plots that the relative errors in the predicted fatigue stress and fatigue life are within permissible limits. This indicates that the SVR algorithm model has a strong generalization ability and is able to accurately predict the fatigue properties of alloys outside the range of the dataset.

**Table 1:** MAPE values for combined gold for 11 alloys.

Alloy Properties	UTS	YS	EL	RA	FS	FL
MAPE	2.82%	4.97%	6.9%	7.63%	4.48%	8.06%

To further validate the generalization capability and engineering applicability of the TL+PLL model, additional validation tests were conducted under two challenging scenarios: extreme operating conditions (ultra‐high and ultra‐low strain amplitudes) and novel alloy systems (high‐entropy alloys). Validation was performed using samples independent of the original database, including 9 sets of ultra‐low strain samples, 11 sets of ultra‐high strain samples, and 1 set of high‐entropy alloy samples tested under different conditions. Detailed alloy composition information and experimental parameters are provided in Tables S10–S12 (Supporting Information), with the prediction results shown in Figure S24 (Supporting Information). Under ultra‐high strain amplitude conditions, the prediction error of the model increased slightly but remained within the acceptable range for engineering applications (error <10%). Under ultra‐low strain amplitude conditions, the model demonstrated excellent stability and accuracy. Although high‐entropy alloys differ significantly from conventional Ni‐based superalloys in compositional complexity and microstructural characteristics, the fatigue prediction model still produced promising predictions. Given that the model was never trained on this class of materials, the relative prediction error of 12% is remarkably low and strongly demonstrates the generalization potential of the model.

To further assess the applicability and generalization capability of the model in a broader context, data on single‐crystal and directionally solidified Ni‐based superalloys were systematically collected from published literature and patents for comprehensive validation. Detailed information is provided in Tables S13 and S14 (Supporting Information). The multi‐objective SVR model was applied to the single‐crystal and directionally solidified alloy datasets to evaluate prediction accuracy, with the results shown in Figure S25 (Supporting Information). The validation results indicate that the fatigue life prediction model achieves an R^2^ of 0.82 for the single‐crystal alloy dataset and 0.81 for the directionally solidified alloy dataset. Although these values are slightly lower than those obtained from the polycrystalline alloy dataset, they still demonstrate good predictive performance. The slight reduction in predictive accuracy is primarily attributed to the anisotropic properties of single‐crystal alloys, which require a more comprehensive consideration of the synergistic effects between crystal orientation and microstructural features.

Overall, the fatigue performance prediction model performs well when applied to single‐crystal and directionally solidified alloys, establishing a solid foundation for engineering applications. Moreover, the validation results under extreme strain conditions and novel alloy systems further demonstrate its strong generalization capability. Nevertheless, to better accommodate the unique microstructural features of specialized alloys and the complexity of real‐world service conditions, further optimization of the model remains necessary for future work.

### 3.2 Model Interpretability Analysis

**Figure 5:**
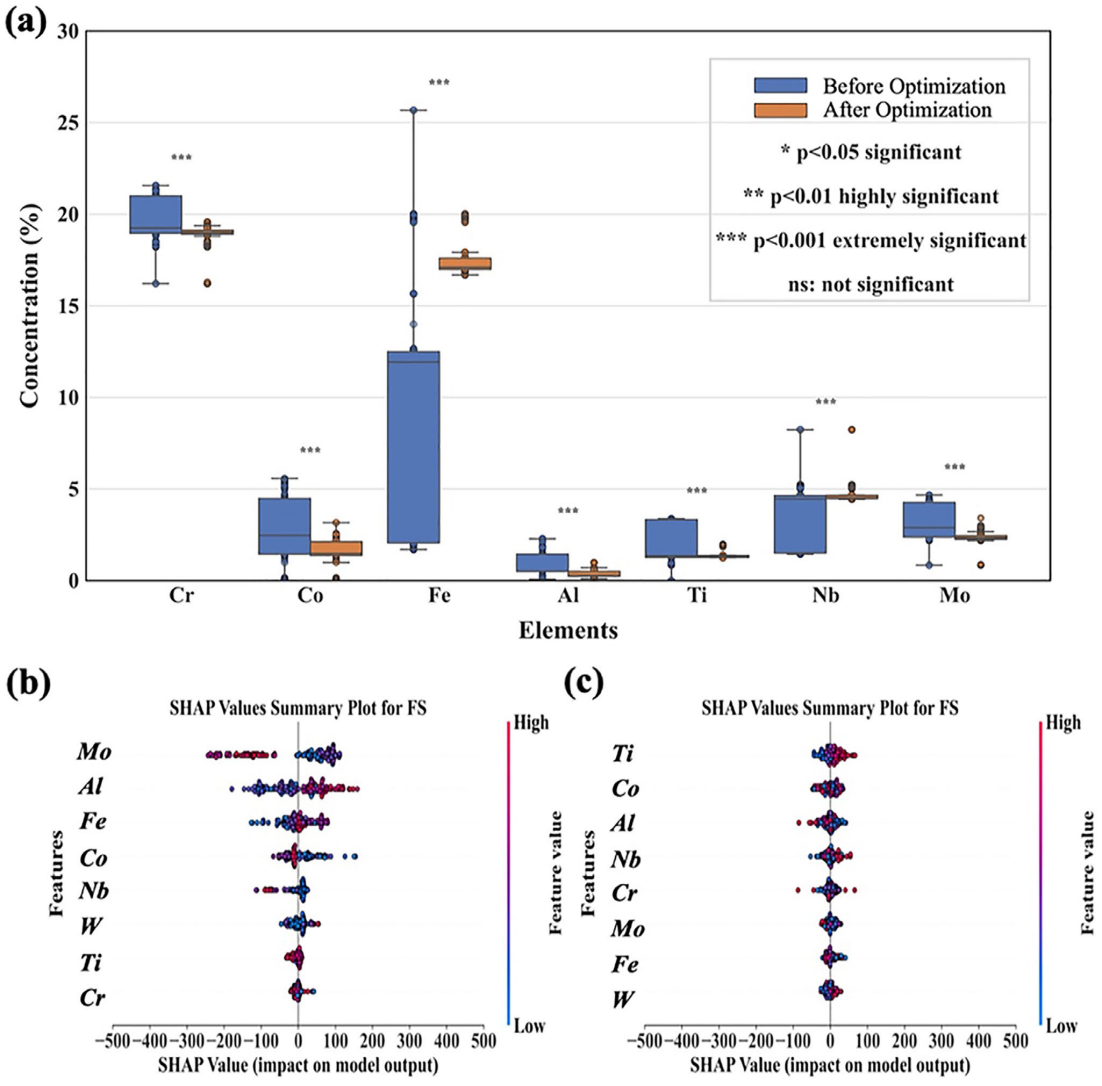
Visualizes model result analysis: a) Results of GH4169 alloy data analysis, showing changes in its main compositions before and after optimization using box and line plots; b) Distribution of GH4169 alloy data before cycle optimization; c) Distribution of GH4169 alloy data after cycle optimization.

**Figure** **5**a presents box plots showing the distribution of major alloy compositions before cyclic optimization, including the median and interquartile range. To enhance the interpretability and accuracy of the data visualization, we applied a standardized significance labeling system based on Welch's test results: ^***^ indicates *p*<0.001 (extremely significant), ^**^ indicates *p*<0.01 (highly significant), ^*^ indicates *p*<0.05 (significant), and ns indicates no significant difference. These significance markers were added above each box plot to visually represent the statistical differences in elemental concentrations before and after disambiguation. A comparison reveals that the composition distribution of GH4169 alloy changed after optimization, with reduced dispersion and a more concentrated median, indicating improved data stability and consistency. This confirms the effectiveness of the optimization process in reducing compositional fluctuations and enhancing prediction accuracy.

Given the impracticality of visualizing PLL disambiguation across all 30 alloy grades, we selected GH4169 as a representative case due to its widespread use and stable fatigue performance. Our cyclic optimization process uses a maximum likelihood criterion to adjust the compositional distribution of noisy‐labeled data to match the distribution of true labels, thereby refining compositions to meet target performance objectives. SHAP analysis confirms the efficacy of this strategy. Post‐optimization, the SHAP value distributions for key strengthening elements (Ti, Al, Nb, Co) became significantly more concentrated, indicating reduced data discretization. This reduction in compositional fluctuation led to a more stable and accurate model, as evidenced by a significant increase in the R^2^ value and a decrease in prediction error (Figure S26, Supporting Information).

To evaluate the applicability of the empirical model proposed by Pang et al.^[^ ^45^, ^46^ ^]^:

(3) $$\sigma_{f}=\left(C-P\cdot UTS\right)\;\cdot UTS$$

where *C* and *P* are material‐related fitting parameters and σ_*f*_ denotes the fatigue stress. We conducted a systematic study on the relationship between the UTS and FS of materials based on the tensile and fatigue database of Ni‐based superalloys. Statistical analysis (see Figure S27, Supporting Information for details) reveals that this relationship exhibits significant temperature dependence. In the medium‐to‐low temperature range (T < 600 °C), UTS and FS show a strong linear correlation (determination coefficient R^2^ = 0.94, see Figure S27b, Supporting Information), confirming the effectiveness of Equation (3) in this interval. However, when the temperature rises above 600 °C, the linear correlation between the two significantly weakens (R^2^ = 0.62, see Figure S27c, Supporting Information). This deviation in performance at high temperatures is mainly attributed to complex microstructural evolution (such as dynamic changes in γ' phases, dislocation slip, and grain boundary slip), which introduces strong nonlinearity into the fatigue behavior of the material, exceeding the linear characterization capability of the original empirical model.

Given the limitations of Equation (3) in the high‐temperature region and the lack of a universal analytical model describing this relationship in the literature,^[^ ^47^ ^]^ this study introduces data‐driven machine learning methods to modify and extend the traditional empirical formula. This approach aims to construct a new model that can accurately capture and embed the complex nonlinear effects at high temperatures, thereby improving the prediction accuracy across a broader temperature range. We extended the linear relationship in Equation (3) to a nonlinear form by introducing a quadratic term:

(4) $$\sigma_{f}=\left(C-P\cdot UTS+Q\cdot UTS^{2}\right)\;\cdot UTS$$

Considering the temperature sensitivity of material properties at high temperatures, a temperature correction factor T was introduced and an exponential decay function was used to simulate the temperature effect:

(5) $$\sigma_{f}=\left(C-P\cdot UTS+Q\cdot UTS^{2}\right)\;\cdot UTS\cdot exp\left(-\alpha \cdot T\right)$$

An SVR algorithm was employed to optimize the parameters (*C, P, Q*, and α) in the modified model (Equation 5), utilizing the high‐temperature UTS and FS data from the database. The validation results (Figure S27d,e, Supporting Information) demonstrate that this revised model yields a significant improvement in prediction accuracy under high‐temperature conditions, with the coefficient of R^2^ increasing from 0.62 for the original linear model to 0.89. Moreover, the revised model maintains strong predictive performance at room and moderate temperatures, ensuring its generalizability and robustness across the entire temperature range.

**Figure 6:**
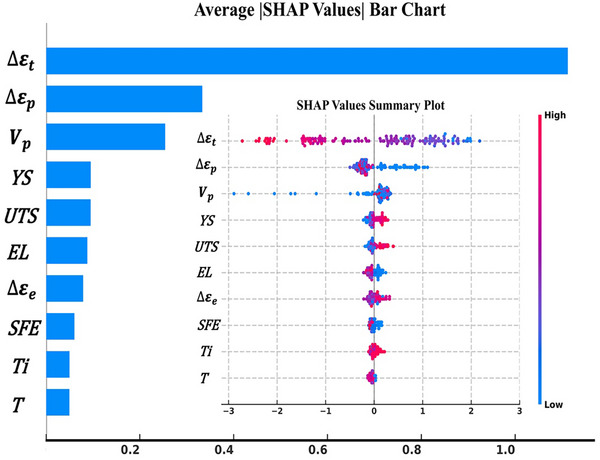
Distribution of SHAP values across 10 features.

This study employs the SHAP method to evaluate and rank the importance of key features, with the top ten presented in **Figure** **6**, enhancing the interpretability of the machine learning model to deeply analyze the interplay between material properties, microstructure, and fatigue behavior in Ni‐based superalloys, thereby guiding targeted alloy design to improve material reliability and longevity; however, SHAP only conducts multi‐feature impact analysis without altering the internal predictive logic of the model or directly examining its training process. To more intuitively illustrate the model training dynamics and elucidate its predictive mechanisms, we included “learning curve” analysis and partial dependence plot (PDP) analysis in Section S7 (Supporting Information) to further demonstrate the prediction results of the model. The detailed process and results are shown in Figures S28 and S29 (Supporting Information).

#### 3.2.1 Total and Plastic Strain

**Figure 7:**
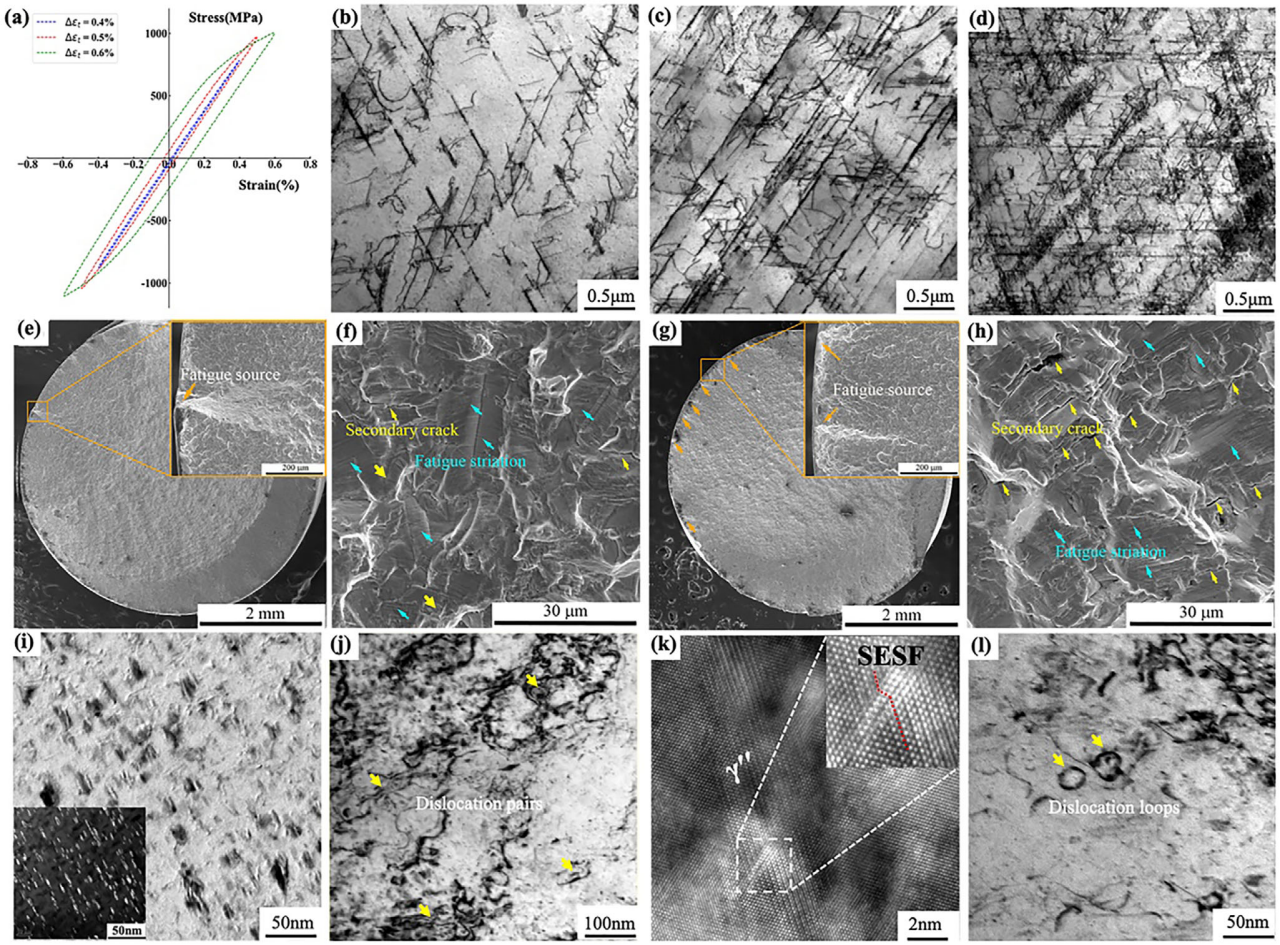
Hysteresis loop curves a) and the corresponding TEM images b–d) At 500 cycles for the GH4169 alloy under different total strain amplitudes: b) 0.4%, c) 0.5% and d) 0.6%; Macroscopic fracture morphology and secondary crack morphology of GH4169 alloy at 0.6% and 1.0% strain amplitude: e, f) 0.6%; g, h) 1.0%; TEM images showing the typical interaction mechanisms between dislocations and precipitates of the experimental samples in different cycles with a total strain amplitude of 0.6%: i) Bright and dark field TEM images showing the precipitates, B//[110]; j) Dislocation pairs left by APB shearing after 100 cycles; k) SSFs shearing after 700 cycles; l) Dislocation ring left by Orowan bypassing after 700 cycles.

Domain knowledge regarding microstructural evolution during cyclic loading, specifically strain‐induced dislocation development and precipitate‐dislocation interactions, formed the foundation of our modeling framework. SHAP analysis identified total and plastic strain as the principal drivers of fatigue damage. Under constant total strain, plastic strain accumulated progressively with cycle number, altering dislocation arrangements and densities and thereby shifting the dominant failure mechanism from single slip to multiple slip and eventually to stacking‐fault formation or twinning.^[^ ^48^, ^49^, ^50^, ^51^ ^]^ A transition from planar to wavy slip could not be excluded. Experimentally, increasing strain amplitude at a fixed cycle count accelerated the transition from single to multiple slip (**Figure** **7**a–d). By contrast, alloys displayed only minor differences in fatigue life under identical test conditions (Figure S30a,b, Supporting Information), suggesting that chemical composition exerted a limited direct influence. In the same alloy, however, fatigue life varied markedly with strain amplitude, underscoring the predominant effect of loading parameters. These findings aligned with the SHAP results and confirmed that test conditions governed fatigue life. Consequently, strain‐related variables were selected as the core input features.

Further research found that the model accurately links macroscopic loading conditions to the final damage modes. SHAP analysis explicitly identifies total strain amplitude (Δε*_t_*) and plastic strain amplitude (Δε*_p_*) as the key detrimental factors for fatigue life. This finding is in perfect agreement with direct physical evidence: a comparison of the fracture surfaces under different strain amplitudes (Figure 7e–h) shows that the high‐strain (1.0%) sample exhibits multiple fatigue initiation sites, wider fatigue striations, and numerous secondary cracks. These are direct physical manifestations of accelerated damage accumulation driven by higher plastic strain.^[^ ^52^, ^53^, ^54^ ^]^ Furthermore, this macroscopic outcome is governed by the cycle‐dependent evolution of microstructural deformation mechanisms, a process that the model has implicitly captured. Under constant total strain, the plastic strain evolves as the dominant deformation mechanism transitions.^[^ ^55^, ^56^ ^]^ In the early cycles, when plastic deformation is high, the deformation mode is primarily dominated by dislocation shearing of the precipitates, forming characteristic dislocation pairs (Figure 7i,j). This efficient shearing mechanism leads to the initial rapid cyclic hardening. As cyclic deformation progresses and damage accumulates, the mechanical mechanism undergoes a dynamic transition. Dislocation movement is impeded, and in later cycles, the deformation mechanism gradually transitions toward a mode dominated by Orowan bypassing, leaving behind dislocation loops (Figure 7l). While the actual deformation process involves multiple complex interactions (e.g., the stacking faults observed in Figure 7k), it is the interplay between these two primary competing mechanisms, shearing and bypassing, that fundamentally determines the evolution of the material's plastic deformation capacity, manifesting as sustained cyclic hardening and a further reduction in Δε*_p_*.

#### 3.2.2 Precipitates

**Figure 8:**
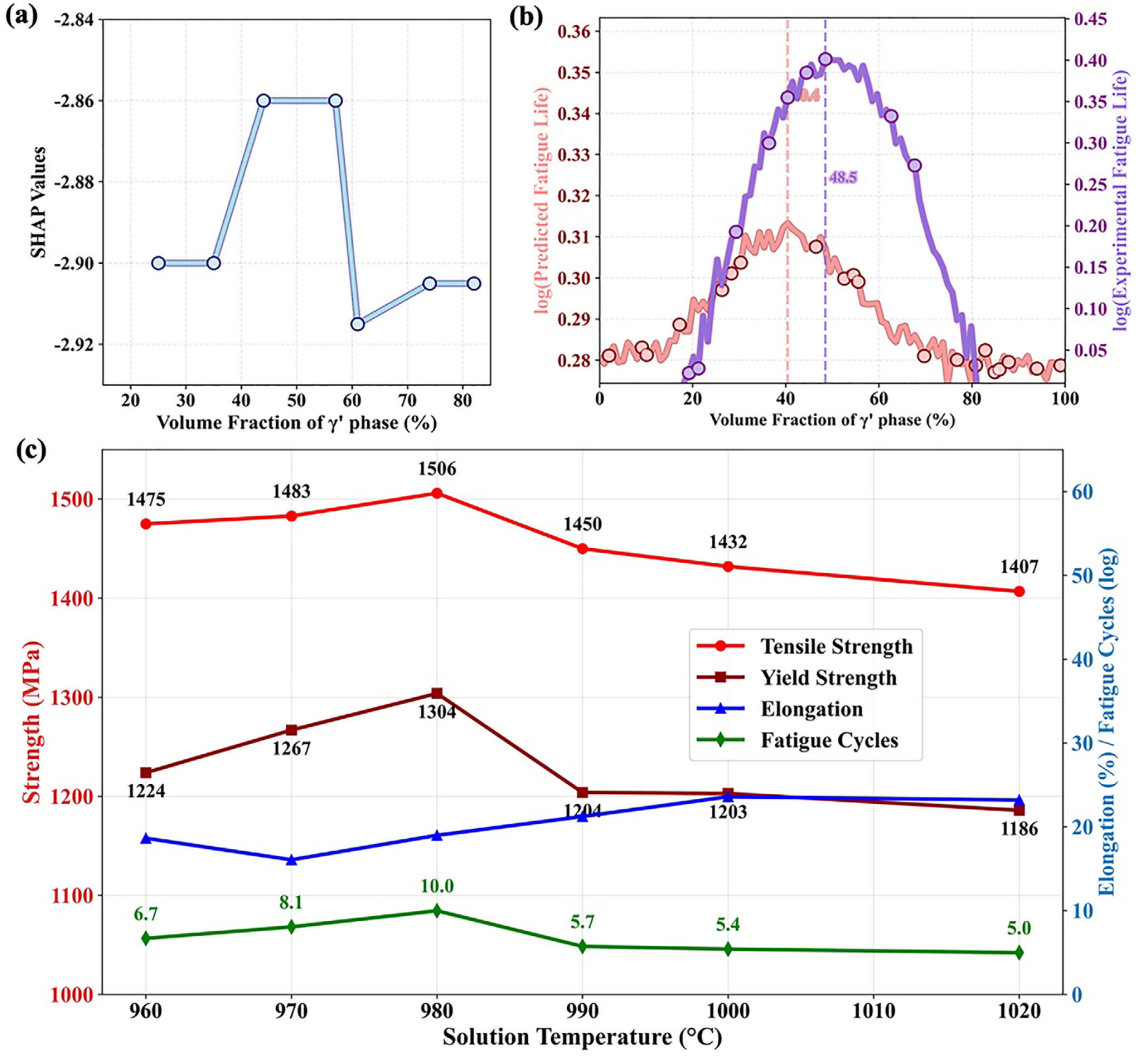
Model‐driven analysis and experimental validation of the non‐monotonic relationship between microstructure, processing, and fatigue life. The figure demonstrates the model's ability to capture complex physical metallurgy principles. a) shows SHAP values indicating the non‐monotonic impact of γ' volume fraction (V*_p_*) on the model's prediction, with an optimal range of 40–60%. This is validated in b), where both the model prediction (purple curve) and experimental data confirm a peak in fatigue life at an optimal V*_p_* of 48.5%. The metallurgical context is provided in c), which shows that this optimal microstructure and peak fatigue life are achieved at a specific solution treatment temperature (980 °C), representing a complex trade‐off with other mechanical properties like tensile and yield strength.

To quantify the effect of precipitates on fatigue performance, we integrated the predictive model with SHAP analysis for feature attribution (**Figure** **8**a,b). The analysis reveals a key insight consistent with extensive metallurgical research: a moderate volume fraction of the strengthening phase enhances fatigue life, whereas excessively high or low fractions lead to its deterioration.^[^ ^57^, ^58^, ^59^, ^60^ ^]^ Our model's successful capture of this non‐monotonic relationship, peaking at a γ' volume fraction of ≈40–50%, is not a statistical artifact but reflects a fundamental trade‐off in the underlying deformation and damage mechanisms.

This phenomenon is a well‐established principle in the design of Ni‐based superalloys. On one hand, a low volume fraction (V*_p_* < 40%) results in insufficient precipitation strengthening. The material possesses low resistance to cyclic plastic deformation, leading to rapid damage accumulation and premature fatigue crack initiation. Conversely, a very high volume fraction (V*_p_* > 50%) leads to an “over‐strengthened” state. In this regime, the dominant deformation mechanism transitions from efficient dislocation shearing to Orowan bypassing, a shift that promotes highly localized planar slip.^[^ ^61^, ^62^, ^63^ ^]^ This strain localization into intense persistent slip bands (PSBs) creates preferential sites for crack nucleation at the material surface or at precipitate‐matrix interfaces, thereby accelerating fatigue failure despite the high static strength.^[^ ^64^, ^65^ ^]^

Yield strength marks the onset of plastic deformation. Under cyclic loading, a higher yield strength in superalloys reduces plastic deformation and fatigue damage, leading to higher fatigue limits and extended life at lower stress levels. Similarly, higher tensile strength enables the material to withstand greater maximum stress (Figure 8c), improving fracture resistance during crack propagation and ultimately prolonging fatigue life.

## 4 Conclusion 

This study proposes an innovative TL combined with a PLL model that effectively addresses the challenge of poor predictive performance in materials ML due to data scarcity. The model was successfully applied to predict the fatigue performance of superalloys. The main conclusions are summarized as follows:

1. Through a novel method combining TL and PLL, we successfully extracted accurate alloy compositions from ambiguous datasets and enhanced data integrity by incorporating thermodynamically computed physical features closely related to fatigue performance. The prediction accuracy of fatigue performance based on the testing dataset exceeds 90%.
2. The model exhibits high sensitivity to conditions and compositions, making it suitable for evaluating fatigue performance across various service conditions and alloy grades. Predictions of fatigue performance for six experimental alloy samples showed an error of less than 10% compared to actual experimental values.
3. By updating the dataset and incorporating more specific physical information, the model has narrowed the gap between model interpretability and material insights. In the meantime, by combining experimental results with the SHAP method, the model can rationally explain the influence of added alloy compositions and physical features, as well as evaluate the impact of different features on fatigue performance.

Overall, this method leveraged the intrinsic relationships between material knowledge, significantly accelerating dataset expansion and improving ML prediction accuracy without relying on extensive manual data processing. Furthermore, the method demonstrates strong generalizability, making it easily extendable to other materials systems, such as aluminum alloys and high‐entropy alloys. These extended applications will be the focus of future work.

## 5 Model

In this study, the tensile data for Ni‐based deformation superalloys were collected and extracted from reliable public literature and patents, and the low‐cycle fatigue test data were extracted from the China Superalloys Handbook.^[^ ^28^ ^]^ The microstructural features related to fatigue performance were calculated in batches using Thermo‐Calc software. The detailed data information is provided in Section S8 and Tables S15–S17 (Supporting Information).

Significant numerical disparities exist among the input features as detailed in Tables S15–S17 (Supporting Information), affecting the prediction accuracy due to their varied distributions.^[^ ^66^ ^]^ To counter this, a normalization step was undertaken for all input features in the database, adjusting their value ranges from 0 to 1. This process aims to minimize bias resulting from differences in feature scales, thereby contributing to the improvement of the model accuracy. In addition, the fatigue life was logarithmically transformed to facilitate the prediction of fatigue life, as shown in Equation (6).

(6) $$Y^{*}=\log\left(Y\right)$$

To ensure the reliability and generalization of our machine learning model for predicting stretching performance, the dataset was randomly partitioned into training (55%), validation (25%), and test (20%) sets. This division strategy provides a robust framework for model development and evaluation, which is particularly important given the lack of a standardized protocol for dataset splitting.^[^ ^67^ ^]^ The model's generalization ability was further affirmed through a ten‐fold cross‐validation process during the training phase.

In this investigation, the accuracy of the ML model was evaluated by using R^2^, RMSE, and MAPE as represented by Equations (7)–(9), respectively.

(7) $$R^{2}=1\frac{\sum_{i=1}^{n}\left(y_{measured}-y_{predicted}\right)}{\sum_{i=1}^{n}\left(y_{measured}-y_{mean}\right)}$$

(8) $$RMSE=\sqrt{\frac{\sum_{i=1}^{S}\left(y_{predicted}-y_{measured}\right)}{n}}$$

(9) $$MAPE=\frac{100\%\sum_{i=1}^{n}\left|\frac{\left(y_{predicted}-y_{measueed}\right)}{y_{predicted}}\right|}{n}$$

where *y_measured_* denotes the actual output, *y_predicted_* denotes the predicted output, *y*_mean_ represents the average of the actual output, and *n* is the number of data points. R^2^ is one of the most important metrics for evaluating the accuracy of a regression model. MAPE and RMSE are other important metrics for evaluating the prediction error of models.

Interpretability has always been a desired property of ML models due to their “black box” nature. SHapley Additive exPlanation algorithm^[^ ^68^ ^]^ is used to improve the interpretability of ML models. This technique assesses the interpretability using Shapley values, where higher values indicate a greater influence of features on the model results.

## Conflict of Interest

The authors declare no conflict of interest.

## Supporting information



Supporting Information

## Data Availability

Data and original code used for training/testing the ML model, validation of the model, and formal analysis have been deposited at the GitHub repository: https://github.com/lvhaopeng/TL‐PLL.git. These are publicly available as of the date of publication. Further inquiries are welcome by the lead contact.
