# Supplementary material for: Data‐Driven Fatigue Prediction of Superalloys: A Novel Strategy Integrating Transfer Learning and Partial Label Learning for Addressing Ambiguous Data
Source: Adv Sci (Weinh). 2025 Nov 7;13(5):e07362. doi: 10.1002/advs.202507362 (PMC12850054; doi:10.1002/advs.202507362)
Supplement: Supplementary file 1 — Supporting Information [file ADVS-13-e07362-s001.docx]

**Data-driven fatigue prediction of superalloys: a novel strategy integrating transfer learning and partial label learning for addressing ambiguous data**

Haopeng Lv^a,1^, Jiawei Yin^a,1^, Dayong Wu^a,c*^, Ziyuan Rao^b^[[1]](#footnote-1)^*^, Chao Su^d^, Jie Kang^a^, Qian Wang^a^, Haikun Ma^a^, Huicong Dong^a^, Yandong Wang^c^, Ru Su^a*^

*^a^ School of Materials Science and Engineering, Hebei University of Science and Technology, Shijiazhuang, Hebei, 050018, China*

*^b^ National Engineering Research Center of Light Alloy Net Forming, Shanghai Jiao Tong University, Shanghai, PR China*

*^c^ State Key Laboratory for Advanced Metals and Materials, University of Science and Technology Beijing, Beijing 100083, China*

*^d^* *Taiji Computer Co., Ltd., Beijing, 100012, China*

**1. Details of tensile performance prediction**

We employed five regression models, including Support Vector Regression (SVR), Random Forest Regression (RFR), Gradient Boosting Regression (GBR), Back Propagation Neural Network (BPNN) and Decision Tree Regression (DTR), to predict tensile properties (UTS, YS, EL and RA) and compositions. Figure S1 respectively display the RMSE and R^2^ values for these five different prediction models.


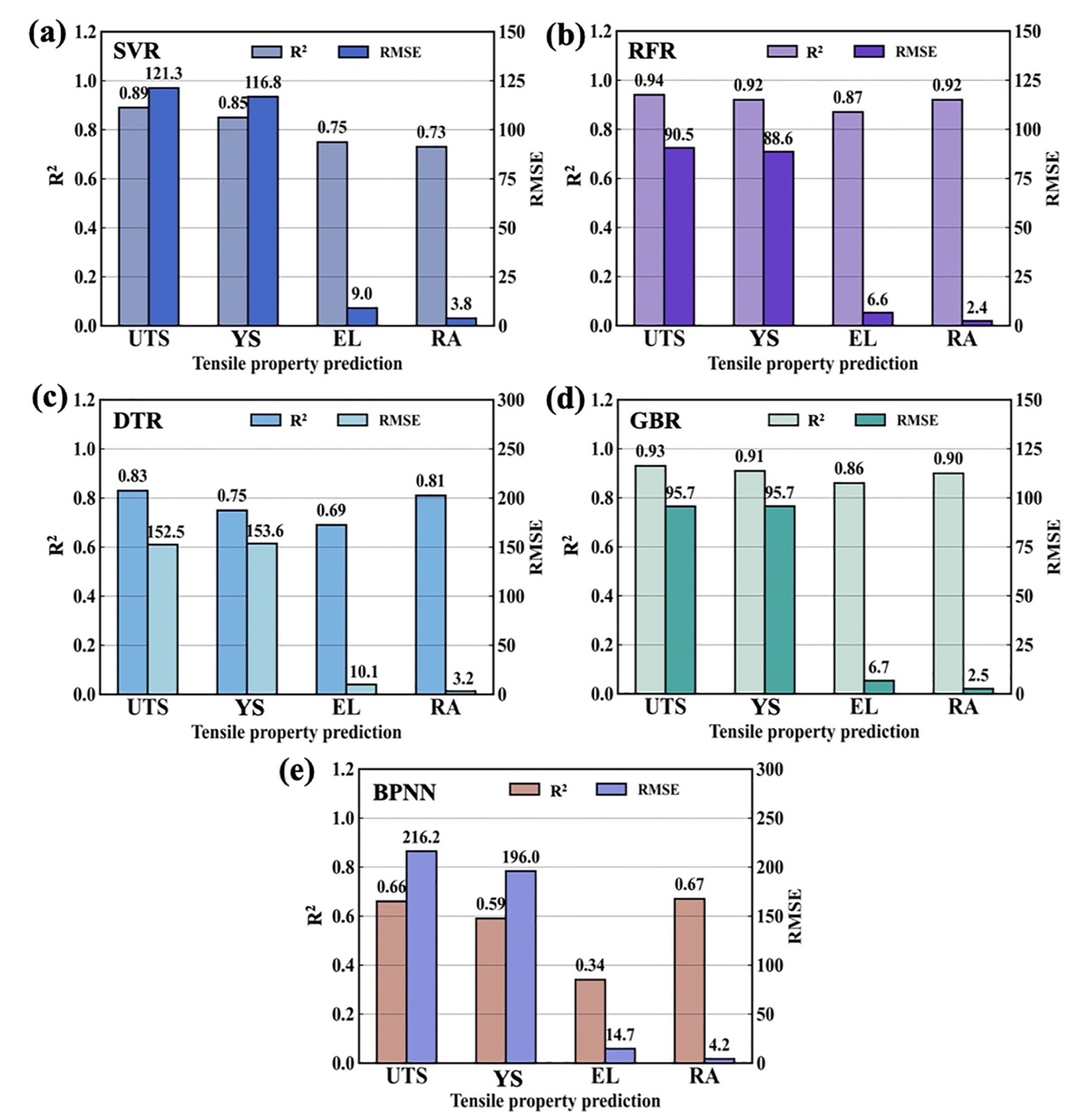


**Figure S1.** The overall R^2^ and RMSE indicators for tensile performance under various algorithmic models are as follows: a) SVR, b) RFR, c) DTR, d) GBR and e) BPNN.

Before selecting the optimal algorithm for predicting tensile performance, we evaluated several multi-objective regression algorithms, including SVR, DTR, GBR, and BPNN. Figures S1a-e display the R² and RMSE values for each model on the tensile performance prediction validation set. The results indicate that the RFR model outperforms the others, achieving the highest prediction accuracy with a R² value exceeding 0.90. Among the other performance metrics, RFR also demonstrates the best accuracy for predicting EL, although with a slightly lower R² value of approximately 0.87. Given that EL is generally more difficult to predict than UTS or YS, these results suggest that our EL predictions are more accurate than those of other algorithms.^[1,2]^ This may be due to EL's higher sensitivity to internal defects, indicating the need for a more comprehensive understanding of impurities, manufacturing processes and factors related to defect formation to further improve prediction accuracy.

Compared to single-objective regression models, multi-objective regression models can predict multiple targets simultaneously by sharing computational resources and network structures. This not only enhances training and testing efficiency but also saves computational time and resources. Additionally, when multiple target variables are correlated, such as UTS and EL, multi-objective regression better leverages the data by sharing features, thereby improving prediction accuracy.

**Table S1.** Evaluation of five models for 9 major alloying chemical elements.

| Algorithm | | SVR | RFR | DTR | GBR | BPNN |
| --- | --- | --- | --- | --- | --- | --- |
| Ni | Train-R^2^ | 0.72 | 0.99 | 1.00 | 0.95 | 0.95 |
|  | Test-R^2^ | 0.73 | 0.95 | 0.94 | 0.90 | 0.90 |
| Cr | Train-R^2^ | 0.69 | 0.99 | 1.00 | 0.96 | 0.96 |
|  | Test-R^2^ | 0.65 | 0.94 | 0.91 | 0.92 | 0.92 |
| Co | Train-R^2^ | 0.60 | 0.98 | 1.00 | 0.92 | 0.92 |
|  | Test-R^2^ | 0.52 | 0.84 | 0.76 | 0.77 | 0.77 |
| Fe | Train-R^2^ | 0.65 | 0.98 | 1.00 | 0.96 | 0.96 |
|  | Test-R^2^ | 0.74 | 0.96 | 0.92 | 0.95 | 0.95 |
| Al | Train-R^2^ | 0.80 | 0.99 | 1.00 | 0.95 | 0.95 |
|  | Test-R^2^ | 0.75 | 0.96 | 0.89 | 0.93 | 0.93 |
| Ti | Train-R^2^ | 0.89 | 0.99 | 1.00 | 0.97 | 0.97 |
|  | Test-R^2^ | 0.84 | 0.95 | 0.86 | 0.93 | 0.93 |
| Nb | Train-R^2^ | 0.68 | 0.99 | 1.00 | 0.94 | 0.94 |
|  | Test-R^2^ | 0.73 | 0.94 | 0.88 | 0.90 | 0.90 |
| Mo | Train-R^2^ | 0.56 | 0.97 | 1.00 | 0.91 | 0.91 |
|  | Test-R^2^ | 0.54 | 0.81 | 0.64 | 0.69 | 0.69 |
| W | Train-R^2^ | 0.78 | 0.99 | 1.00 | 0.97 | 0.97 |
|  | Test-R^2^ | 0.64 | 0.92 | 0.88 | 0.90 | 0.90 |


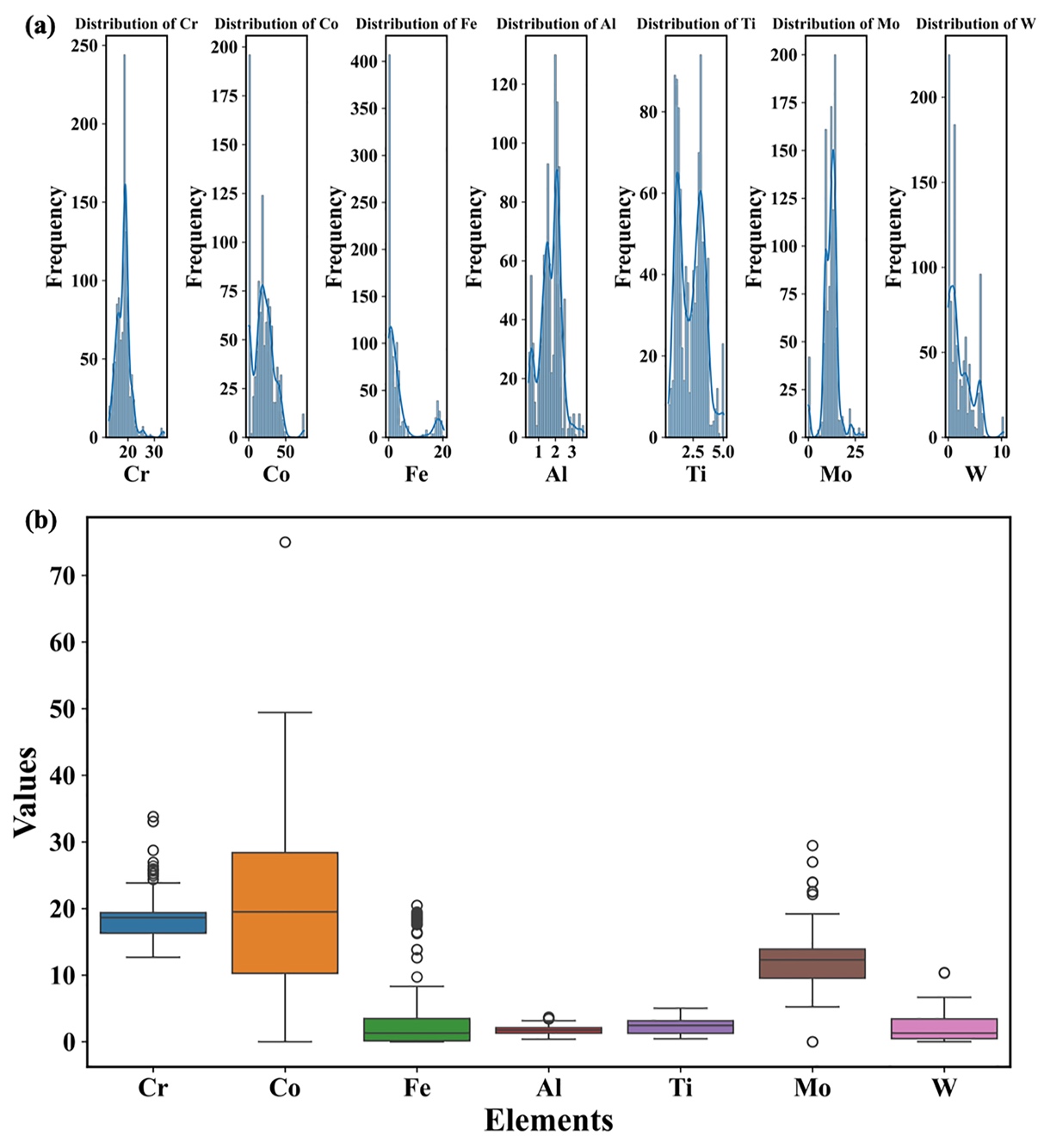


**Figure S2.** Statistical distribution analysis of major alloying elements in the database.

a) Frequency distribution histograms of elemental contents for Cr, Co, Fe, Al, Ti, Mo, and W; b) Box plots of each element showing the range, median, outliers and degree of dispersion in their concentrations. It is evident that Co and Mo exhibit high dispersion and multiple outliers, indicating greater difficulty in their prediction.

Further analysis of Mo and Co distributions in the database reveals that their high numerical dispersion and the presence of very low or zero values significantly affect prediction accuracy in the inverse tensile model. As shown in Figure S2, Mo and Co exhibit uneven concentration distributions, with some values at zero. Consequently, when predicting Mo and Co concentrations, the model tends to overfit to frequently occurring values while demonstrating a weaker predictive capability for outliers and sparse regions. This leads to larger errors when predicting samples in highly dispersed regions.

Mo and Co play important strengthening roles in Ni-based superalloys. Mo significantly enhances the strength and fatigue properties of alloys through solid solution strengthening and promotion of γ'/γ'' phase precipitation, while Co enhances the creep and fatigue resistance of alloys by stabilizing the γ'/γ'' phase and decreasing the stacking fault energy (SFE).^[3,4]^ Previous studies have demonstrated that small fluctuations in Mo content can lead to changes in the volume fraction and size of the γ'/γ'' phase, while changes in Co content affect the stability of the γ'/γ'' phase.^[5]^ Analysis of the composition prediction model revealed that the model input lacks microstructural features such as γ'/γ'' phase volume fraction and size, which makes it difficult to accurately capture the complex effects of small changes in Mo and Co content on microstructure and mechanical properties, resulting in prediction errors. Additionally, the roles of Co and Mo as solid solution strengthening elements are relatively weak compared to the γ'/γ'' phase strengthening mechanism.^[3,6]^ The database primarily emphasizes γ'/γ'' phase strengthening characteristics, exhibiting lower sensitivity to changes in Co and Mo content, which consequently results in poor prediction performance.

**2. Details of TL Model**

This study employs a structural transfer learning approach for B-P and F-P model migration, resulting in two migration prediction models: the T*_B-P_* L model and the T*_F-P_* L model. The B-P model is an inverse composition prediction model that uses RFR as the optimal algorithm and is trained on 1,020 stretch samples from the source domain. The input features of the model include UTS, YS, EL, RA, T, ST, STt, STat, Stat, AT and At, while its output features comprise Ni, Cr, Co, Fe, Al, Ti, Nb, Mo and W.

The target domain dataset is derived from published literature and experimental results, comprising 40 sets of samples in total. Of these, 30 sets are used for structural migration training and 10 sets are reserved for model validation. For the T*_B-P_* L model, the input features include FS, ST, STt, STat, Stat, AT, At, T, Δε*_t_*, Δε*_e_* and Δε*_p_*, while the output features remain Ni, Cr, Co, Fe, Al, Ti, Nb, Mo and W.

A comparison of the feature sets reveals seven common features that primarily describe the material's heat treatment process: T, ST, STt, STat, Stat, AT and At. These shared parameters provide a consistent foundational input space for the model. Each domain also possesses four unique features: the source domain is characterized by static tensile properties (UTS, YS, EL, RA), whereas the target domain is defined by fatigue properties (FS, Δε*_t_*, Δε*_e_*, Δε_p_). This feature structure is well-founded, as the final mechanical properties of a material are fundamentally determined by its heat treatment and composition. By training on the extensive source domain dataset, the B-P model has learned the complex relationships between the seven shared process parameters and the material's resulting mechanical behavior. This acquired knowledge, encapsulated in the model weights, represents the governing principles of the material " composition-process-performance" relationship. Consequently, this knowledge is readily transferable to the target domain, establishing a robust basis for predicting fatigue properties.

The UTS and YS of a material directly indicate its resistance to plastic deformation. In fatigue testing, where stress levels are relatively low, fatigue life is primarily governed by the crack initiation phase. Higher UTS and YS typically correlate with an increased fatigue limit or fatigue stress (FS), enabling the material to endure greater cyclic stresses without yielding or initiating cracks. This relationship is a cornerstone of fatigue research.^[7]^ Consequently, insights derived from UTS and YS measurements can be effectively applied to predict and understand the FS of a material.

EL and RA are macroscopic indicators of a material's plasticity or toughness. In low-cycle fatigue (LCF), materials experience significant plastic deformation with each cycle. The Coffin-Manson relationship establishes a power-law correlation between Δε*_p_* and fatigue life (FL).^[8]^ Materials exhibiting greater plasticity, as evidenced by higher EL and RA, can endure higher cumulative plastic strain before fracturing, resulting in enhanced LCF performance. Consequently, a source domain model that incorporates material toughness through EL and RA measurements is essential for predicting Δε*_p_* in the target domain and understanding the fatigue behavior it governs.

The Δε*_t_* in the target domain comprises the Δε*_e_* and Δε*_p_*, expressed as Δε*_t_* = Δε*_e_* + Δε*_p_*. The Δε*_e_* correlates positively with material strength, as characterized by YS and UTS, whereas the Δε*_p_* correlates positively with material plasticity, as indicated by EL and RA. As illustrated in Figures S3-4, correlation analysis supports this relationship. Consequently, the four source domain properties (UTS, YS, EL, RA) can be considered static proxies for the corresponding target domain characteristics (FS, Δε*_t_*, Δε*_e_*, Δε*_p_*).


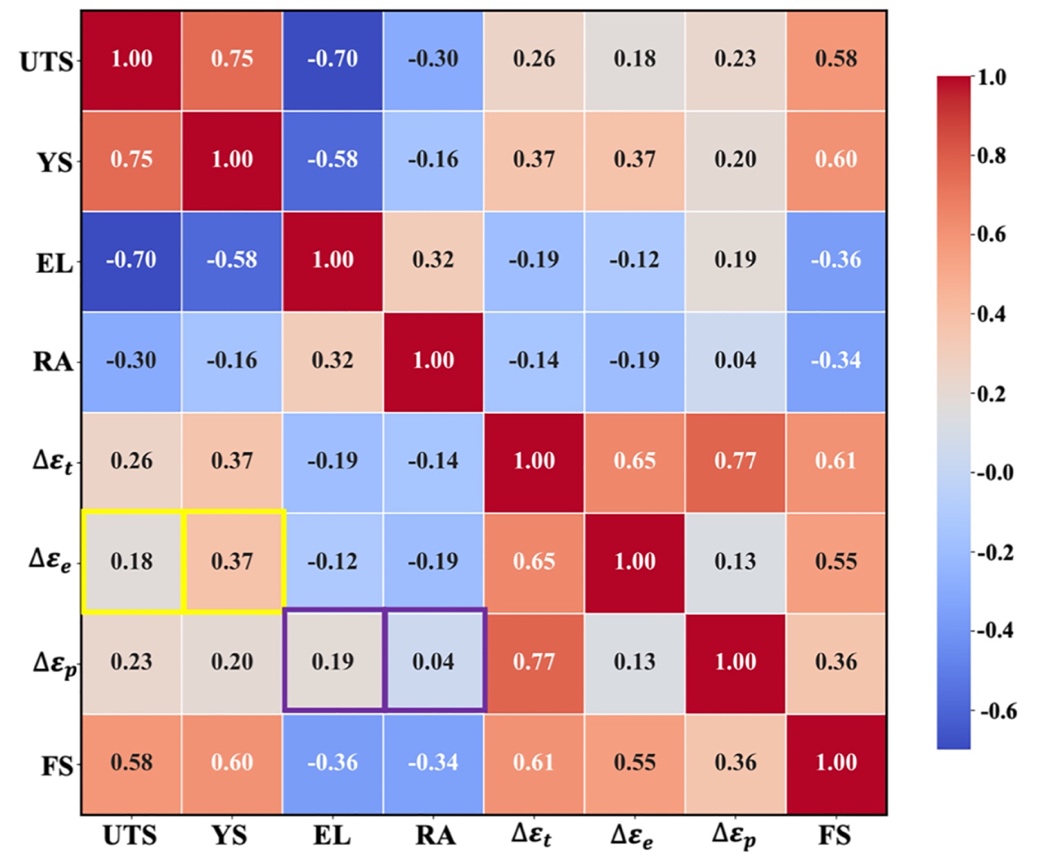


**Figure S3.** Correlation heatmap illustrating the correlation matrix among various features, with color intensity representing the strength and direction of correlations (red denotes positive correlations, blue denotes negative correlations). The highlighted region (yellow box) emphasizes linear relationships among features for detailed examination.


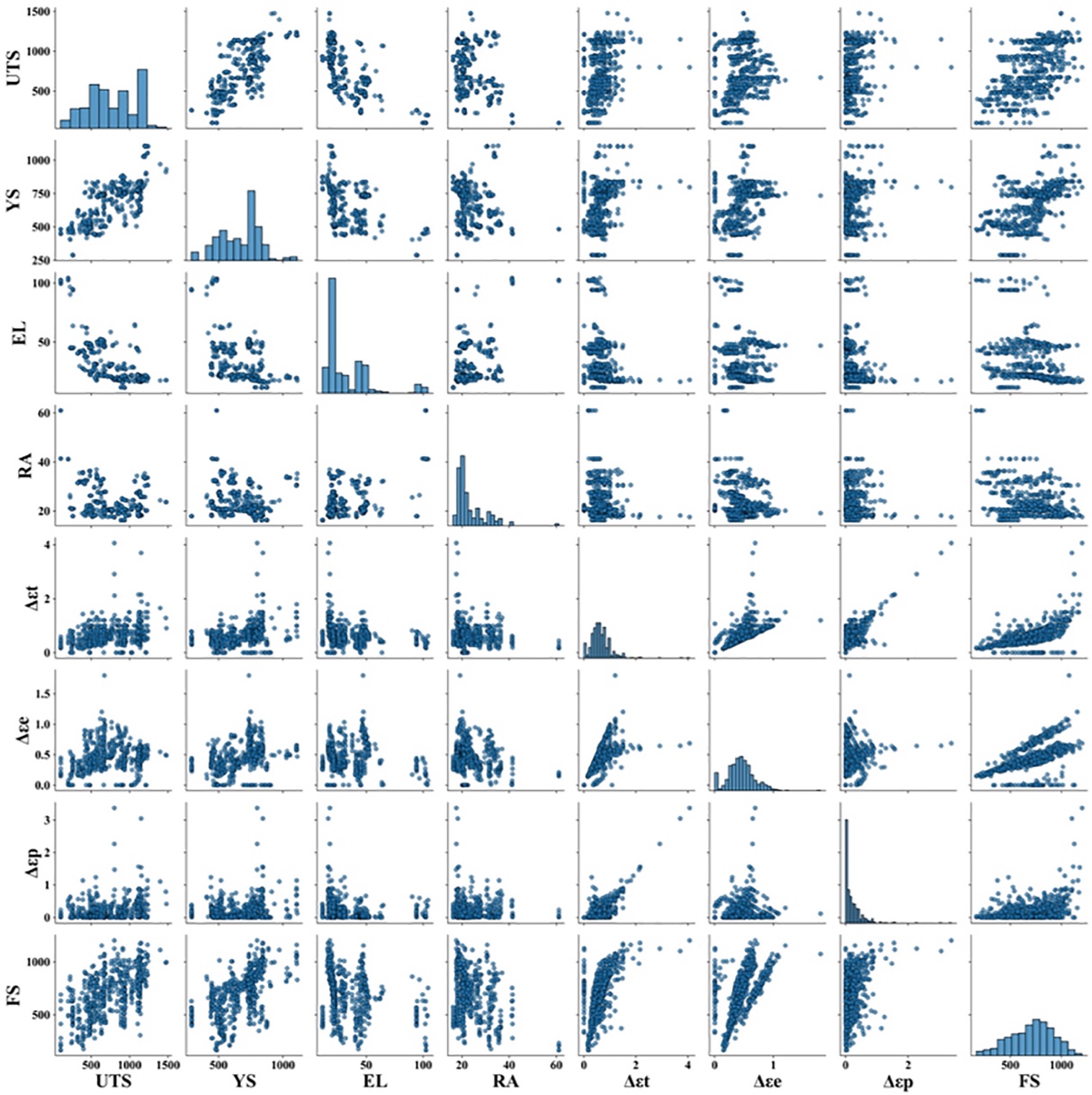


**Figure S4.** The corresponding scatter plots depict pairwise relationships among features, with each subplot illustrating the distribution of data points and potential linear trends.

Despite moderate linear correlations between source and target domains, deeper nonlinear physical couplings may still be present. Our RFR-based TL+PLL model with the STRUT mechanism is specifically designed to capture such nonlinear relationships and actively adapt to interdomain variations. The literature also emphasizes that Pearson’s correlation coefficient measures only linear dependencies. The composition-process-structure-property chain in Ni-based superalloys is inherently nonlinear and involves multifactor interactions. However, we argue that this observation, rather than being a limitation, is precisely the scenario where a sophisticated transfer learning framework like ours becomes indispensable. To prove this, we structured our response in three parts: first, we present a rigorous ablation study to quantitatively demonstrate the significant gains from transfer learning; second, we use Mutual Information (MI)^[9]^ to show that strong nonlinear relationships exist and third, we visualize how our STRUT mechanism actively bridges the domain gap.

To directly and quantitatively validate the effectiveness of our approach, we conducted a rigorous ablation study comparing three models:

1) Model A (Our full TL+PLL model): utilizes transfer learning with the STRUT adaptation mechanism.

2) Model B (Train from scratch): a baseline model trained only on the limited target (fatigue) data.

3) Model C (Naive transfer): a model trained on the source (tensile) data and applied directly to the target data without adaptation.

**
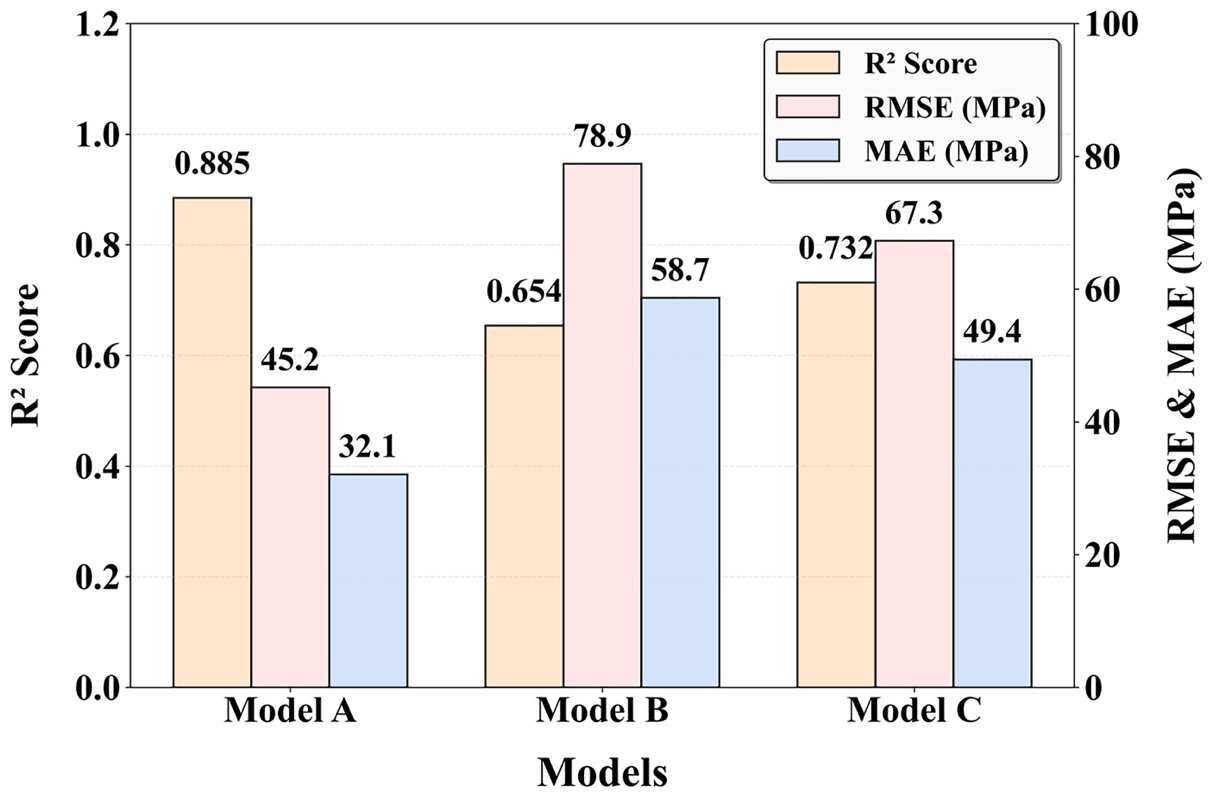
**

**Figure S5.** Quantitative validation of transfer learning and STRUT effectiveness through ablation experiments.

The results of the ablation study, shown in Figure S5, are conclusive. Model A, our full framework, significantly outperforms both baselines. The comparison between Model A and Model B demonstrates that transfer learning provides substantial benefits; the large performance gap confirms that initializing from the source domain offers a much stronger starting point than random initialization, even when linear correlations are only moderate. Furthermore, its superiority over Model C underscores the critical role of our STRUT adaptation mechanism, which effectively bridges the domain-specific gaps implied by these moderate correlations to achieve much higher predictive accuracy.

Having established that transfer learning works, we then explain why. The moderate Pearson correlations only reflect a lack of strong linear relationships. Materials science problems, however, are inherently nonlinear. We therefore employed Mutual Information (MI), a metric that captures both linear and nonlinear dependencies.^[18]^


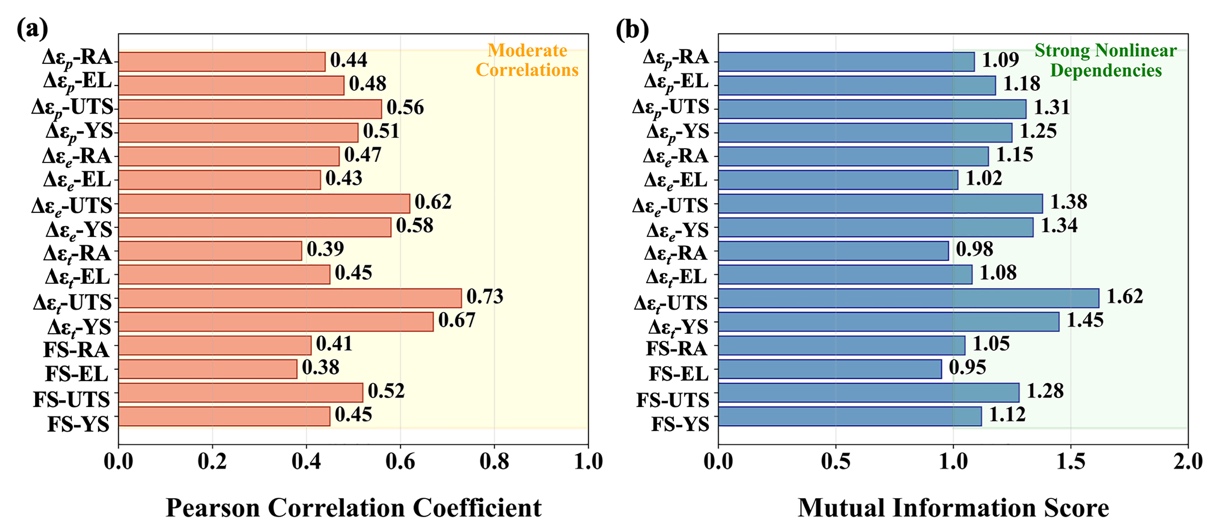


**Figure S6.** Beyond linear correlations: using mutual information (MI) to reveal strong nonlinear dependencies between source and target domain features.

As shown in Figure S6, the feature pairs that exhibited only moderate linear correlation show substantially higher dependence scores under MI analysis. This strongly confirms the existence of robust, predominantly nonlinear physical couplings between the source and target domains. Our Random Forest-based model is specifically designed to capture these complex nonlinearities, which explains its success where simpler linear assumptions might fail.


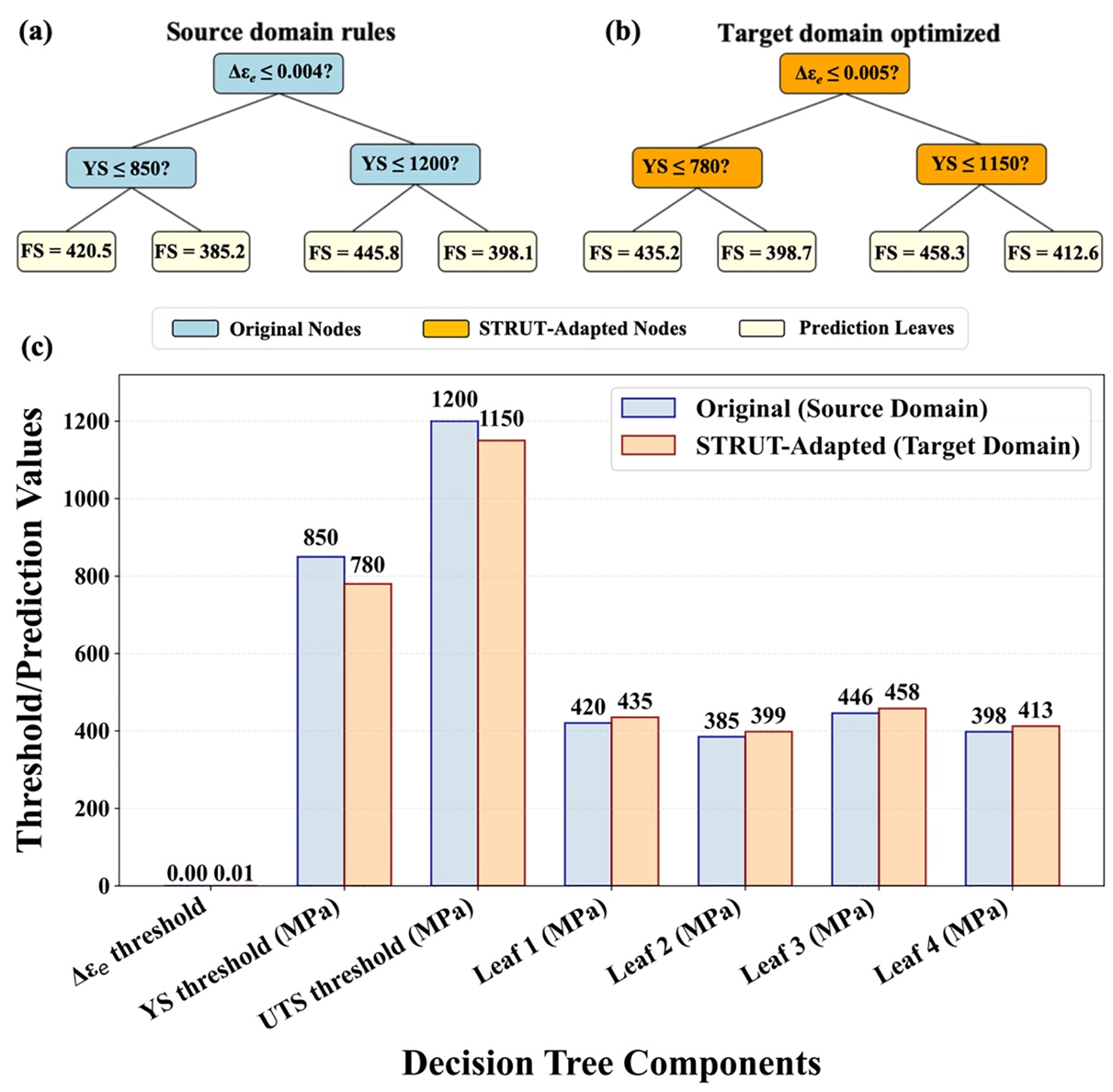


**Figure S7.** Visualization of the STRUT adaptive mechanism: precise cross-domain knowledge calibration through structural adjustments. a) Original Decision Tree: Shows the initial decision rules directly transferred from the source domain, e.g., split thresholds of Δε*_e_* ≤ 0.004 and YS ≤ 850. These rules represent knowledge from the source domain; b) STRUT-Adapted Decision Tree: Shows the same tree after optimization by the STRUT module using limited target domain data. Key split thresholds are adaptively adjusted (e.g., Δε*_e_* from ≤ 0.004 to ≤ 0.005, YS from ≤ 850 to ≤ 780) and leaf-node predictions are updated. Orange nodes highlight modifications introduced by STRUT; c) Quantitative comparison of decision tree components: The bar chart directly quantifies numerical changes in key components (split thresholds and leaf-node predictions) before and after STRUT.

Finally, we visualize how our model intelligently adapts. Figure S7 illustrates the STRUT mechanism in action. It shows that after being initialized with rules from the source domain (e.g., YS ≤ 850), the STRUT module uses the limited target data to adaptively adjust these decision thresholds (e.g., to YS ≤ 780) and update the final predictions.

This dynamic recalibration is the core of our approach. It demonstrates that our model does not blindly apply source domain knowledge. Instead, it intelligently refines that knowledge to fit the specific physics and data distribution of the target domain. This explains why our framework can still perform effectively, even when the relationship between domains is complex and not perfectly linear.

In summary, the moderate linear correlation is not a barrier but the very reason our advanced TL framework is necessary. The quantitative success of our model is rooted in its ability to capture strong nonlinear relationships (confirmed by MI) and to intelligently adapt its internal structure to bridge the gap between domains (visualized via STRUT).

A comparative analysis of the features in the source and target domains revealed shared features, specifically T, ST, STt, STat, Stat, AT and At. Unique features in the source domain include UTS, YS, EL and RA, while the target domain includes FS, Δε*_t_*, Δε*_e_* and Δε*_p_*. To enhance the model applicability to the target domain, the study applied the Structural Transfer Method (STRUT) to optimize the original RFR algorithm.^[10]^


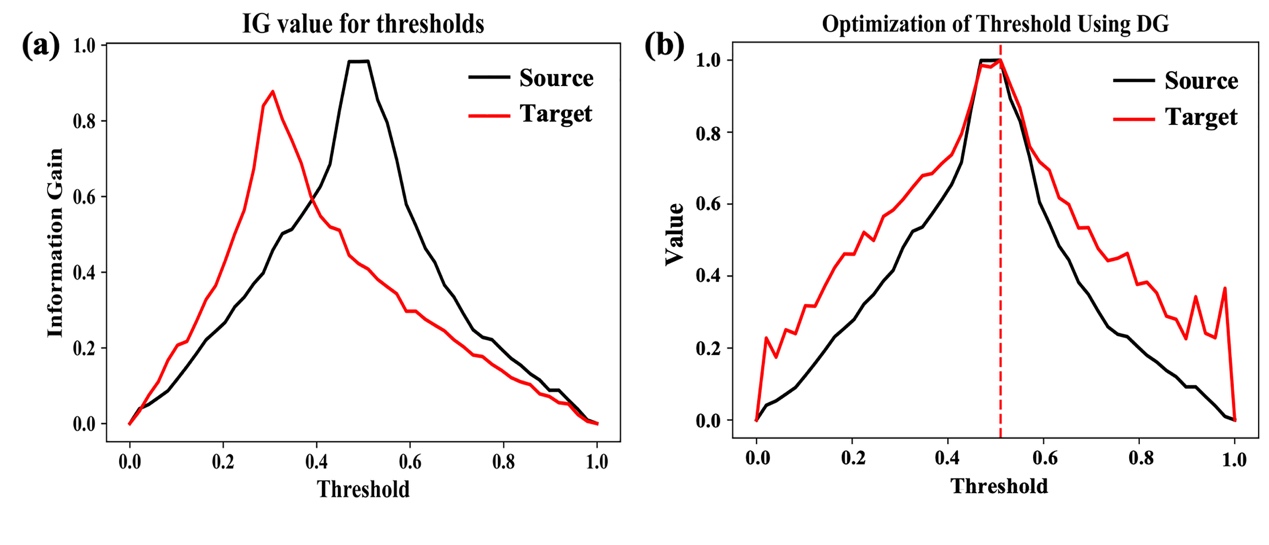


**Figure S8.** Threshold optimization result graph a) IG value for threshold; b) Optimization of threshold using DG.

The STRUT method aims to achieve knowledge transfer from the source domain (Tensile Database) to the target domain (Fatigue Database) via feature adaptation. RFR, the core model of the STRUT method, partitions the feature space by constructing multiple decision trees and adjusts their thresholds based on the distribution of target domain data to improve the adaptability of the model for fatigue performance prediction. The core idea of threshold adjustment is to dynamically optimize the partition point of each decision node by weighting feature importance and statistical properties (mean, variance) of the target domain data to minimize prediction error. Threshold adjustment in RFR is based on Information Gain (IG) optimization combined with the distribution characteristics of target domain data, employing divergence (DG) refinement that uses the difference between source and target domain sample probability distributions on either side of a threshold (Jensen-Shannon divergence) as a metric to fine-tune the threshold and minimize divergence,^[11-13]^ with the IG-DG optimization repeating until the threshold converges or meets a predefined number of iterations. This process dramatically reduces the distribution mismatch caused by the rigid application of source domain thresholds to new domains. This method effectively addresses feature distribution differences between the source and target domains and ensures prediction accuracy on the target domain.

Thresholds learned during source domain training were applied to features present in both the source and target domains. Features unique to a single domain were aligned by scaling their values to a uniform order of magnitude. Standardization is defined as follows:

$x^{'}=\frac{x-\mu}{\sigma}$ (1)

where $x$ is the original eigenvalue, $\mu$represents the mean of the feature, $\sigma$ represents the standard deviation of the feature and $x^{'}$ represents the normalized eigenvalue.

To further reconcile distributional discrepancies between source and target domains, we introduce a Maximum Mean Discrepancy (MMD) regularization term to enhance the generalization of the model on the target domain. Specifically, we add MMD as a penalty to feature representations, where MMD is computed as follow :^[14,15]^

$MMD(x_{s},x_{t})=||\frac{1}{n_{s}}\sum_{i=1}^{n_{s}} \emptyset(x_{s}^{i})-\frac{1}{n_{t}}\sum_{j=1}^{n_{t}} \emptyset(x_{t}^{j})||$ (2)

where $x_{s}$ represents the feature set of the source domain, $x_{t}$ represents the feature set of the target domain, $n_{s}$ represents the number of samples in the source domain, $n_{t}$ represents the number of samples in the target domain and $\emptyset$ represents the kernel function mapping, in this case a Gaussian kernel. This modification encourages the feature distributions from both domains to converge in a reproducing kernel Hilbert space.

We trained an initial RFR on source-domain data and computed the information gain for each feature to determine feature importance rankings. The information gain for feature 𝑓 is given by:^[11]^

$f\left( D \right)=1-\sum_{i=1}^{k} p_{i}^{2}$ (3)

where $D$ is the dataset, $p_{i}$as the proportion of samples belonging to category (i) and $k$represents the number of categories ($k$ = 2). Features are then ordered by descending information gain, providing a basis for subsequent threshold adjustment in the STRUT method.

We analyze the target-domain feature distribution by calculating the mean and standard deviation of each feature, then compare these statistics with those from the source domain to quantify distributional shifts. For each node in the decision tree, we adjust the split threshold using target-domain statistics and optimize the split point according to a divergence-based criterion:^[16,17]^

$H_{w}\left( D \right)=-\sum_{i=1}^{k} w_{i}p_{i}log(p_{i})$ (4)

where $w_{i}$ represents the weight coefficient, $p_{i}$ is the probability of the i category and $k$ is the number of categories. Where $w_{i}$ is the weight adjusted based on the distribution of the target domain, calculated as^[17,18]^:

$w_{i}=\frac{exp(-|\mu_{t}-\mu_{s}|/\sigma_{t})}{\sum_{j=1}^{k} exp(-|\mu_{t}-\mu_{s}|/\sigma_{t})}$ (5)

where $w_{i}$ represents the weight of the i feature, $\mu_{t}$ represents the mean of the feature in the target domain, $\mu_{s}$ represents the mean of the feature in the source domain, $\sigma_{t}$represents the standard deviation of the feature in the target domain and $\mu_{t}-\mu_{s}$ represents the difference in the means of the source and target domains. This iterative refinement minimizes divergence between source and target distributions on either side of the threshold.

To ensure continuous threshold optimization, the STRUT algorithm proceeds iteratively. In each iteration, the algorithm first selects an initial threshold using IG and then further refines it using DG. Detailed results are presented in Figure S8. After multiple rounds of optimization, the thresholds are adjusted to make the decision tree structures of the source and target domains as consistent as possible. This iterative optimization approach minimizes the distributional differences between the source and target domains at each splitting node.

The adjusted model is validated using a limited set of labeled target-domain samples, with detailed results illustrated in Figures 3b-e of the manuscript. The prediction performance of the model is evaluated through cross-validation and the thresholds are iteratively updated until the prediction error in the target domain converges. To clearly illustrate this process, we provide the following Python pseudo-code detailing the core threshold adjustment logic of the RFR in the STRUT method:

def STRUT(X_source, y_source, X_target, y_target):

# 1. Standardized features

X_source_norm = standardize(X_source)

X_target_norm = standardize(X_target)

# 2. Train an initial RFR model in the source domain

model = train_random_forest(X_source_norm, y_source)

# 3. Adjust the threshold for each decision tree

for tree in model.trees:

for node in tree.nodes:

if not node.is_leaf:

feature = node.feature_index

# Obtain initial threshold

threshold = node.threshold

# Select initial threshold using information gain (IG)

threshold=optimize_with_information_gain(X_source_norm, y_source, feature, threshold)

# Further optimize the threshold using Divergence (DG)

threshold = optimize_with_divergence(X_source_norm, X_target_norm, feature, threshold)

# Update node threshold

node.threshold = threshold

# 4. Fine tune the model on the target domain data

while not converged:

y_pred = model.predict(X_target_norm)

error = calculate_error(y_target, y_pred)

# Further adjust feature representation based on MMD regularization

mmd = calculate_mmd(X_source_norm, X_target_norm)

model = update_model_parameters(model, error, mmd)

return model

def optimize_with_information_gain(X, y, feature, threshold):

# Calculate information gain (Eq. 3)

best_threshold = threshold

max_gain = calculate_information_gain(X, y, feature, threshold)

for t in generate_candidate_thresholds(X, feature):

gain = calculate_information_gain(X, y, feature, t)

if gain > max_gain:

max_gain = gain

best_threshold = t

return best_threshold

def optimize_with_divergence(X_source, X_target, feature, threshold):

#Calculate and minimize the distribution difference between the source domain and the target domain (Eqs. 4 and 5)

best_threshold = threshold

min_divergence = calculate_divergence(X_source, X_target, feature, threshold)

mean_source = mean(X_source[:, feature])

mean_target = mean(X_target[:, feature])

std_target = std(X_target[:, feature])

weight = std_target / abs(mean_target - mean_source)

# Try different thresholds and choose the one with the smallest divergence

for t in generate_candidate_thresholds(X_target, feature):

divergence = weight*calculate_jensen_shannon_divergence(X_source, X_target, feature, t)

if divergence < min_divergence:

min_divergence = divergence

best_threshold = t

return best_threshold

The method significantly improves the fit of the model to the target domain data by locally optimizing the thresholds at each decision tree node in the RFR model. Experimental results demonstrate that the optimized model performs excellently in the target domain, validating its robust transfer learning capability.

Similarly, the F-P model is used for forward performance prediction. Its input features consist of nine elements and process parameters, while the output includes four mechanical performance indicators. The model also employs RFR as the optimal algorithm and is trained on the same number of source domain stretching samples. The target domain data also comprises 40 samples. Notably, both the B-P and F-P models employ identical structural transfer methods, ensuring consistency and comparability between them.

To further assess the effectiveness of transfer learning, we conducted a systematic t-SNE (t-distributed stochastic neighbor embedding) analysis of the model’s learned feature embeddings.^[19-21]^ This aimed to visualize the high-dimensional representations learned during the source domain (tensile property database) pretraining phase and examine whether they could be transferred to the target domain (fatigue property database), capturing the underlying physical mechanisms. The key findings are summarized as follows.

First, we visualized the embeddings of both the source (tensile) and target (fatigue) domain data together. As shown in Figure S9a, the two domains are neither completely segregated nor randomly mixed. Instead, they exhibit significant overlap in regions corresponding to materials with similar compositions and properties. This confirms that the model has successfully created a shared latent space, which is the foundational prerequisite for knowledge transfer.

Next, we colored the shared latent space by a fundamental physical property: yield strength (YS). The result in Figure S9b is a physically meaningful gradient, where high-YS samples systematically cluster in one region and low-YS samples in another, with a smooth transition in between. This distinct, organized pattern provides strong evidence that the model has learned to structure its internal representations according to the material's intrinsic strength, a core principle transferable across both tensile and fatigue behavior.

Finally, to provide the most compelling evidence that the model has learned true physical causality, we investigated whether it could distinguish between the two primary strengthening mechanisms in Ni-based superalloys: 1) precipitation strengthening: primarily driven by γ' and γ'' forming elements (Al, Ti, Nb).^[22]^ 2) solid solution strengthening: primarily driven by elements causing lattice distortion (Mo, W, Co).^[22,23]^

Our analysis revealed a finding. As shown in Figures S9c-d, the model has geometrically decoupled these two distinct physical mechanisms within its latent space: samples with high concentrations of precipitation-strengthening elements form a tight, distinct cluster (Figure S9c), which perfectly overlaps with the high-YS region identified in Figure S9b. Crucially, samples dominated by solid solution strengthening form a separate, non-overlapping cluster (Figure S9d).

This geometric separation is the key evidence. It proves the model learned beyond the superficial correlation that 'adding elements makes materials stronger.' Instead, it has independently identified two fundamentally different strengthening mechanisms (precipitation and solid solution) and built separate, transferable representations for each. This ability to distinguish between different physical pathways provides the strongest evidence that the model's knowledge is rooted in physical causality, not mere statistical correlation. Therefore, this hierarchical t-SNE analysis fundamentally clarifies how our transfer learning approach works: by successfully building a physically meaningful latent space and capturing these transferable principles, it ultimately enhances the model's predictive accuracy and generalization.


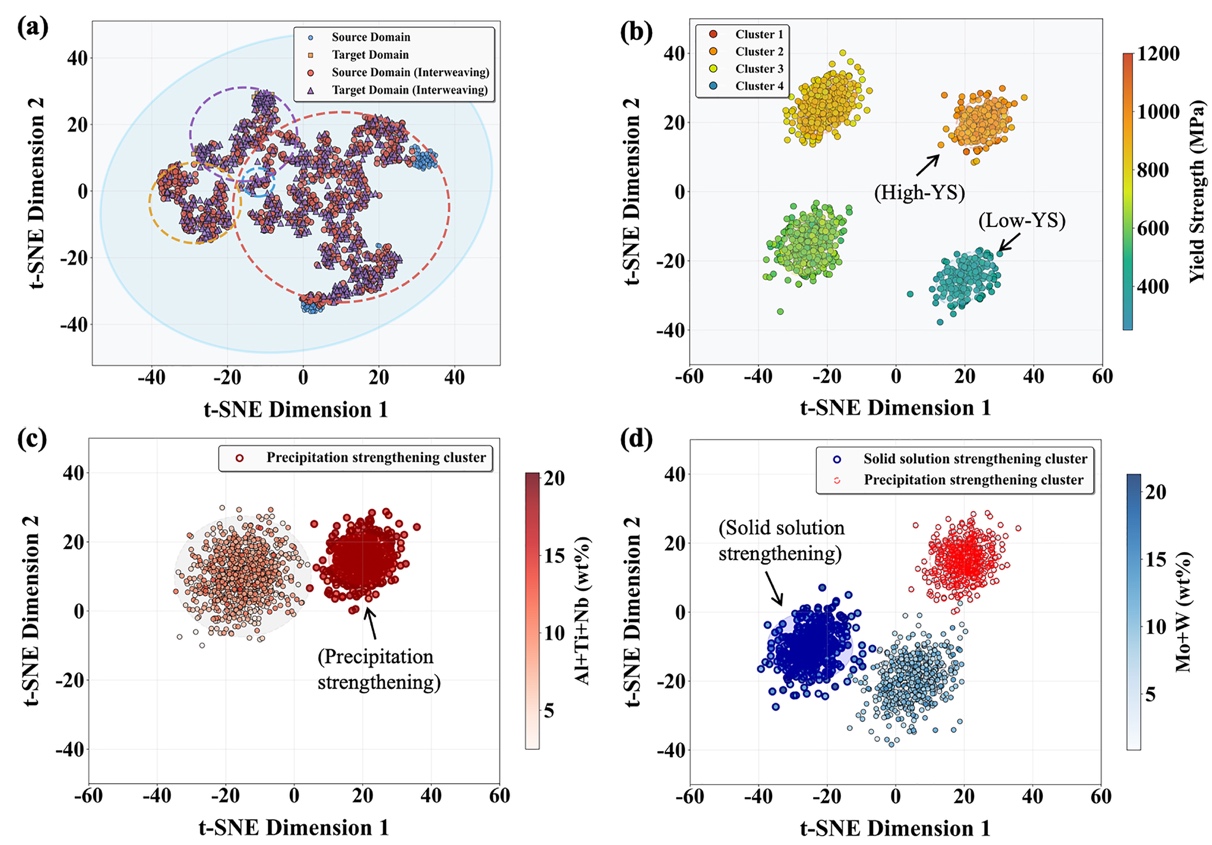


**Figure S9.** Visualization of transferable physical mechanisms learned by the model via t-SNE embedding analysis. a) Joint embedding distribution for source (tensile) and target (fatigue) domains, showing significant overlap; b) The same embedding space color-coded by yield strength, revealing a physically meaningful gradient from low-YS to high-YS; c) The space color-coded by the total content of precipitation strengthening elements (Al+Ti+Nb). A distinct high-concentration cluster emerges, overlapping with the high-YS region; d) The space color-coded by the total content of solid solution strengthening elements (Mo+W). A separate high-concentration cluster forms, demonstrating the model's ability to geometrically decouple the two distinct strengthening mechanisms.

**Table S2.** 30 new data chemical composition of alloys.

| Alloy composition(wt.%) | | | | | | | | |
| --- | --- | --- | --- | --- | --- | --- | --- | --- |
| Ni | Cr | Co | Fe | Al | Ti | Nb | Mo | W |
| 58.55 | 19.18 | 0.766 | 4.149 | 1.319 | 3.03 | 2.44 | 3.51 | 1.24 |
| 59.34 | 18.3 | 12.7 | 1.06 | 1.37 | 2.84 | 0 | 4.27 | 0 |
| 56.18 | 16.0 | 13.0 | 0.00 | 2.2 | 3.8 | 0.7 | 4 | 4 |
| 52.3 | 18.18 | 0 | 19.13 | 1.24 | 0.39 | 5.16 | 3.01 | 0 |
| 53.0 | 19.0 | 0 | 13.85 | 0.7 | 1.1 | 4.8 | 7.5 | 0 |
| 58.2 | 15.52 | 14.44 | 0 | 2.55 | 4.98 | 0.00 | 3.02 | 1.18 |
| 51.82 | 18.94 | 0.03 | 19.35 | 0.59 | 1 | 5.23 | 3.01 | 0 |
| 60.42 | 18.95 | 6.94 | 0.4 | 2.06 | 1.29 | 0 | 3.96 | 5.94 |
| 60.60 | 18.5 | 6.5 | 1.00 | 2.1 | 1.25 | 0 | 4 | 6 |
| 62.61 | 12.98 | 8 | 0 | 3.48 | 2.55 | 3.5 | 3.4 | 3.4 |
| 51.20 | 18.77 | 10.88 | 4.5 | 1.52 | 3.1 | 0 | 9.87 | 0 |
| 57.62 | 19.2 | 13.5 | 0.52 | 1.41 | 3.25 | 0 | 4.45 | 0 |
| 57.83 | 19.14 | 13.34 | 0.47 | 1.46 | 3.24 | 0 | 4.42 | 0 |
| 53.0 | 19.0 | 0 | 18.15 | 0.5 | 1.0 | 5.3 | 3 | 0 |
| 52.82 | 18.96 | 0 | 18.36 | 0.59 | 1.0 | 5.23 | 3.01 | 0 |
| 57.17 | 16.0 | 15.0 | 0 | 2.5 | 5 | 0.00 | 3.00 | 1.25 |
| 51.20 | 18.77 | 10.88 | 4.5 | 1.52 | 3.1 | 0 | 9.87 | 0 |
| 60.42 | 18.95 | 6.94 | 0.4 | 2.06 | 1.29 | 0 | 3.96 | 5.94 |
| 74.09 | 20.45 | 0 | 0 | 0.98 | 2.87 | 1.57 | 0 | 0 |
| 56.77 | 15.7 | 12.9 | 0.09 | 2.09 | 3.73 | 0.8 | 3.94 | 3.98 |
| 57.17 | 16.0 | 15.0 | 0 | 2.5 | 5 | 0 | 3.0 | 1.25 |
| 57.22 | 16.3 | 14.8 | 0 | 2.4 | 4.8 | 0 | 3.01 | 1.25 |
| 53.22 | 18.92 | 0 | 17.6 | 0.56 | 1.0 | 5.35 | 3.3 | 0 |
| 59.36 | 15.83 | 8.28 | 4.15 | 2.31 | 3.44 | 1.10 | 2.86 | 2.6 |
| 57.64 | 15.93 | 14.56 | 0 | 2.51 | 5.02 | 0 | 3.02 | 1.24 |
| 59.345 | 18.30 | 12.77 | 1.06 | 1.37 | 2.84 | 1.37 | 2.84 | 0 |
| 57.62 | 19.2 | 13.5 | 0.52 | 1.41 | 3.25 | 0 | 4.45 | 0 |
| 58.85 | 19.31 | 13.39 | 0 | 1.46 | 2.64 | 0 | 4.3 | 0 |
| 57.57 | 19.21 | 14.2 | 0.15 | 1.37 | 2.97 | 0 | 4.42 | 0 |
| 56.77 | 15.7 | 12.9 | 0 | 2.1 | 3.7 | 0.7 | 4 | 4 |

**Table S3.** 10 new data chemical composition of alloys.

| Alloy composition(wt.%) | | | | | | | | |
| --- | --- | --- | --- | --- | --- | --- | --- | --- |
| Ni | Cr | Co | Fe | Al | Ti | Nb | Mo | W |
| 58.55 | 10.25 | 15 | 0 | 4.05 | 1.65 | 0 | 5 | 5.5 |
| 75.63 | 20.46 | 0 | 0.02 | 0.96 | 2.92 | 0 | 0.01 | 0 |
| 58.62 | 18.92 | 0 | 17.6 | 0.56 | 1 | 0 | 3.3 | 0 |
| 54.23 | 18.96 | 0 | 19.94 | 0.7 | 1.07 | 5.1 | 0 | 0 |
| 52.26 | 19.2 | 0 | 18.7 | 0.5 | 1.1 | 5.2 | 3 | 0 |
| 71.36 | 19.05 | 0 | 0 | 0.5 | 0.92 | 5.17 | 3 | 0 |
| 51.82 | 18.94 | 0.03 | 19.35 | 0.59 | 1 | 5.23 | 3.01 | 0 |
| 57.66 | 15.83 | 14.75 | 0 | 2.64 | 4.95 | 0 | 2.93 | 1.24 |
| 73.962 | 20.24 | 0.008 | 0.45 | 0.97 | 2.85 | 1.52 | 0 | 0 |
| 55.84 | 16.46 | 13.07 | 0 | 2.02 | 3.64 | 0.76 | 4.09 | 4.12 |


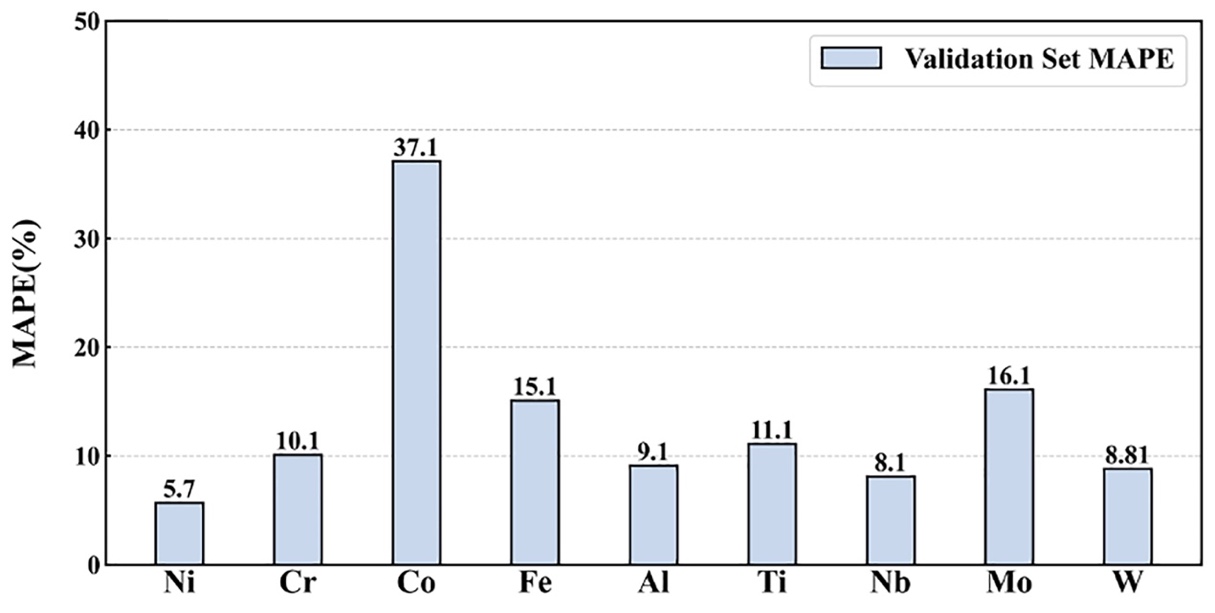


**Figure S10.** Detailed MAPE values during validation of new data.


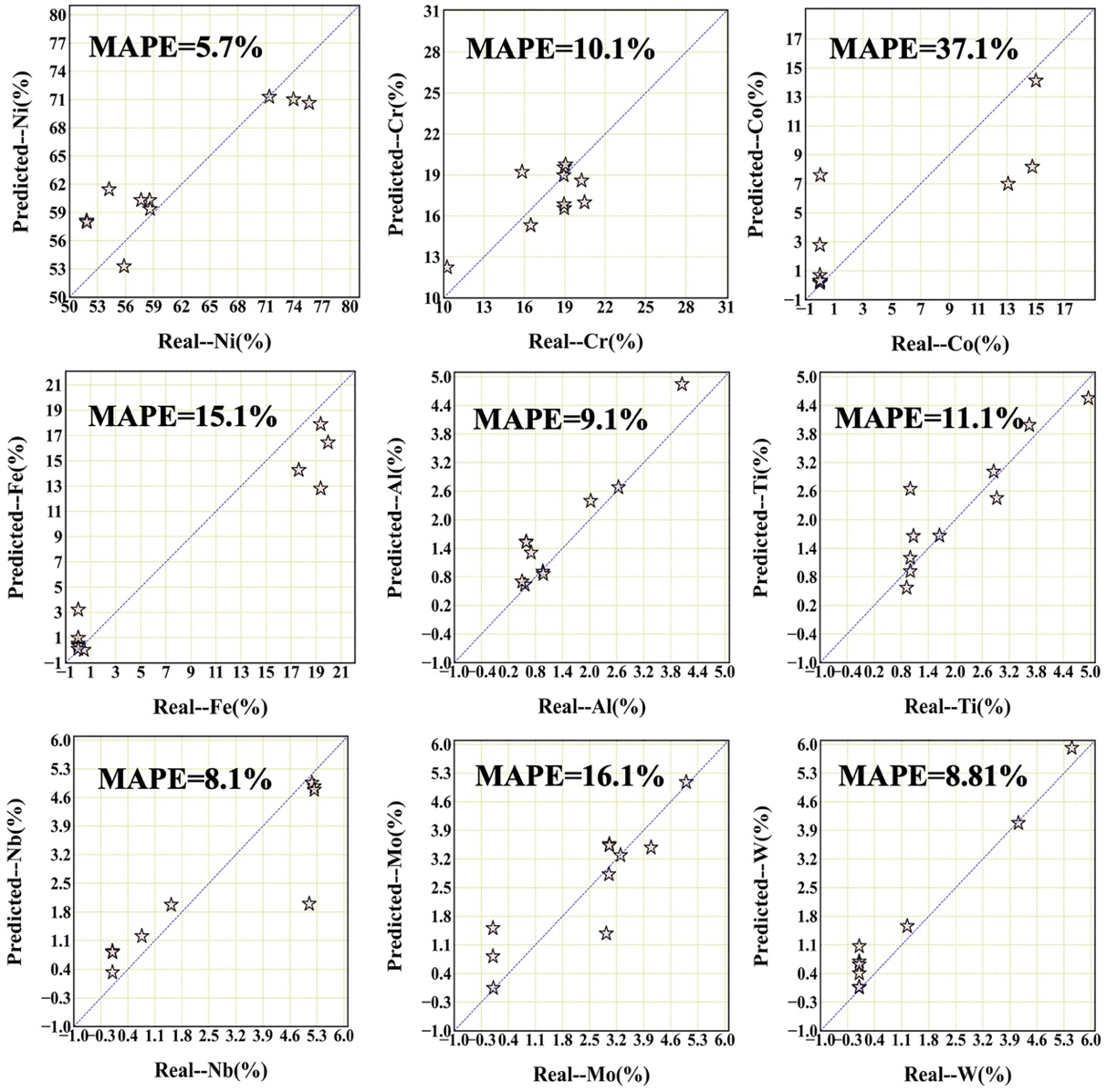


**Figure S11.** Detailed MAPE values during validation of new data.


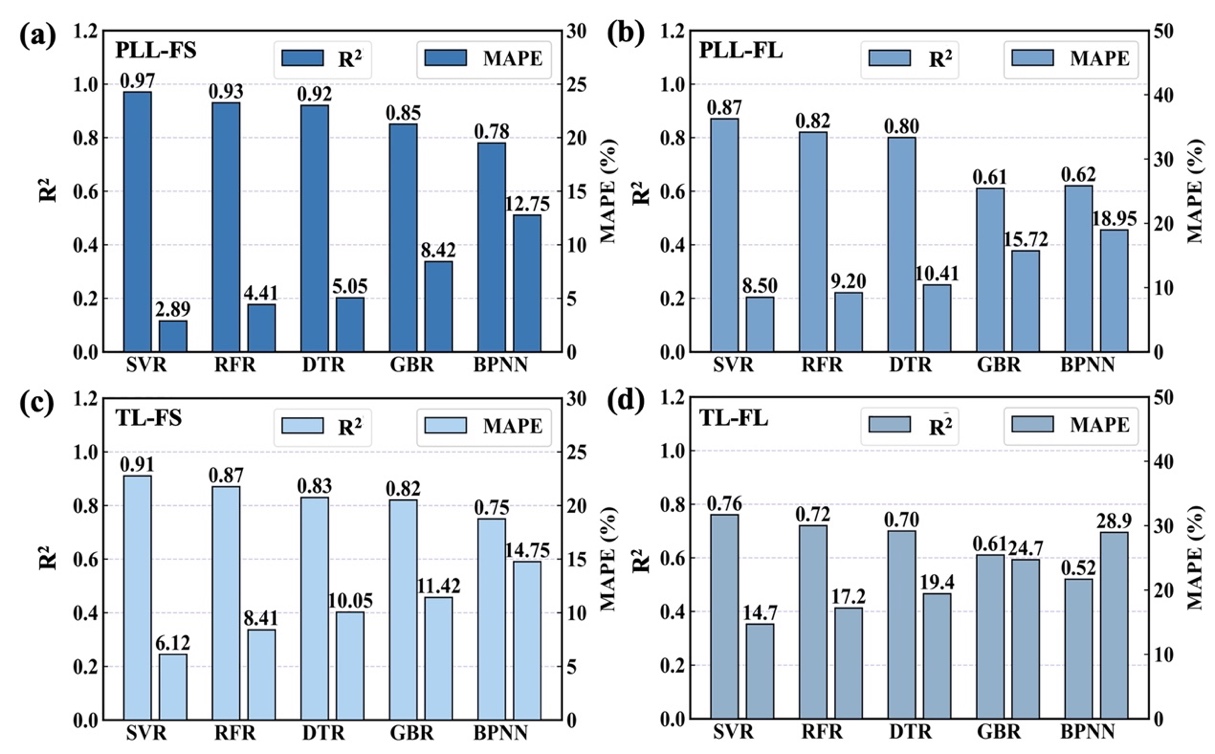


**Figure S12.** R² and MAPE values for each algorithmic model in predicting fatigue performance, before and after applying PLL. a) and b) show results after PLL application, while c) and d) show results before PLL application.

**3. Threshold Optimization**

In the process of PLL model construction and loop optimization, threshold selection plays a crucial role in data screening, model improvement and the optimization of predictive capability. The setting of the threshold is closely linked to the alloy grades of the true labels and noisy labels. If the threshold value is too high, although it can ensure that all alloy grades in the noisy labels are present in the true label samples, it will lead to the inclusion of low-quality or biased samples in the true label set, which can compromise the reliability of the dataset and may negatively impact model performance. Conversely, setting the threshold too low reduces the number of true labels and the corresponding alloy grades required for effective validation and Gaussian distribution model learning, ultimately reducing guidance for failed data. Therefore, threshold selection must follow a systematic optimization strategy and avoid arbitrary determination.

At the initial stage, thresholds are established based on empirical values derived from relevant studies.^[24]^ For the initial modeling of material properties, the error threshold *e₁* = 5 is considered valid. To improve model accuracy, narrow the error range and investigate the intrinsic connection between alloy compositions and properties, this study tested different thresholds (*e₁* = 1, 2, 3, 4, 5) in turn and evaluated the distribution differences of alloy grades between true labels and noisy labels at each threshold. By comparing these distribution differences, the most appropriate threshold setting was initially determined. Subsequently, based on the optimized alloy compositions under this threshold, the T*_F-P_* L model was constructed and its performance was evaluated.

The optimization process is as follows: at the initial stage, in the PLL strategy, by comparing the alloy grades corresponding to the true label samples with the alloy grades in the fatigue database under different thresholds (*e₁*=5, 4, 3, 2, 1), it was observed that when the thresholds were *e₁*=5, 4 and 3, the alloy grades of the true label samples matched those in the fatigue database, totaling 30 alloy grades. For thresholds *e₁*=2 and *e₁*=1, the corresponding alloy grades were reduced due to the decrease in the number of true label samples.

Since subsequent cycle optimization requires true label samples to guide noisy label samples, it is critical to ensure that the number of alloy grades in the true labels aligns with the fatigue database. In particular, when the alloy grades in the true labels do not encompass the key alloy classes in the fatigue database, the optimization process may not effectively guide the adjustment of the new noisy labels. Ensuring that the optimized data matches the structure of the database by aligning alloy grades can increase the stability of the optimization results, reduce data bias and improve model reliability.

To determine the optimal threshold, a sensitivity analysis was conducted. Based on the criterion that “true labels must cover all 30 alloy grades,” the candidate thresholds were limited to *e₁* = 3, 4, and 5. Subsequently, for each candidate threshold (*e₁* = 5, *e₁* = 4 and *e₁* = 3), the complete iterative optimization process was independently executed to process the data. A prediction model was subsequently constructed using the optimized data. Finally, the performance of the final models under these three threshold settings was evaluated using cross-validation, as shown in Supplementary Figure S13. The comparison results indicate that the model achieves optimal performance across key metrics, including R² and MAPE, when the threshold is set to *e₁* = 3.


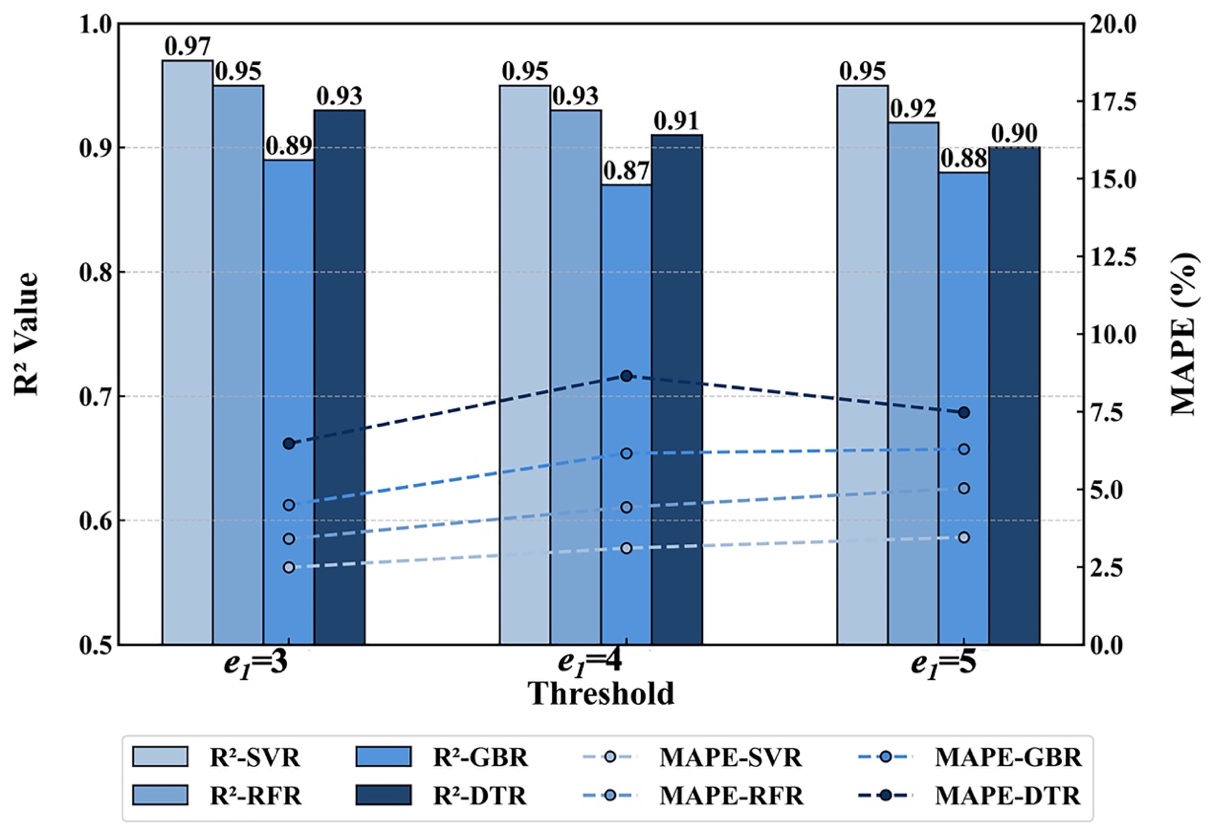


**Figure S13.** illustrates the overall R² values and metrics of alloy compositions at the end of the cycle optimization for various algorithmic models at different threshold settings.

**4. Details of PLL**


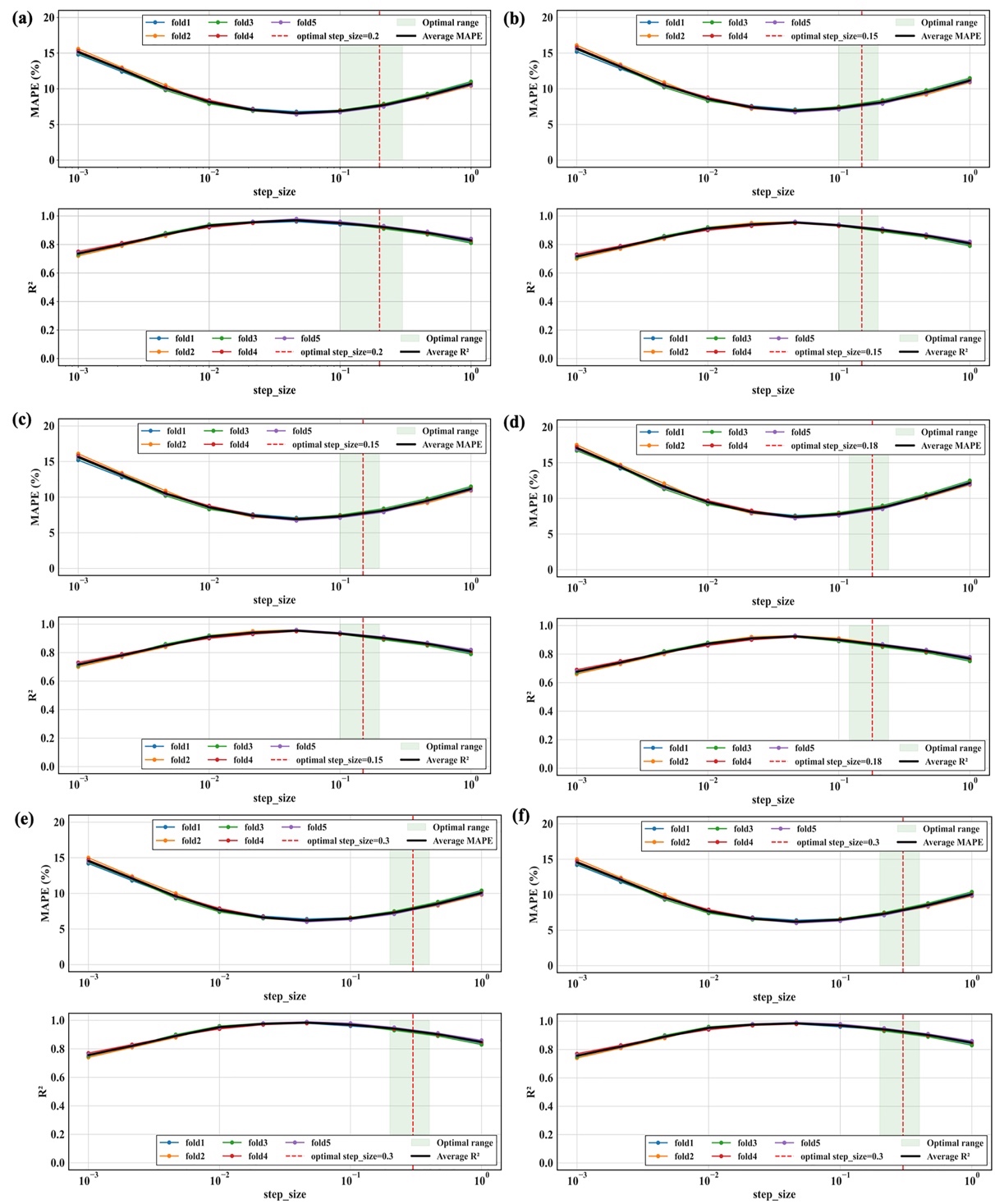


**Figure S14.** a)-f) Respectively display the optimal *step_size* values obtained after five-fold cross-validation from optimization rounds one through six. The horizontal axis represents the *step_size* values, while the vertical axis indicates the corresponding model prediction evaluation R² and MAPE values.

The generation of perturbation vector Δ in Eq. (1) is based on a Gaussian distribution model, wherein the selection of the *step_size* parameter follows scientific principles rather than arbitrary assignment. We developed a dynamic adjustment strategy based on the mean prediction error of each compositional element, establishing the *step_size* inversely proportional to error magnitude. This approach ensures reasonable weight allocation for the contribution of each composition to the optimization process. For elements with larger errors, smaller *step_size* values are implemented to ensure accurate fitting, while elements with smaller errors utilize larger *step_size* values to accelerate optimization.^[25,26]^ The *step_size* was dynamically adjusted within the range of [0.001, 1.0]. Additionally, we optimized the *step_size* through five-fold cross-validation in conjunction with grid search methodology, evaluating model stability using metrics such as MAPE and R². Comprehensive results are presented in Supporting Figure S14. Experimental results demonstrate that the error-based dynamic adjustment strategy significantly enhances the ability of the model to fit complex composition-property relationships, a finding corroborated by other studies.^[24]^


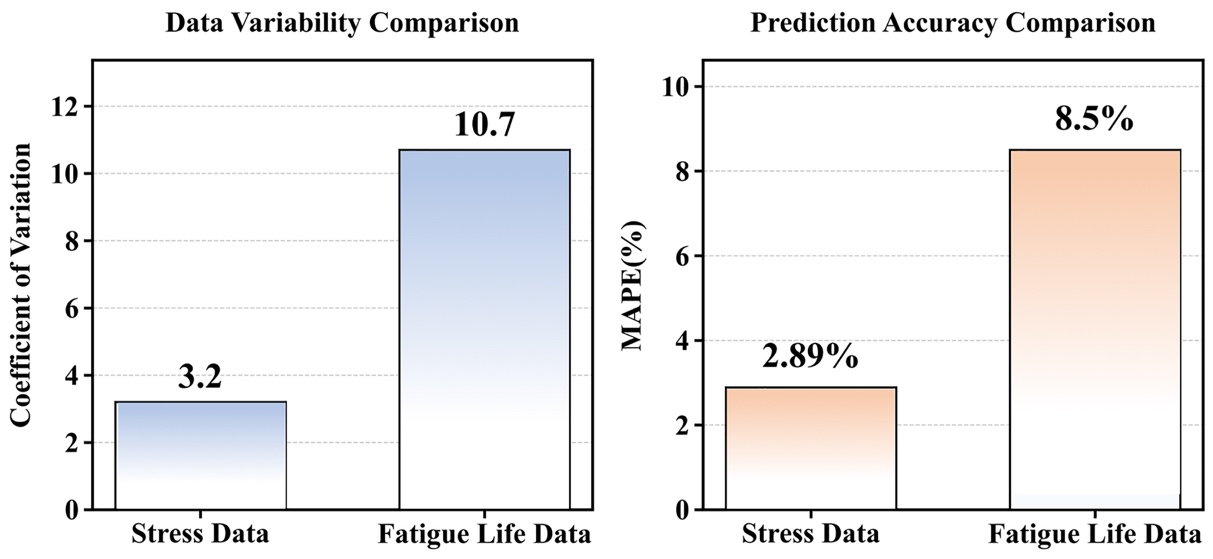


**Figure S15.** Presents the data analysis of fatigue stress and fatigue life. a) Presents a comparison of data variability; b) Presents the MAPE evaluation index of the optimal algorithm model for fatigue stress and fatigue life.


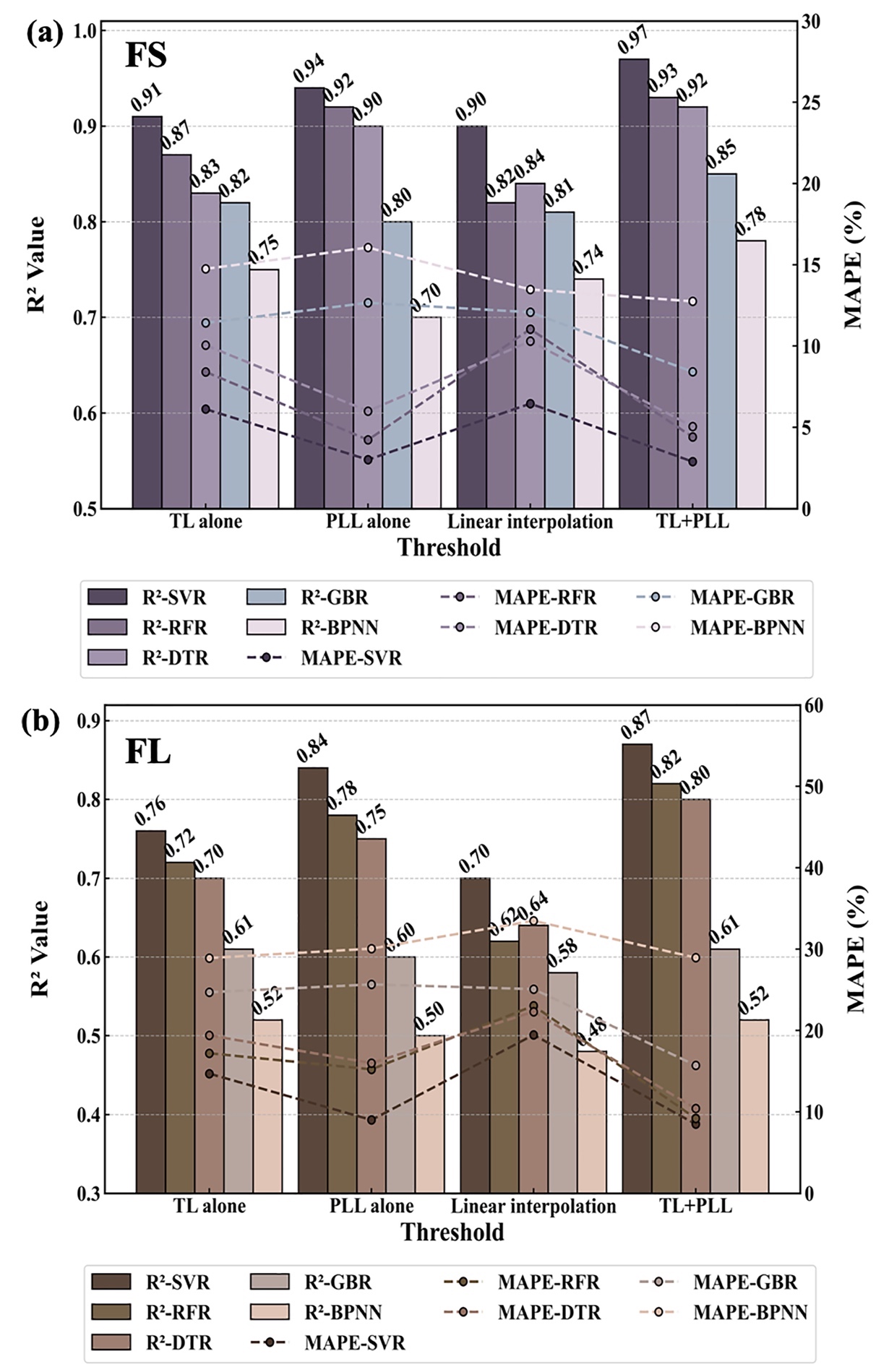


**Figure S16.** Presents a comparison between the TL+PLL method and baseline methods, showing the overall R² values and MAPE evaluation metrics of alloy compositions for various algorithmic models at the end of the cycle optimization.

To thoroughly evaluate the performance advantages of the TL+PLL method, systematic comparative experiments were conducted with three benchmark methods: (I) The standalone transfer learning method; (II) The standalone partially labeled learning method and (III) The standalone traditional linear interpolation method. Detailed experimental procedures are presented in the *Supporting Information.*

(I) Standalone transfer learning method: this approach employs a pure transfer learning strategy to predict fatigue performance. The specific implementation steps are: train the prediction model using the Tensile Database and then directly transfer the trained model to the Fatigue Database for fatigue performance prediction.

(II) Standalone partially labeled learning: this method specifically addresses the problem of composition range uncertainty in the Fatigue Database. In the PLL framework, the composition range of each sample is modeled as a set of candidate labels. For example, when the Ni content range is 50-55 wt%, all possible values within this range constitute the candidate label space. Considering the computational complexity of continuous values, we discretize the composition ranges into equally spaced sampling points (with an interval of 0.1 wt%) to form a finite set of candidate labels. The PLL model selects the optimal labels by calculating the contribution of each candidate label to the target variable using a weighted voting mechanism and subsequently obtains the specific composition values for training the fatigue performance prediction model.

(III) Standalone traditional linear interpolation method: as a classical benchmark, linear interpolation is used to estimate the specific composition values in the Fatigue Database, after which the fatigue performance prediction model is trained based on the estimated values.

To ensure the fairness and reliability of the comparison, all methods employ the same evaluation metrics, including the coefficient of R² and MAPE. The detailed results shown in Figure S16 indicate that the TL+PLL method demonstrates superior performance across all evaluation metrics. Specifically, for fatigue strength prediction, the TL+PLL method achieves an R² of 0.97 and a MAPE of 2.89%, respectively; for fatigue life prediction, the corresponding metrics are 0.87 and 8.50%, respectively, which represent significant improvements over other benchmark methods.

Detailed analysis reveals that the superiority of the TL+PLL method stems from its unique dual advantages: on the one hand, the transfer learning composition effectively utilizes the abundant tensile performance data to provide valuable a priori knowledge for fatigue performance prediction; on the other hand, the partial label learning composition effectively addresses the inherent ambiguity of the composition ranges to provide accurate predictions despite limited data and uncertain conditions. In contrast, each benchmark method has inherent limitations: standalone transfer learning method cannot effectively handle ambiguous composition range data;^[27]^ standalone partially labeled learning method lacks the support of external knowledge sources and exhibit limited prediction accuracy;^[28]^ and standalone traditional linear interpolation methods are oversimplified and fail to capture the complex nonlinear relationships between material composition and properties.^[29]^ Consequently, all of these methods demonstrate inferior performance when confronted with the complexity and uncertainty of material data.

Unlike traditional or streamlined UQ methods, the core task of PLL extends beyond quantifying uncertainty to actively resolving ambiguity-identifying the true label within a set of candidates that best represents the underlying physical state. This capability constitutes a fundamental distinction from standard UQ approaches.

To address this, we conducted a direct comparative study. We benchmarked our TL+PLL framework against three simpler, widely-used methods for handling ambiguous interval data:

1) Baseline midpoint imputation (BMI)^[30]^: uses the midpoint of the interval (e.g., (min+max)/2) as a deterministic input.

2) Baseline sampling-ensemble imputation (BSEI)^[31]^: averages predictions from multiple models trained on random samples drawn from the intervals.

3) Interval prediction (IP): trains a model to predict a performance range rather than a single point value.^[31]^


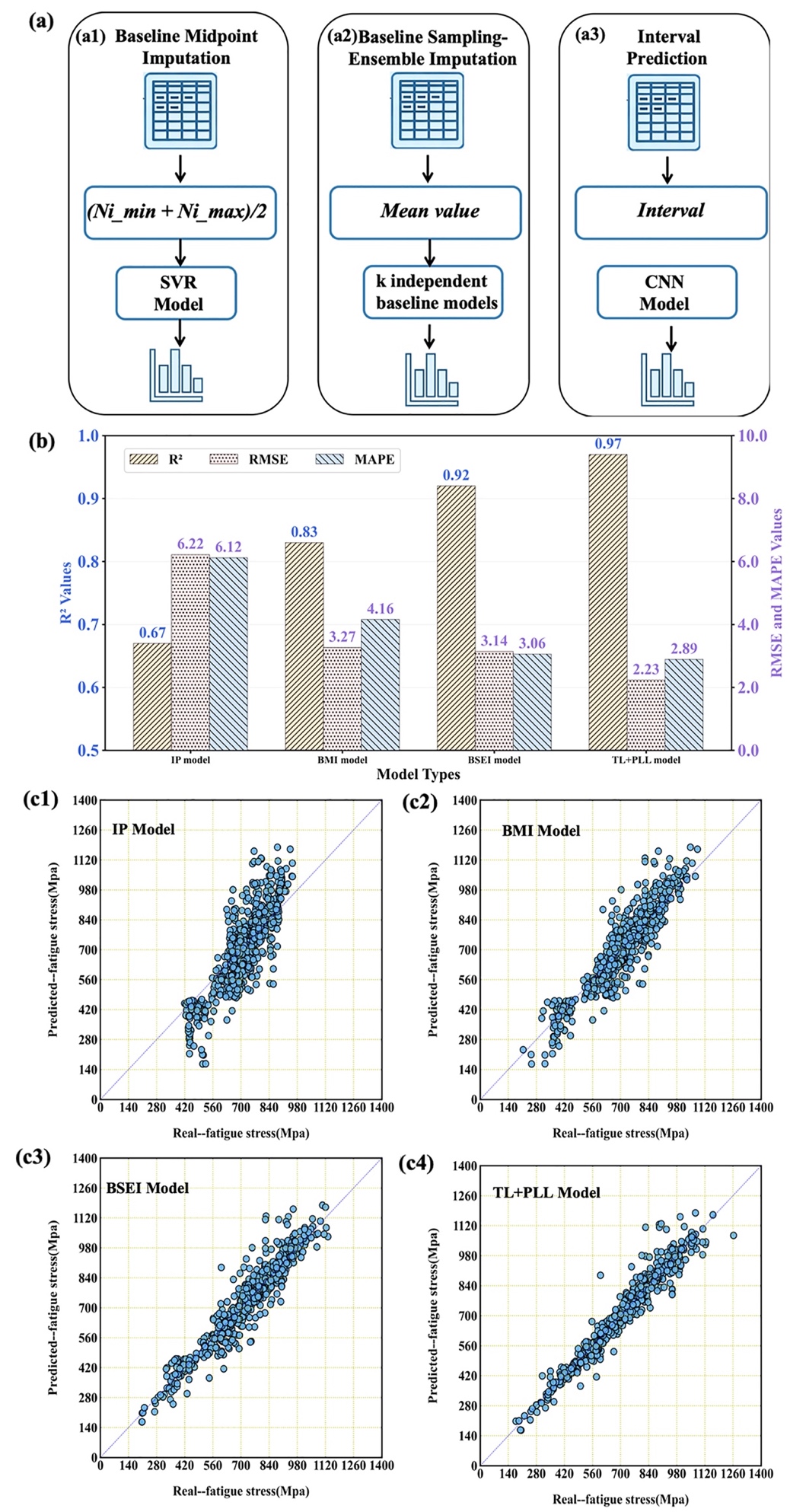


**Figure S17.** Performance comparison of the TL+PLL framework with three baseline methods for handling ambiguous composition data. a) Schematics of the baseline methods: a1) Midpoint Imputation (BMI), a2) Sampling-Ensemble Imputation (BSEI), and a3) Interval Prediction (IP); b) Quantitative comparison of R², RMSE and MAPE for all four models, showing the superior performance of the TL+PLL framework; c) Scatter plots of predicted vs. true values, visually confirming that the TL+PLL model provides the most accurate predictions.

The results in Figure S17 clearly demonstrate the superior performance of the TL+PLL framework. Quantitatively (Figure S17b), the method achieves an R^2^ of 0.97, significantly outperforming BMI (0.83) and BSEI (0.92) while also delivering the lowest prediction errors (RMSE and MAPE). This numerical advantage is visually confirmed in the scatter plots (Figure S17c): predictions from the TL+PLL model show the tightest clustering around the ideal diagonal line, whereas other methods exhibit considerably more scatter and deviation. The lower point-prediction accuracy of the IP method is expected, given its different objective of predicting an interval. These results highlight a fundamental difference in approach. While simpler methods like BMI and BSEI passively handle uncertainty by averaging or sampling, our PLL framework actively resolves ambiguity. It iteratively infers the most plausible composition value within the interval that best explains the observed fatigue performance. This active inference capability allows it to capture complex, non-linear composition-property relationships that simpler statistical approaches miss. For instance, BMI is prone to systematic bias if the true value is not at the interval's center, while BSEI fundamentally propagates sampling noise into its final predictions.

In summary, our experiments confirm that while simpler UQ methods can handle ambiguous data to some extent, our TL+PLL framework is demonstrably more accurate because it is designed to infer the latent ground truth rather than simply manage the uncertainty. This superior performance comes with a trade-off in computational complexity. The iterative optimization process of PLL is more demanding than a single-pass method like BMI. However, we argue this additional complexity is a necessary investment. For applications like materials design and optimization, which require precise compositional targets, the ability of our framework to provide a specific, highly plausible component value rather than an interval or a stochastic estimate-offers a unique and indispensable advantage.

**5. Detailed comparative experiments of feature strategies**

To rigorously validate the impact of eliminating feature correlations on model accuracy, a novel multi-level experimental framework was designed. This framework systematically compares the performance of the baseline "retain-all-features" model against three widely-used strategies for feature selection and dimensionality reduction:

1) Pearson correlation threshold: This strategy systematically removes one feature from any pair with a Pearson correlation coefficient greater than 0.5. It serves to test the effect of a straightforward feature reduction based on linear correlation.

2) Variance inflation factor (VIF) elimination^[32]^: this more rigorous method addresses multicollinearity. Features were iteratively removed based on the highest VIF score until all remaining features had a VIF below the established threshold of 5.0.

3) Principal component analysis (PCA): this strategy transforms the original features into linearly independent principal components rather than removing them. Components sufficient to explain 95% of the total variance were retained for modeling.

4) Baseline (Retain all features): this model was trained using the complete set of original features. It serves as the primary control against which the performance of the feature-reduction strategies is evaluated.


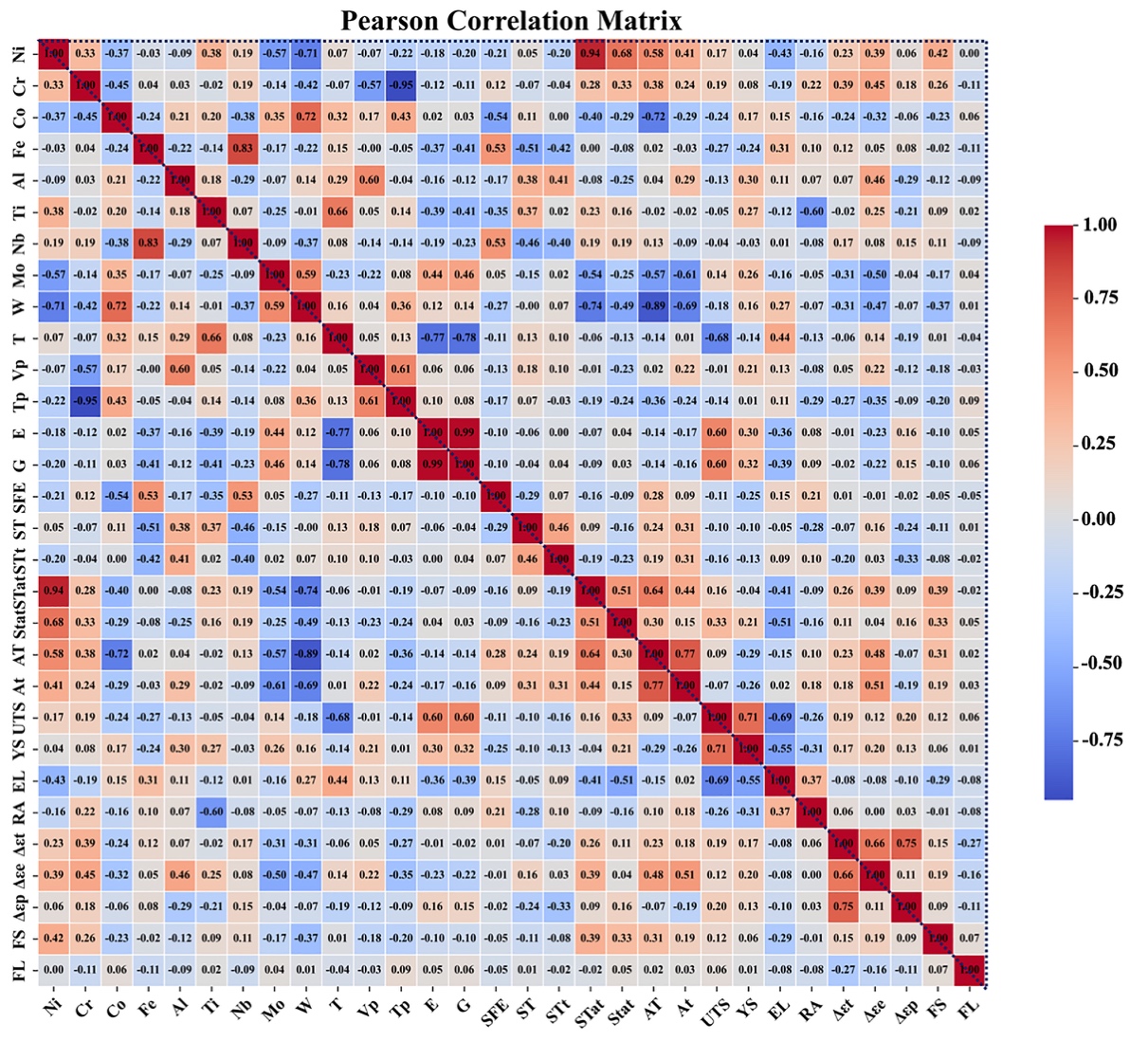


**Figure S18.** Pearson correlation matrix heatmap illustrating correlations among features, with the color gradient (red denoting positive correlations, blue denoting negative correlations) indicating the strength of the correlations.


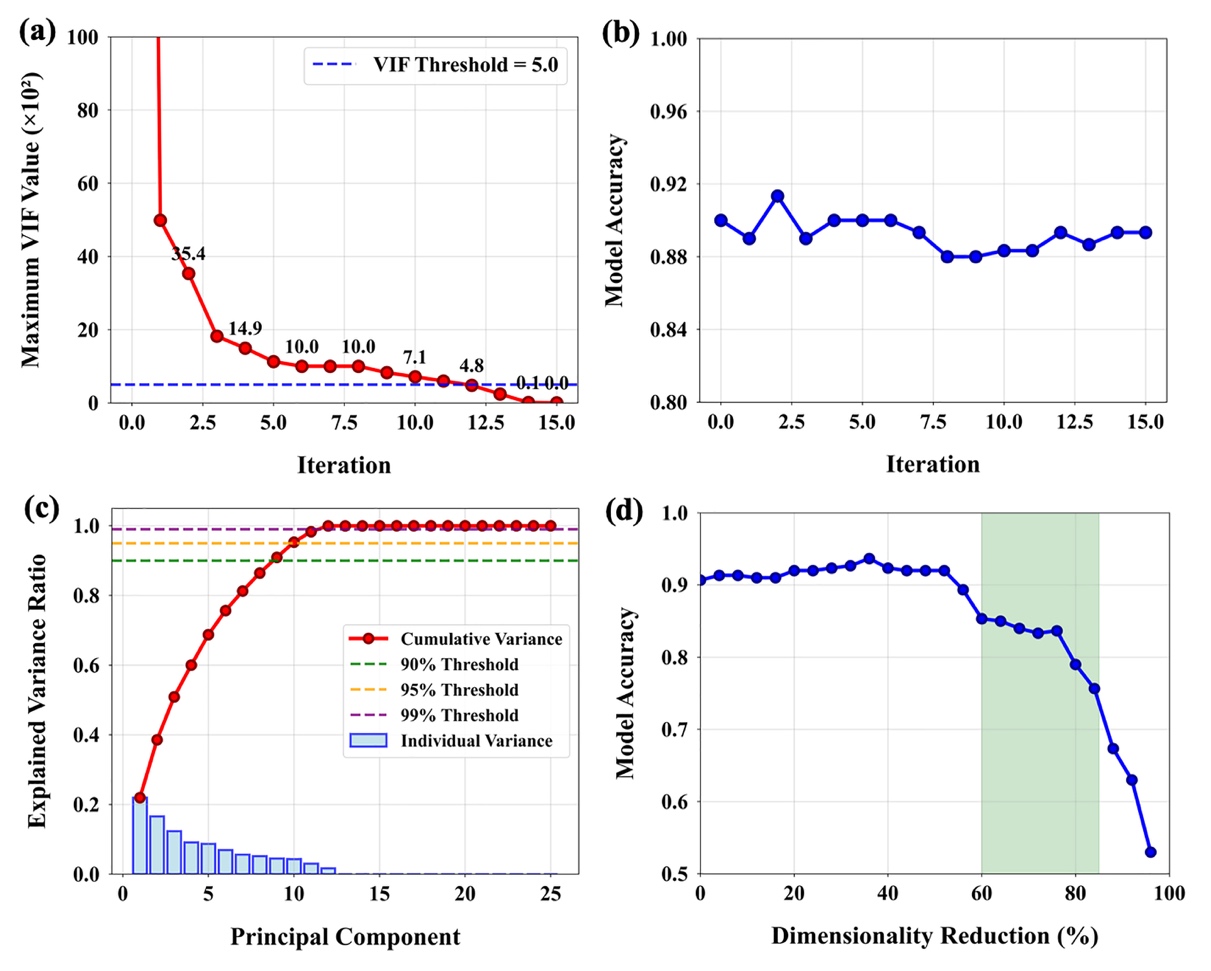


**Figure S19.** Workflow and exploratory analyses of feature-selection and dimensionality-reduction strategies. a) Reduction of VIF values; b) Trade-off between feature count and model predictive accuracy; c) PCA analysis of explained variance; d) Efficiency trade-off analysis.

First, the correlation analysis (Figure S18) confirmed the existence of widespread moderate-to-high linear correlations among the features. However, the subsequent stepwise elimination process using VIF (Figures S19a-b) revealed a non-monotonic relationship: as the number of features was reduced, the model's predictive accuracy (R^2^) did not consistently improve but instead exhibited complex fluctuations. This observation strongly suggests that simply removing statistically correlated features does not guarantee optimal model performance.

Furthermore, the PCA analysis (Figures S19c-d) showed that while dimensionality could be substantially reduced (60%-80%) with only a minor impact on predictive accuracy, it highlighted a trade-off between performance and dimensionality. The existence of this "efficiency zone" indicates that although redundancy exists among the features, the information they collectively carry is still necessary to maintain high predictive accuracy.

Taken together, these exploratory analyses point to a preliminary conclusion: the relationship between statistical correlation among features and model performance is not straightforward or linear. This provided the motivation and context for conducting the more rigorous statistical tests to determine if retaining all features was indeed the optimal strategy.

To ensure the conclusions are robust and independent of randomness from data partitioning, a rigorous statistical validation protocol was followed. Specifically, a 5$\times$10 nested cross-validation was employed: each strategy (the baseline and the three reduction methods) was evaluated using 10-fold cross-validation, and this process was repeated five times with different random seeds. This protocol generated a stable performance distribution of 50 independent R^2^ scores for each strategy, effectively mitigating biases that could arise from a single data partition. Finally, based on these distributions, a paired t-test was applied to compare the baseline model against each reduction strategy. The null hypothesis (H_0_) was that no significant performance difference exists, while the alternative hypothesis (H_1_) was that the retain all features baseline is significantly superior.

The results summarized in Figure S20 provide clear and statistically significant evidence for this claim. The performance distributions in Figure S20a show that the "retain all features" baseline achieves a higher average R^2^ score of 0.970, consistently outperforming all three feature-reduction strategies: Pearson (0.798), VIF (0.895), and PCA (0.928). Subsequently, the paired t-test results in Figure S20b confirm that this performance drop is statistically significant, as the p-values are well above the 0.05 threshold for all three strategies. This significance threshold aligns with established domain conventions, controlling the Type I error rate below 5% and achieving a balance between false positive control and statistical power.^[33]^ Consequently, the null hypothesis is rejected. The statistical evidence strongly supports the conclusion that for this complex materials science problem, eliminating statistically correlated features, even with sophisticated methods, leads to a significant degradation in model predictive accuracy.


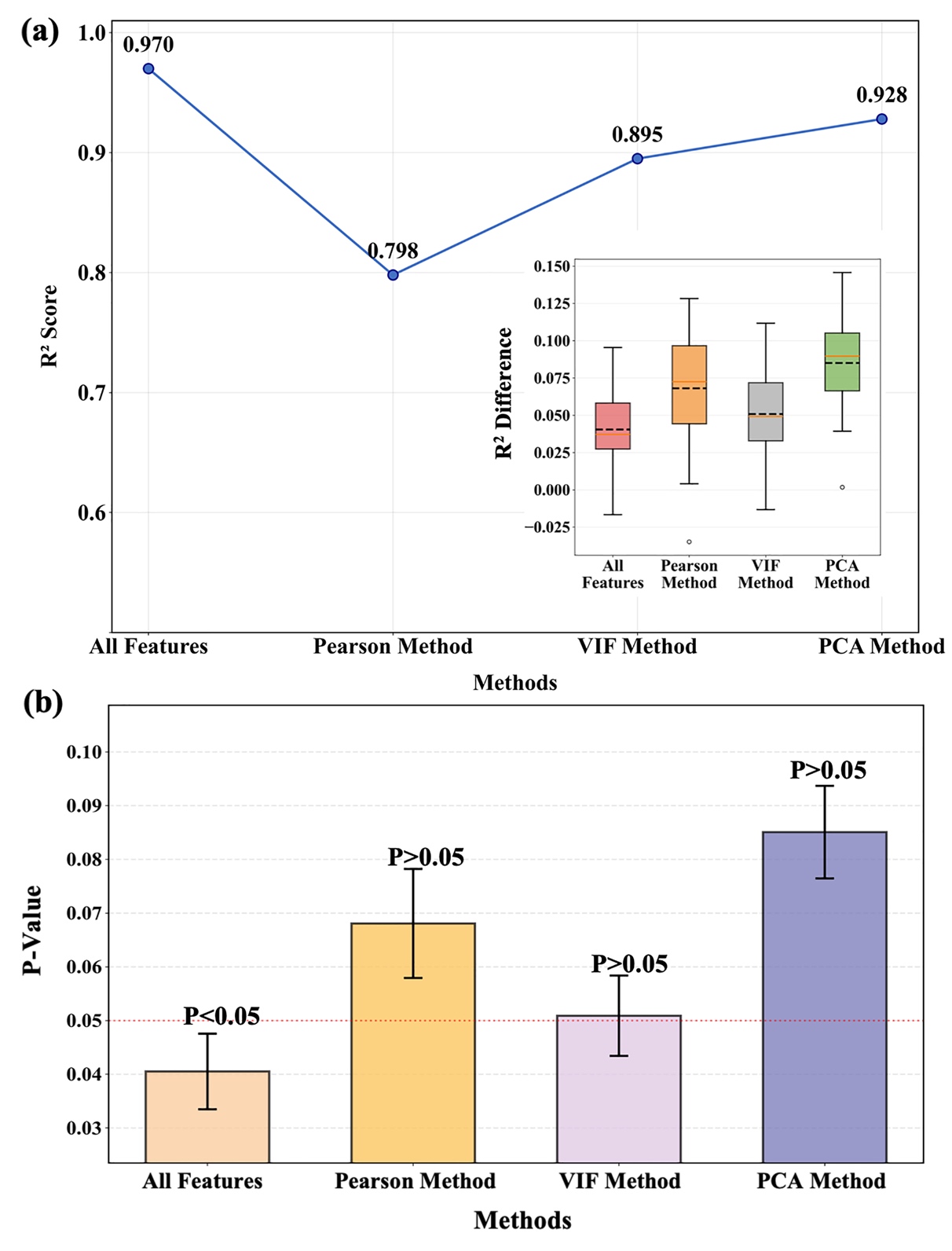


**Figure S20.** Rigorous statistical validation of the hypothesis that "eliminating correlated features reduces model accuracy." a) Distributions of average performance and performance differences across different strategies; b) Statistical significance results from the paired t-test.

This finding highlights a crucial insight for materials informatics: statistical correlation does not equate to informational redundancy. In complex, multicomponent systems like Ni-based superalloys, correlated features (e.g., Al and Ti contents) often encode unique and complementary physical information essential for capturing the underlying mechanisms. Retaining them enhances the model's ability to learn complex interactions, ultimately improving its stability and generalization capacity.

Regarding computational complexity, the framework's cost is primarily a trade-off for its higher accuracy. As shown in Figure S21, the TL+PLL method exhibits a moderate and predictable increase in computational cost compared to baselines. While more complex than simpler methods, this overhead is a necessary investment for its superior performance in handling ambiguous data and achieves a favorable balance between accuracy and efficiency.

The framework also demonstrates excellent scalability. The learning curves in Figure S28 confirm its ability to effectively leverage larger datasets, as model performance consistently improves with more data. Furthermore, its applicability extends to new alloy systems. As a proof-of-concept, the model achieved 87% accuracy in predicting the properties of high-entropy alloys (Figure S24), demonstrating its robust generalization potential.

Finally, a comprehensive hyperparameter sensitivity analysis was conducted to ensure the model's robustness, with results summarized in Figure S22.

For the base Random Forest model, the analysis of n_estimators and max_depth (Figures S22a-b) reveals that performance stabilizes around n_estimators = 200 and is optimal for a max_depth between 8 and 10. This indicates that high accuracy can be achieved without excessive model complexity or exhaustive tuning.

Regarding the PLL optimization process, the sensitivity analysis for the crucial error threshold (*e_₁_*) determined that a value of 3 provides the optimal balance between label accuracy and data coverage, with the detailed methodology presented in Supporting Information for Figure S13. Furthermore, the analysis of the GPR hyperparameters (Figures S22c-f) demonstrates clear performance peaks and stable behavior. For instance, the RBF kernel length scale achieves optimal performance around 1.0, while the noise level shows a distinct optimal point near 10^-5^. The existence of these well-defined optimal regions confirms that the model's success is robust and not dependent on fragile or overly specific hyperparameter choices.

In summary, this comprehensive analysis demonstrates that the TL+PLL framework is not only theoretically sound but also practically robust. Its moderate computational overhead is a justifiable trade-off for higher accuracy, it scales effectively, and its performance is not overly sensitive to hyperparameter choices.

**
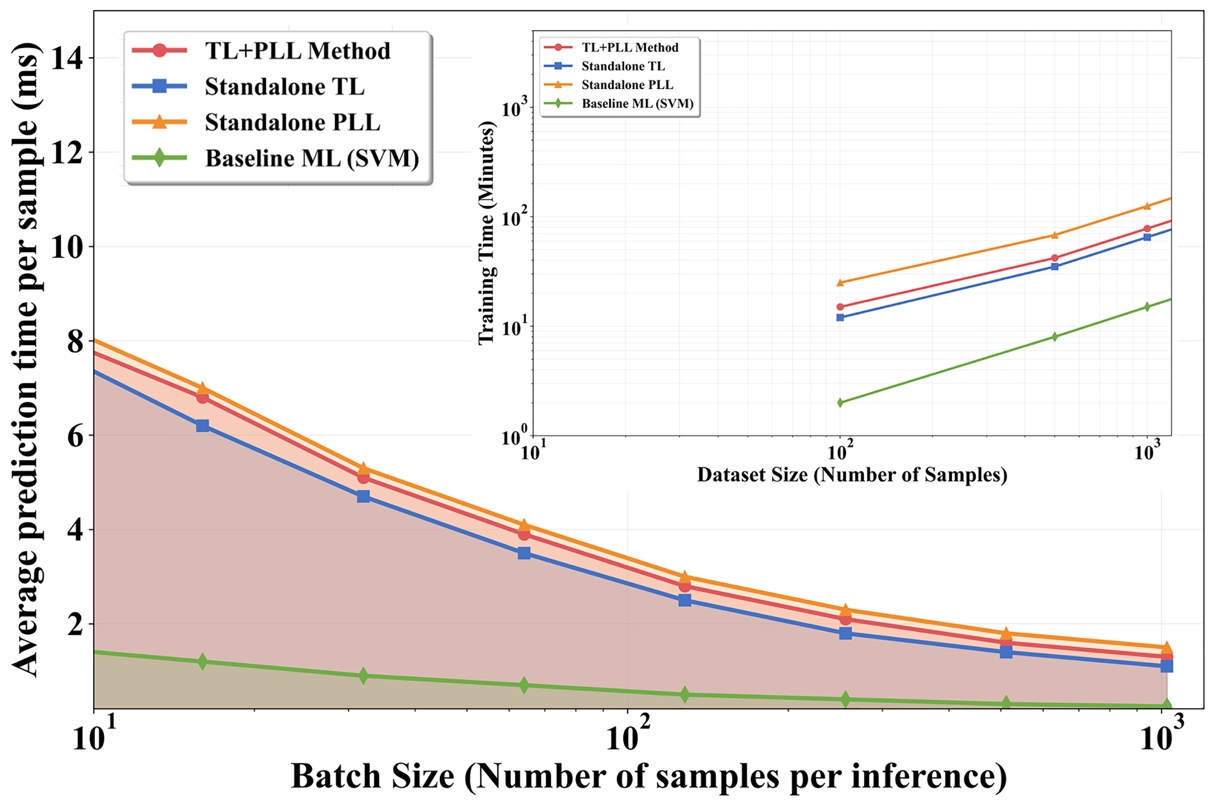
**

**Figure S21.** Computational cost and scalability analysis: comparison of the TL+PLL Method with Other Baselines.


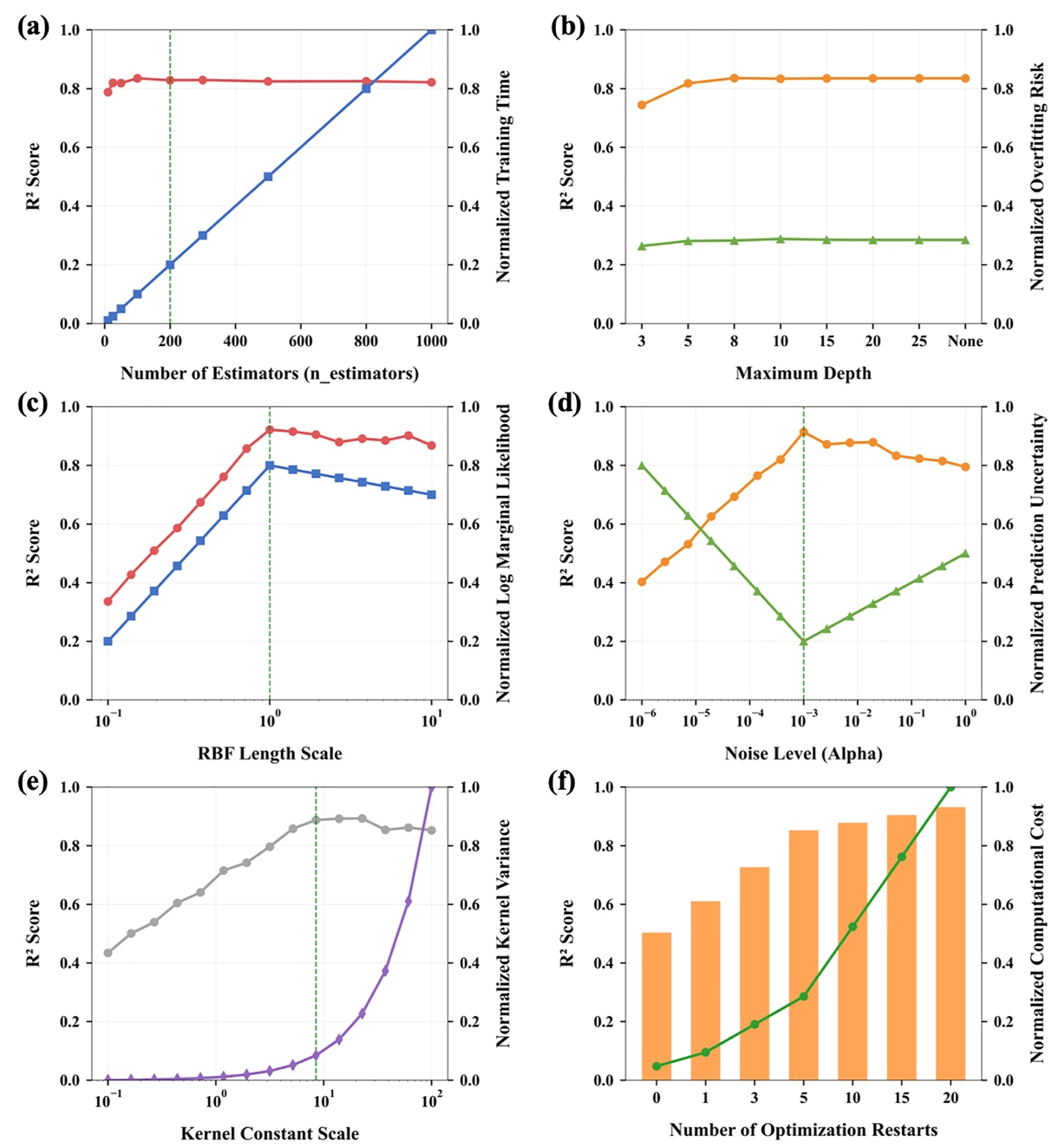


**Figure S22.** Systematic Sensitivity Analysis of Core Model Hyperparameters. a) Number of estimators (n_estimators); b) Maximum tree depth (max_depth); c) RBF kernel length scale; d) Noise level (α); e) Kernel constant scale; f) Number of optimization restarts.

**6. Details of Experimental validation**

The experimental materials are GH4169 high-temperature alloy rods, with their chemical compositions shown in Table S1. After hot rolling, the alloy was subjected to a standard heat treatment of 960 ℃ × 1 h air cooling + 720 ℃ × 8 h furnace cooling (cooling rate of 50 ℃-h-1) + 620 ℃ × 8 h air cooling to room temperature. The fatigue testing machine MTS-50kN-7 was used to first perform tensile tests and then low-cycle fatigue tests at room temperature (20°C). The tests were strain-controlled with a strain ratio of -1 and a strain rate of $6\times{10}^{-3}s^{-1}$. The total strain amplitudes during the experiment for GH4169 were 0.4%, 0.5% and 0.6%. The fatigue stress as well as the fatigue life in Table S5 are for the 0.6% total strain condition.

**Table S4** Chemical compositions of GH4169 high-temperature alloys (%, mass fraction).

| Alloy | Ni | Cr | Co | Fe | Al | Ti | Nb | Mo | W |
| --- | --- | --- | --- | --- | --- | --- | --- | --- | --- |
| INCONEL718 | 51.82 | 18.94 | 0.03 | 19.35 | 0.59 | 1.00 | 5.23 | 3.01 | 0 |

**Table S5** Tensile and Fatigue properties of GH4169 superalloy.

| UTS | YS | EL | RA | FS | FL |
| --- | --- | --- | --- | --- | --- |
| 1414 | 1181 | 22.5 | 42.2 | 982 | 2195 |

**
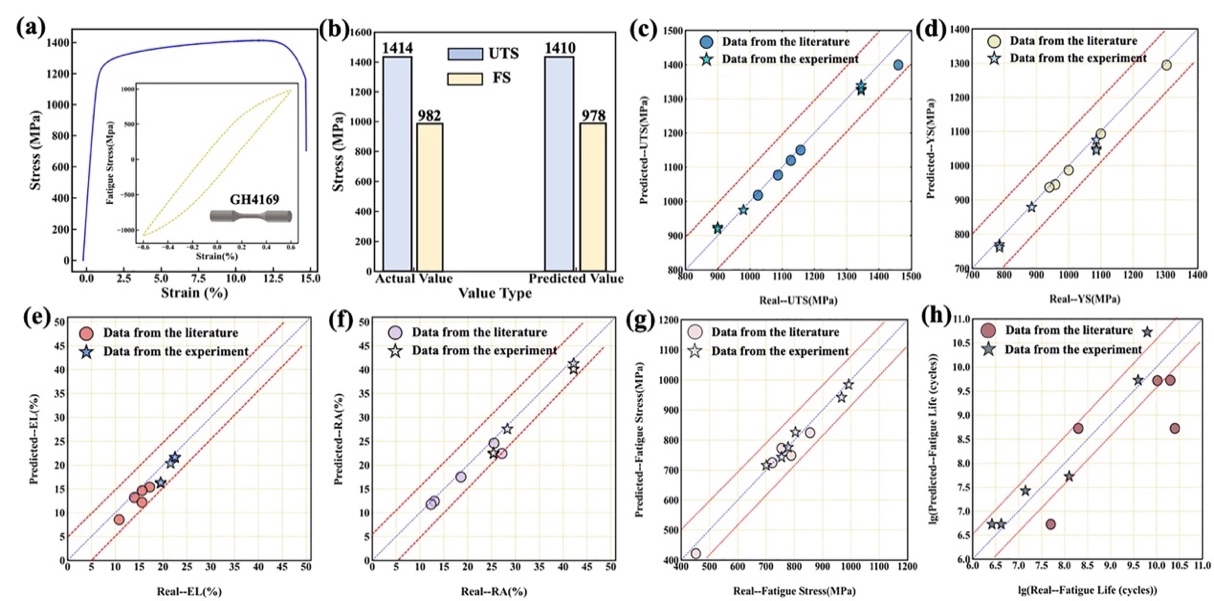
**

**Figure S23.** Experimental validation results of the model: a) Stress-strain curve of GH4169 alloy during tensile testing at room temperature and the hysteresis return line at half-life for fatigue testing of GH4169 alloy at room temperature with a total strain amplitude of 0.6%; b) Comparison of experimental values and model predictions; c)-f) Comparison of the model predictions and actual experimental values for the mechanical properties of 11 alloys: c) UTS; d) YS; e) EL; f) RA. g)-h) Comparison of the predicted values and actual experimental values for 11 alloys: g) Fatigue stress; h) Fatigue life.

**Table S6** Alloy compositions in 11 groups of Ni-based superalloys datasets.

|  | Alloy composition(wt.%) | | | | | | | | | |
| --- | --- | --- | --- | --- | --- | --- | --- | --- | --- | --- |
| Alloy | | Ni | Cr | Co | Fe | Al | Ti | Nb | Mo | W |
| AD730 | | 55.5 | 16 | 13 | 1 | 2.1 | 3.7 | 0.7 | 4 | 4 |
| GH4049 | | 58.1 | 10.25 | 15.0 | 0 | 4.05 | 1.65 | 0 | 5.0 | 0 |
| GH4720Li | | 51.9 | 15.83 | 14.75 | 18.9 | 2.64 | 4.95 | 0 | 2.93 | 1.24 |
| GH4169 | | 51.6 | 18.96 | 0 | 18.6 | 0.7 | 1.07 | 5.1 | 3.26 | 0 |
| GH4742 | | 63.17 | 14.00 | 10.03 | 0 | 2.63 | 2.67 | 2.48 | 5.02 | 0 |
| GH4169 | | 51.8 | 18.94 | 0.03 | 19.3 | 0.59 | 1 | 5.23 | 3.01 | 0 |
| GH4169 | | 51.8 | 18.94 | 0.03 | 19.3 | 0.59 | 1 | 5.23 | 3.01 | 0 |
| GH4169 | | 51.8 | 18.94 | 0.03 | 19.3 | 0.59 | 1 | 5.23 | 3.01 | 0 |
| GH4169G | | 52.2 | 19.2 | 0 | 18.7 | 0.5 | 1.1 | 5.2 | 3 | 0 |
| GH4169G | | 52.2 | 19.2 | 0 | 18.7 | 0.5 | 1.1 | 5.2 | 3 | 0 |
| GH4151 | | 55.5 | 15.1 | 11.6 | 0 | 3.8 | 3.0 | 3.7 | 4.6 | 2.7 |

**Table S7** Process and test parameters in 11 sets of Ni-based superalloys datasets.

| ST | STt | STat | Stat | AT | At | Δε*_t_* | Δε*_e_* | Δε*_p_* | T |
| --- | --- | --- | --- | --- | --- | --- | --- | --- | --- |
| 1080 | 4 | 0 | 0 | 760 | 8 | 0.413 | 0.408 | 0.005 | 750 |
| 1060 | 2 | 0 | 0 | 760 | 8 | 0.5 | 0.417 | 0.0083 | 500 |
| 1070 | 0 | 0 | 0 | 760 | 8 | 0.8 | 0.484 | 0.316 | 650 |
| 960 | 1 | 720 | 8 | 620 | 8 | 0.6 | 0.0443 | 0.0057 | 550 |
| 1115 | 0 | 845 | 4 | 720 | 8 | 0.6 | 0.31 | 0.28 | 650 |
| 960 | 1 | 720 | 8 | 620 | 8 | 0.4000 | 0.3675900 | 0.032411 | 20 |
| 960 | 1 | 720 | 8 | 620 | 8 | 0.5000 | 0.4724300 | 0.027571 | 20 |
| 960 | 1 | 720 | 8 | 620 | 8 | 0.6000 | 0.4480900 | 0.1519 | 20 |
| 975 | 1 | 720 | 8 | 620 | 8 | 1.7 | 1.65322 | 0.55 | 450 |
| 975 | 1 | 720 | 8 | 620 | 8 | 2.1 | 2.0115 | 0.046 | 450 |
| 1080 | 4 | 840 | 6 | 720 | 8 | 0.5 | 0.465 | 0.035 | 600 |

**Table S8** Microstructural features in 11 sets of Ni-based superalloys datasets.

| V*_p_* | T*_p_* | E | G | SFE |
| --- | --- | --- | --- | --- |
| 35.51 | 1110 | 172 | 64.46 | 120.7 |
| 37.11 | 1211.54 | 167.24 | 72.54 | 112.1 |
| 37.06 | 1109.54 | 162.24 | 60.9 | 122 |
| 24.61 | 935.78 | 224.54 | 60.99 | 25.1 |
| 34.61 | 1100.78 | 182.54 | 60.99 | 125.1 |
| 23.62 | 922.65 | 203.24 | 75.66 | 22 |
| 23.62 | 922.65 | 203.24 | 75.66 | 22 |
| 23.62 | 922.65 | 203.24 | 75.66 | 22 |
| 24.62 | 925.65 | 207.54 | 77.66 | 24 |
| 24.62 | 925.65 | 207.54 | 77.66 | 24 |
| 39.1 | 1140.66 | 147.23 | 60.43 | 107 |

**Table S9** Mechanical properties of 11 groups of Ni-based superalloys s in the dataset.

| UTS | YS | EL | RA | FS | FL |
| --- | --- | --- | --- | --- | --- |
| 1127 | 1000 | 14 | 13 | 722 | 22509 |
| 1460 | 1304 | 17.2 | 27.16 | 789 | 29966 |
| 1157 | 1100 | 10.8 | 25.5 | 856 | 4038 |
| 980 | 885 | 21.6 | 28.3 | 755 | 34522 |
| 1025 | 958 | 15.6 | 12.3 | 854 | 2294 |
| 1345 | 1086 | 22.5 | 42.2 | 701 | 13648 |
| 1345 | 1086 | 22.5 | 42.2 | 778 | 3469 |
| 1345 | 1086 | 22.5 | 42.2 | 804 | 2195 |
| 900 | 785 | 19.5 | 25.4 | 967 | 1286 |
| 900 | 785 | 19.5 | 25.4 | 992 | 620 |
| 1087 | 940 | 15.63 | 18.57 | 452 | 1780 |

**
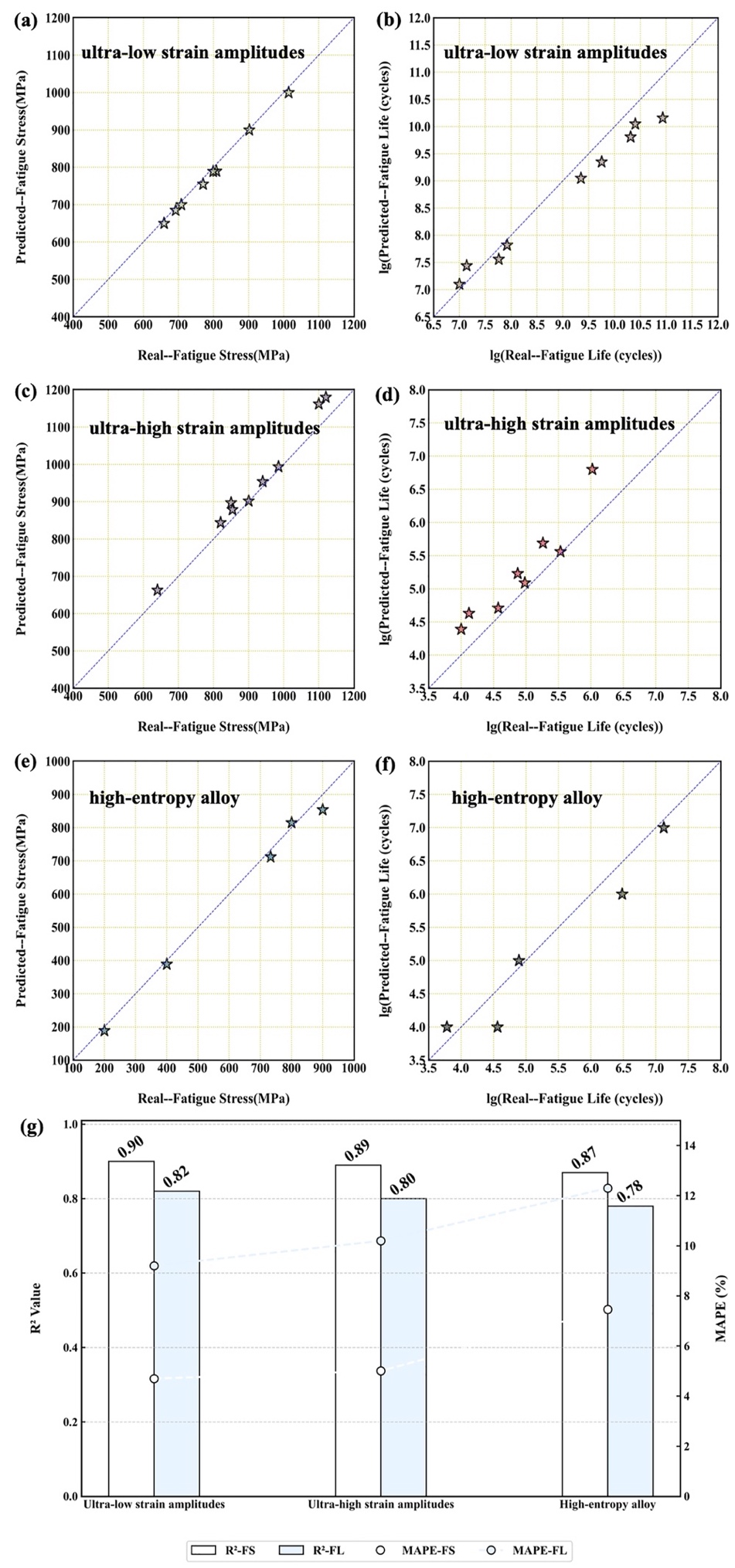
**

**Figure S24.** Experimental validation results of the model: a-f) Scatter plots comparing experimental values with model predictions; g) Comparison of R² and MAPE values between experimental results and model predictions.


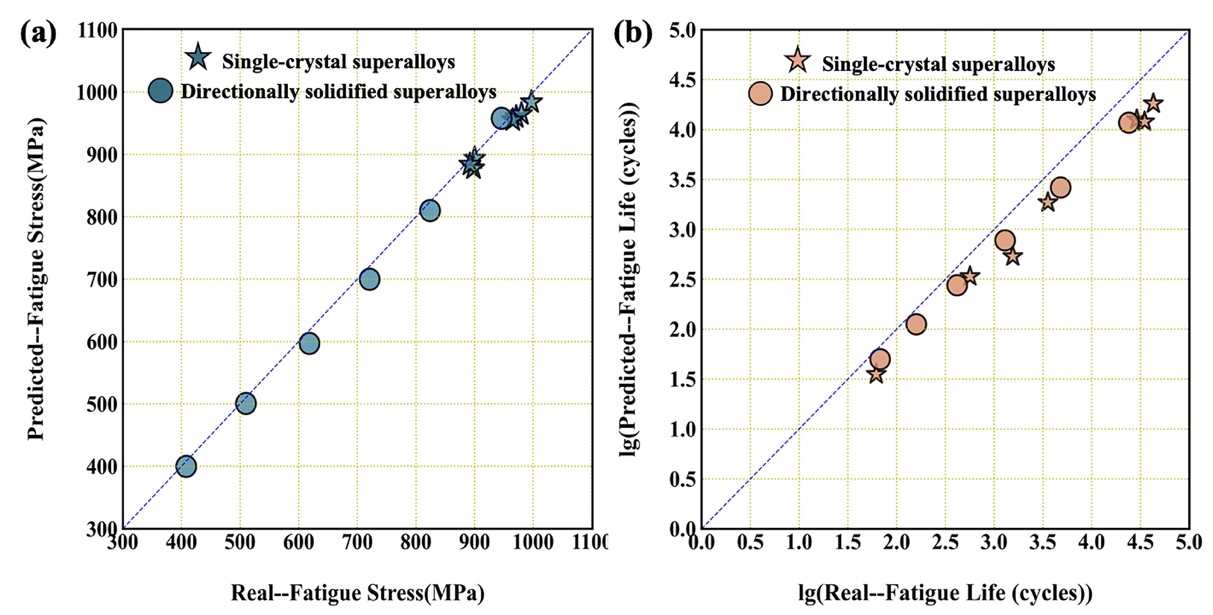


**Figure S25.** Model validation results: a-b) Scatter plots comparing experimental values with model predictions for single-crystal and directionally solidified superalloys; a) Fatigue strength prediction; b) Fatigue life prediction.

**Table S10.** Alloy compositions in 9 groups of Ni-based superalloys datasets.

| Ni | Cr | Co | Fe | Al | Ti | Nb | Mo | W |
| --- | --- | --- | --- | --- | --- | --- | --- | --- |
| 52.8 | 16.05 | 18.91 | 0.02 | 1.78 | 3.255 | 0.557 | 4.044 | 1.589 |
| 52.9 | 15.93 | 19.37 | 2.01 | 1.657 | 3.255 | 0.538 | 4.076 | 1.59 |
| 53.5 | 15.93 | 18.67 | 1.88 | 1.575 | 3.195 | 0.501 | 4.18 | 1.546 |
| 53.2 | 15.93 | 19.37 | 2.39 | 1.654 | 3.255 | 0.538 | 4.212 | 1.59 |
| 52.8 | 15.94 | 19.47 | 0.02 | 1.776 | 3.255 | 0.54 | 4.019 | 1.577 |
| 66.7 | 17.80 | 4.96 | 1.36 | 1.934 | 3.709 | 1.604 | 4.245 | 0.736 |
| 63.2 | 17.80 | 5.59 | 1.32 | 2.364 | 3.833 | 1.659 | 4.229 | 0.974 |
| 74.2 | 17.91 | 4.37 | 1.42 | 1.4 | 3.706 | 1.433 | 3.409 | 0.71 |
| 73.1 | 18.76 | 3.75 | 2.26 | 1.38 | 3.702 | 2.085 | 5.618 | 0.868 |
| 52.86 | 16.05 | 18.91 | 0.02 | 1.78 | 3.255 | 0.557 | 4.044 | 1.589 |
| 52.92 | 15.93 | 19.37 | 2.01 | 1.657 | 3.255 | 0.538 | 4.076 | 1.59 |

**Table S11.** Alloy compositions in 11 groups of Ni-based superalloys datasets.

| Ni | Cr | Co | Fe | Al | Ti | Nb | Mo | W |
| --- | --- | --- | --- | --- | --- | --- | --- | --- |
| 62.61 | 22.05 | 5.139 | 0.50 | 1.851 | 3.109 | 2.241 | 4.526 | 1.883 |
| 55.79 | 20.86 | 5.429 | 2.57 | 2.267 | 3.328 | 1.612 | 4.429 | 1.505 |
| 54.31 | 18.20 | 3.152 | 15.6 | 0.515 | 0.838 | 5.228 | 2.844 | 1.008 |
| 53.85 | 18.26 | 0.043 | 19.6 | 0.51 | 0.973 | 5.004 | 3.409 | 0.286 |
| 65.81 | 17.97 | 6.675 | 0.49 | 1.799 | 2.608 | 2.172 | 4.961 | 0.438 |
| 63.16 | 17.90 | 7.525 | 0.27 | 2.272 | 3.573 | 2.195 | 4.949 | 0.652 |
| 60.81 | 18.75 | 6.644 | 2.51 | 1.901 | 1.378 | 0.008 | 5.275 | 6.034 |
| 73.79 | 17.00 | 3.925 | 0.32 | 3.03 | 3.808 | 2.35 | 6.218 | 1.338 |
| 71.68 | 17.73 | 2.754 | 3.76 | 2.586 | 3.794 | 2.048 | 3.948 | 1.323 |
| 62.61 | 22.05 | 5.139 | 0.50 | 1.851 | 3.109 | 2.241 | 4.526 | 1.883 |
| 55.79 | 20.86 | 5.429 | 2.57 | 2.267 | 3.328 | 1.612 | 4.429 | 1.505 |

**Table S12.** Alloy compositions in 1 groups of High-entropy alloys datasets.

| Ni | Cr | Co | Fe | Al | ST | STt | Δε*_t_* | Δε*_e_* | Δε*_p_* |
| --- | --- | --- | --- | --- | --- | --- | --- | --- | --- |
| 24.56 | 21.76 | 24.66 | 23.3 | 5.64 | 1100 | 1 | 3.5 | 0.9 | 2.6 |
| 24.56 | 21.76 | 24.66 | 23.3 | 5.64 | 1100 | 1 | 2.5 | 0.9 | 1.6 |
| 24.56 | 21.76 | 24.66 | 23.3 | 5.64 | 1100 | 1 | 2 | 0.9 | 1.1 |
| 24.56 | 21.76 | 24.66 | 23.3 | 5.64 | 1100 | 1 | 1 | 0.9 | 0.1 |
| 24.56 | 21.76 | 24.66 | 23.3 | 5.64 | 1100 | 1 | 0.5 | 0.9 | -0.4 |

**Table S13.** Alloy compositions in 1 groups of Ni-based single-crystal superalloy dataset.

| Ni | Cr | Co | Ta | Al | Re | Nb | Mo | W | Hf | Ti |
| --- | --- | --- | --- | --- | --- | --- | --- | --- | --- | --- |
| 55.65 | 4.8 | 9.5 | 8.5 | 6.2 | 2.4 | 1.2 | 2.5 | 9.0 | 0.15 | 0.1 |

**Table S14.** Alloy compositions in 1 groups of Directionally solidified superalloy dataset.

| C | Cr | W | Co | Mo | Al | Ti | B | Ni |
| --- | --- | --- | --- | --- | --- | --- | --- | --- |
| 0.7 | 9.5 | 4.8 | 4.5 | 3.8 | 5.2 | 2.3 | 0.015 | 69.18 |


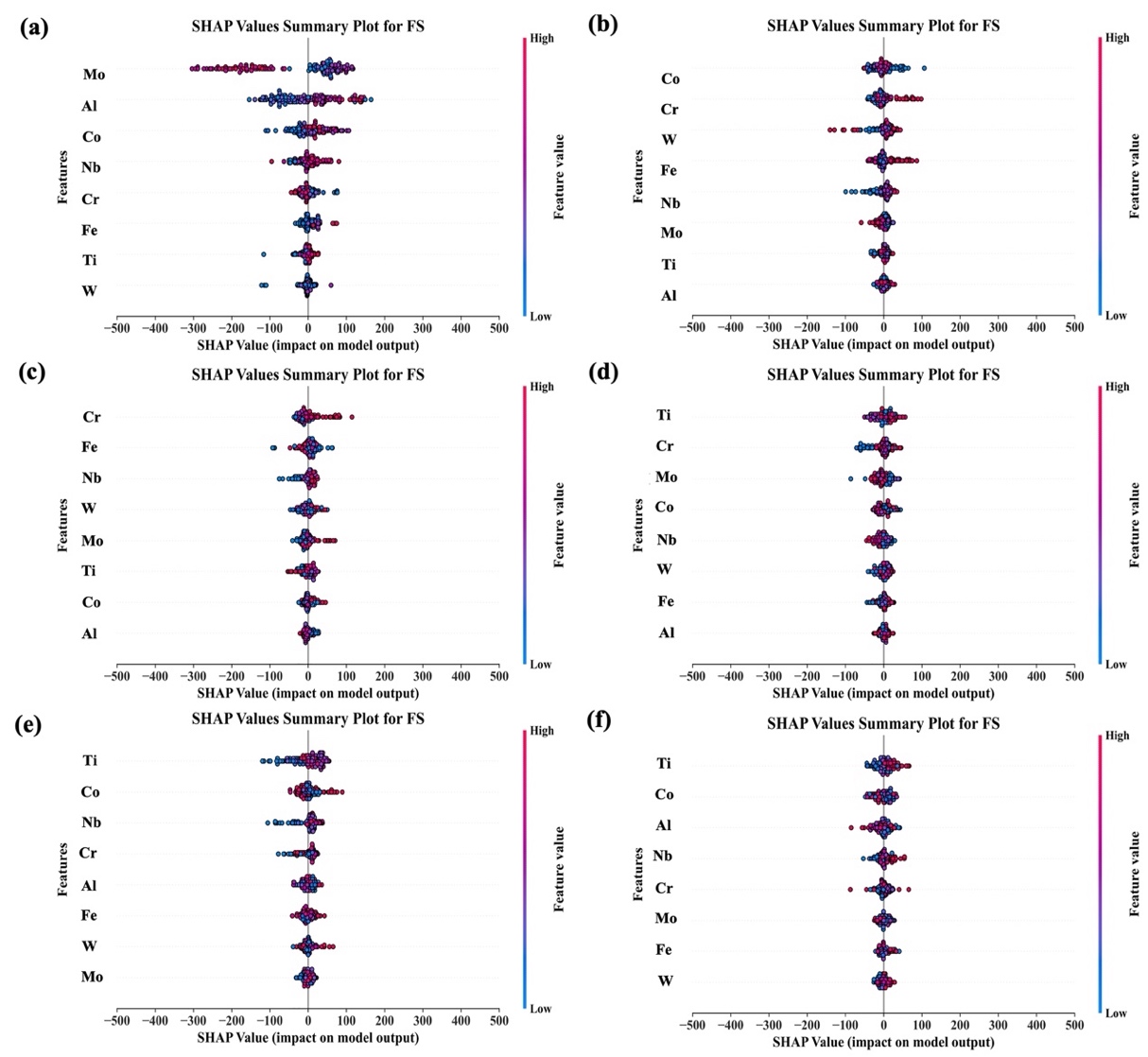


**Figure S26.** a)-f) Display SHAP summary plots for optimizations one through six, respectively, with SHAP values (magnitude of effect on model output) on the horizontal axis and major alloy compositions on the vertical axis. The color gradient from blue to red indicates the trend of the composition taking values from low to high. In each optimization, larger SHAP values indicate a more significant contribution of that alloy composition to performance. As the optimization progresses, the evolving influence of different compositions on performance can be observed.


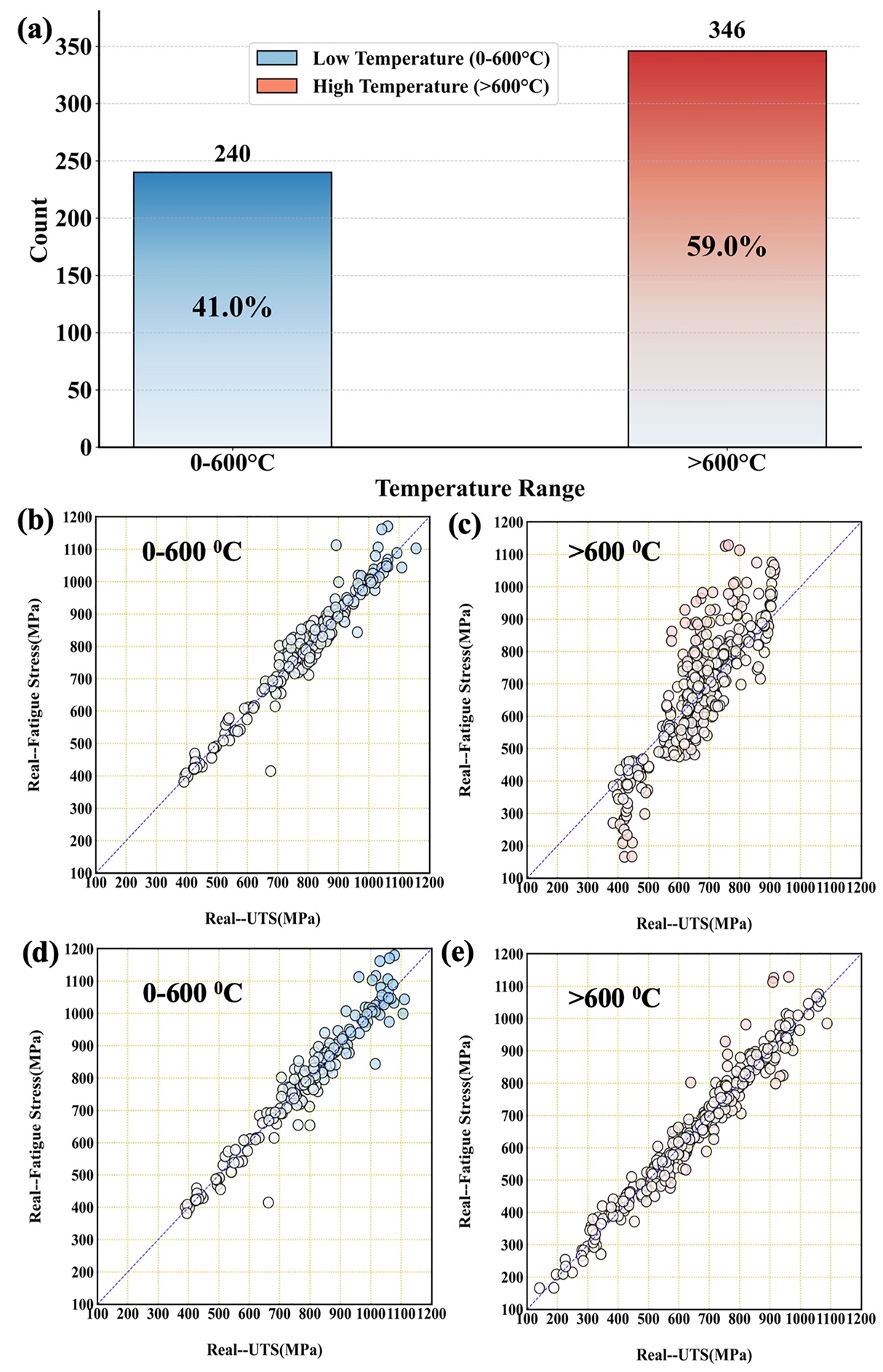


**Figure S27.** Illustrates the prediction results of fatigue data using Eqs. (3) and (5) from the manuscript. a) Depicts the data distribution of low- and high-temperature samples in the fatigue database; b) Presents a scatter plot of the predictions from Eq. (3) for low-temperature data; c) Shows a scatter plot of the predictions from Eq. (3) for high-temperature data; d) Displays a scatter plot of the predictions from Eq. (5) for low-temperature data; e) Provides a scatter plot of the predictions from Eq. (5) for high-temperature data.

**7. Details of Model validation**

To provide a more intuitive visualization of the model training dynamics and its generalization capabilities, we have included a “learning curve” analysis in the supplementary materials. By plotting the trend of model performance on the training set and validation set as the number of samples changes, the learning curve clearly reveals whether the model exhibits high bias (underfitting) or high variance (overfitting) issues. This method has been widely adopted in materials science, such as in the fatigue life prediction of Ni-based superalloys, to assess the generalization capability of the model.^[34]^

Using data from Ni-based superalloys under the TL + PLL strategy, we generated learning curves for fatigue stress and fatigue life, as depicted in Figure S28. Figures S28a-b illustrate that, with an increasing number of training samples, the R² values for the training and validation sets rise from approximately 0.6 and 0.2 to 0.97 and 0.95, respectively, indicating effective learning of data patterns. The training set R² (0.97) slightly exceeds the validation set R² (0.95), with a difference of 0.02, which is within an acceptable range and suggests minimal overfitting. The validation set MAPE increases slightly for sample sizes between 300 and 400, potentially due to local overfitting or outliers, such as the high variability of Co observed in Supplementary Figure S2. Therefore, future work will consider adopting more robust algorithms or outlier detection to address potential outliers, further enhancing the stability of the model. For sample sizes below 100, both training and validation R² values remain low (R^2^<0.2), indicating underfitting and the need for additional samples to capture the nonlinear relationships. Figures S28c-d demonstrate that, as sample size increases, the training set R² stabilizes between 0.8 and 1.0, with a MAPE of 5%, reflecting a robust fit to the training data. The validation set R² improves from 0.2 to 0.87, and the MAPE decreases from 27% to 5%, indicating enhanced generalization and reduced prediction error. However, for sample sizes below 200, the training set R² significantly exceeds the validation set R² (0.9 vs. 0.2, a difference of 0.7), suggesting pronounced overfitting. A negative validation set R² indicates that predictions deviate substantially from actual values, likely due to overfitting caused by noise in the training data and the complexity of the fatigue life variable post-log transformation.


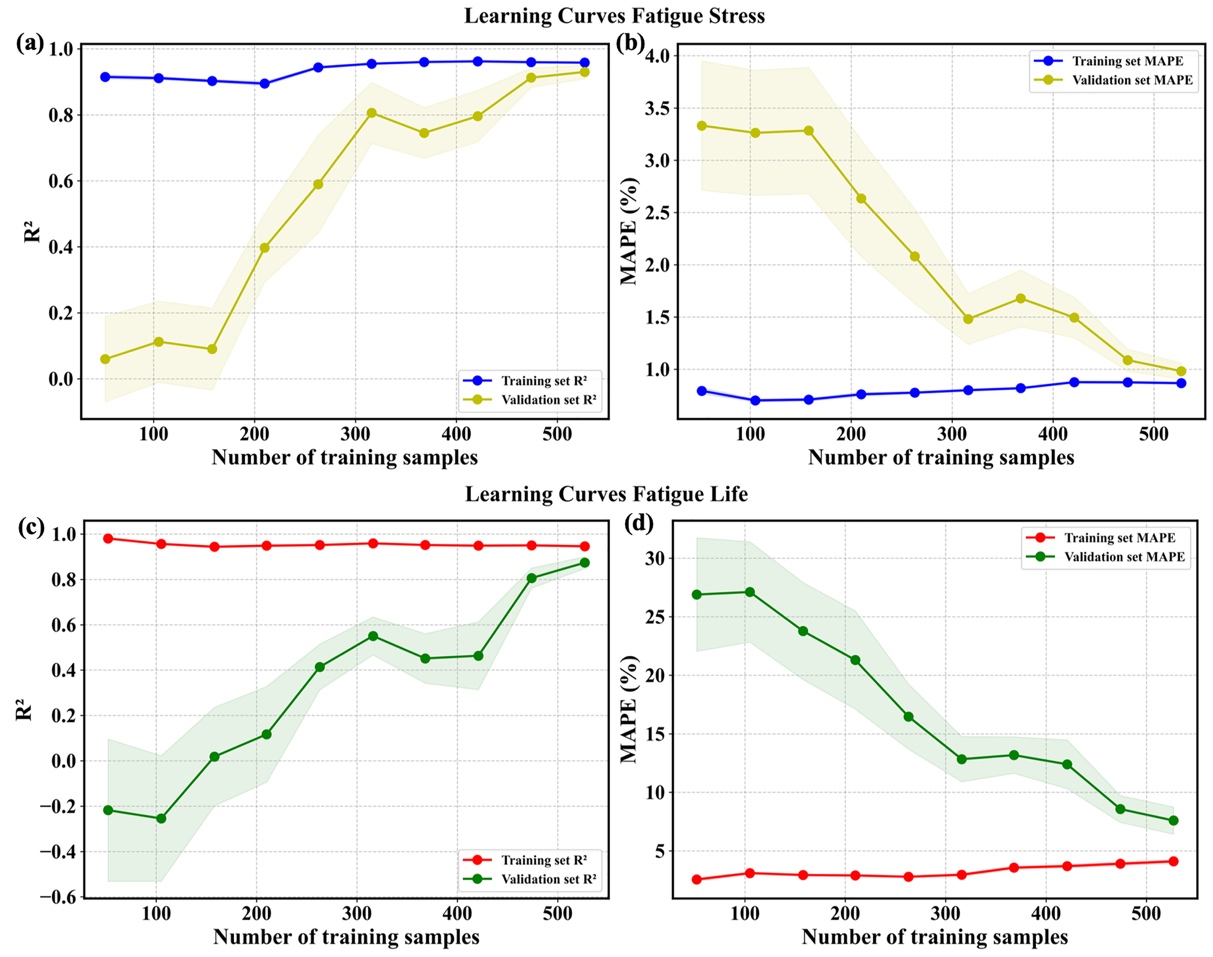


**Figure S28.** a-b) Learning curves for the fatigue stress prediction model, illustrating the relationships between R², MAPE, and the number of training samples, with the solid blue line indicating training set performance and the shaded yellow region indicating validation set performance. c-d) Learning curves for the fatigue life prediction model, depicting the trends in R² and MAPE as the number of training samples increases, with the solid green line indicating training set performance and the shaded green region indicating validation set performance.

To elucidate the predictive mechanisms of the model, we employed partial dependence plot (PDP) analysis to examine the influence of key features on model outputs. PDP visualizes the average effect of one or more features on the target variable, revealing nonlinear or interactive effects captured by the model. Unlike SHAP, which quantifies individual feature contributions, PDP provides global average effects, enhancing insight into the predictive logic of the model.^[35]^ Figure S29 presents the PDP analysis results for key features. The results reveal a pronounced negative correlation between Δε*_t_* and fatigue life, with distinct nonlinear behavior: as Δε*_t_* increases from 0 to 0.1, fatigue life remains stable at 9.2; beyond 0.1, it declines rapidly, reaching 5.5 at Δε*_t_* > 0.2. This trend aligns with the Coffin-Manson relationship, reflecting a negative power-law dependence on strain amplitude. Similarly, Δε*_p_* exhibits a negative correlation with fatigue life, though its effect is less pronounced than that of Δε*_t_*. As Δε*_p_* increases from 0 to 0.05, fatigue life decreases from 8.5 to 7.3, stabilizing thereafter, indicating a primary influence at lower values. Conversely, as Volume fraction of the precipitate phase (V*_p_*) increases from 0 to 0.5, fatigue life rises from 7.3 to 7.8, then declines as V*_p_* approaches 0.5. UTS and YS show a positive correlation with fatigue life, whereas EL exhibits minimal influence, with no clear trend.


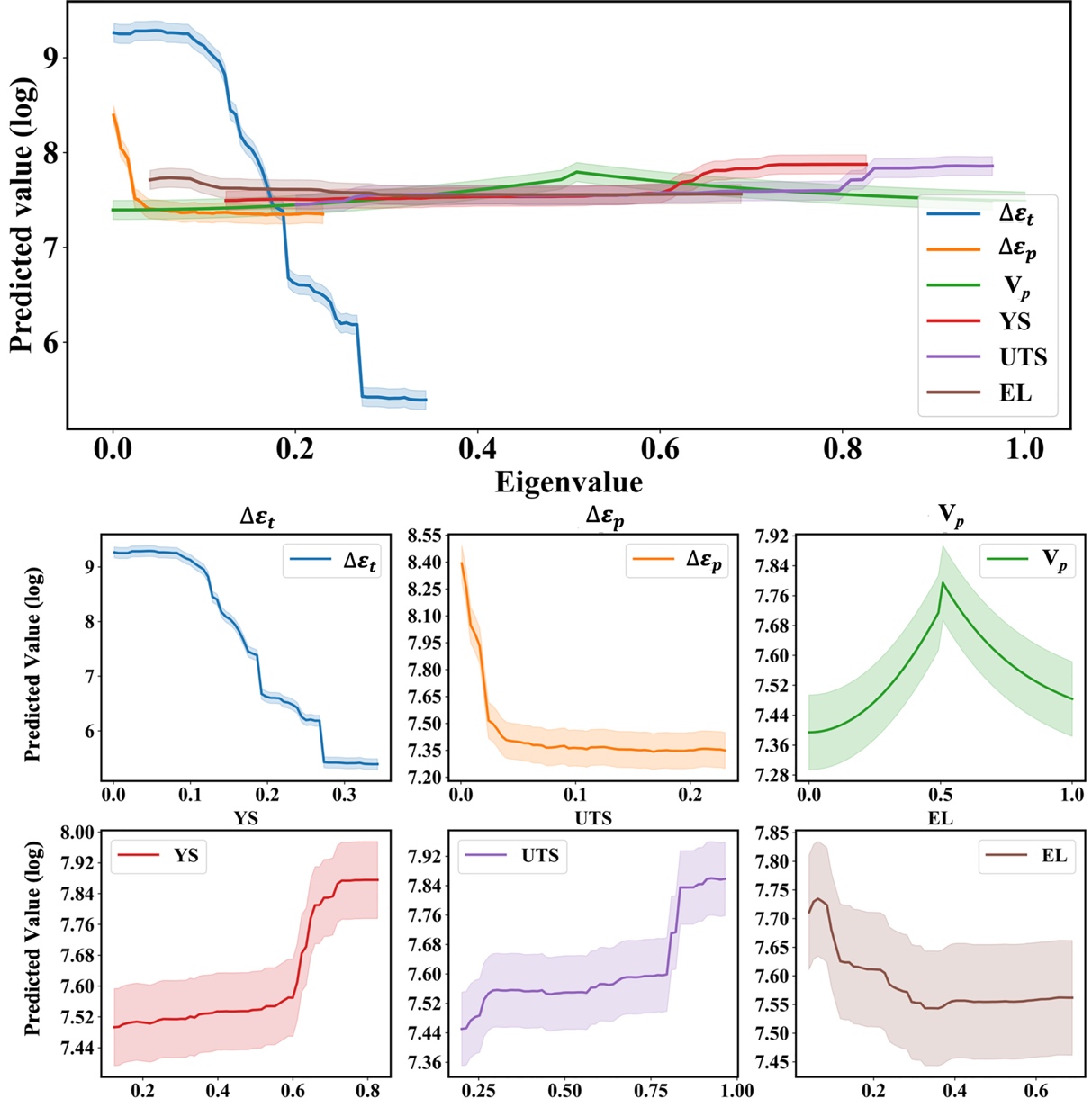


**Figure S29.** Partial dependence plots illustrating the relationships between the predicted fatigue life of Ni-based superalloys and key variables, with each curve depicting the influence of the variables Δε*_t_*, Δε*_p_*, V*_p_*, YS, UTS, and EL on the model output, highlighting their relative contributions across different ranges.


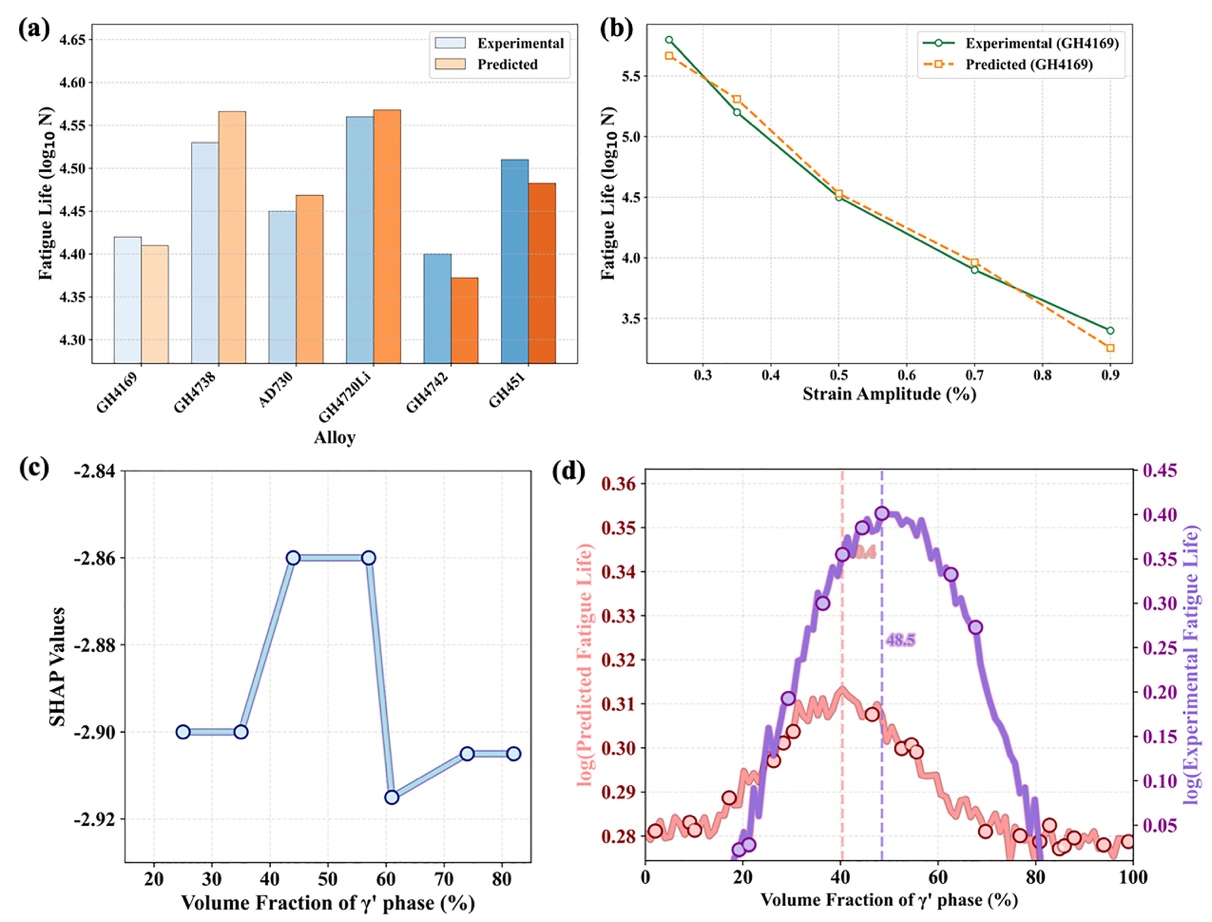


**Figure S30.** Illustrates the relationship between SHAP analysis and experimental results: a) Fatigue life predictions for different alloys under identical test conditions; b) Fatigue life predictions for the GH4169 alloy under varying test conditions.

**8. Data collection**

In this study, tensile data for Ni-based deformation superalloys were collected and extracted from reliable public literature and patents to train the ML model. The feature space formed by the data is shown in Table S15. Table 1 presents 1020 sets of data on the tensile properties of Ni-based superalloys. Each dataset includes 16 input features, encompassing chemical compositions, heat treatment process parameters and test parameters. In addition, four target features, namely tensile strength (UTS), yield strength (YS), elongation (EL) and reduction of area (RA) are included. The database is primarily used as a mediator for fatigue life model development, especially for tensile performance prediction and feature-aware disambiguation methods. The database is named Tensile Database. This dataset represents a valuable resource for TL. TL utilizes related techniques to apply methods developed for predicting tensile composition models to the prediction of fatigue compositions.^[36]^

**Table S15** Database of tensile properties of Ni-based superalloys

| Type | Variable | Symbol | Minimum | Maximum |
| --- | --- | --- | --- | --- |
| Inputs | Ni (wt.%) | Ni | 46.707 | 75.715 |
|  | Chromium (wt.%) | Cr | 12.690 | 33.810 |
|  | Cobalt (wt.%) | Co | 0.000 | 25.000 |
|  | Iron (wt.%) | Fe | 0.000 | 20.477 |
|  | Aluminum (wt.%) | Al | 0.400 | 3.700 |
|  | Titanium (wt.%) | Ti | 0.047 | 5.040 |
|  | Niobium (wt.%) | Nb | 0.000 | 5.500 |
|  | Molybdenum (wt.%) | Mo | 0.000 | 9.830 |
|  | Tungsten (wt.%) | W | 0.000 | 10.410 |
|  | Temperature (^o^C) | T | 19.00 | 1200.00 |
|  | Solid solution temperature (^o^C) | ST | 0.00 | 1140.00 |
|  | Solid solution time (h) | STt | 0.00 | 48.00 |
|  | Stabilizing aging temperature (^o^C) | STat | 0.00 | 1150.00 |
|  | Stabilization aging time (h) | Stat | 0.00 | 75.00 |
|  | Aging temperature (^o^C) | AT | 0.00 | 950.00 |
|  | Aging time (h) | At | 0.00 | 36.00 |
| Outputs | Tensile strength (MPa) | UTS | 20.00 | 1834.00 |
|  | Yield strength (MPa) | YS | 41.00 | 1477.00 |
|  | Elongation (%) | EL | 2.50 | 136.00 |
|  | Reduction of area (%) | RA | 10.79 | 76.55 |

Table S16 presents 622 sets of low-cycle fatigue test data extracted from the CHINA SUPERALLOYS HANDBOOK, primarily focusing on Ni-based superalloys. Each dataset contains test parameters, heat treatment process parameters and two key target characteristics: fatigue stress and fatigue life, referred to as the Fatigue Database. However, since the fatigue chemical compositions in this dataset are given as ranges rather than specific values, the main challenge lies in determining the specific values of the compositions to enrich the data quality and improve the fatigue performance prediction models. In the field of ML, this approach is known as PLL.^[37]^ The goal of PLL is to learn how to extract the correct labels from ambiguous training data and eliminate the ambiguity of the true expression within the candidate label set.

**Table S16** Fatigue life database for Ni-based superalloys

| Type | Variable | Symbol | Minimum | Maximum |
| --- | --- | --- | --- | --- |
| Inputs | Solid solution temperature (^o^C) | ST | 0 | 1260 |
|  | Solid solution time (h) | STt | 0 | 8 |
|  | Stabilizing aging temperature (^o^C) | STat | 0 | 1120 |
|  | Stabilization aging time (h) | Stat | 0 | 24 |
|  | Aging temperature (^o^C) | AT | 0 | 980 |
|  | Aging time (h) | At | 0 | 32 |
|  | Total strain range (%) | Δε*_t_* | 0.00247 | 4.065 |
|  | Elastic strain range (%) | Δε*_e_* | 0.00224 | 1.802 |
|  | Plastic strain range (%) | Δε*_p_* | -0.00037 | 3.375 |
|  | Temperature (^o^C) | T | 20 | 1000 |
| Outputs | Fatigue stress (MPa) | FS | 166 | 1203 |
|  | Fatigue life (Cycles) | FL | 62 | 275518 |

In Ni-based superalloys, there is a strong correlation between microstructure features and fatigue properties.^[38]^ Considering the significance and computational feasibility of these features, the equilibrium phase composition and physical properties at the test temperature were computed in batches from the TCNi-12 database using Thermo-Calc software. Five microstructural features were chosen, encompassing the volume fraction of the precipitate phase, the melting temperature of the precipitate phase, Young's modulus, shear modulus and stacking fault energy. Table S17 presents the feature space of the calculated microstructural features. The quantity and distribution of V*_p_* are intimately linked to the mechanical properties of the alloy.^[39]^ It influences fatigue crack propagation behavior by altering slip characteristics at the crack tip; a higher V*_p_* content improves the reversibility of dislocation slip, reduces the dislocation accumulation and enhances fatigue life.^[40]^ SFE plays a pivotal role in metal plastic deformation and damage processes; its primary function is to regulate the mechanical properties of metals by modifying slip modes and twinning mechanisms.^[41]^

**Table S17** The feature space of the microstructural features after determining the fatigue compositions.

| Features | Symbol | | Minimum | Maximum |
| --- | --- | --- | --- | --- |
| Volume fraction of the precipitate phase | | V*_p_* | 15.70 | 40.51 |
| Solvus temperature of the precipitate phase | | T*_p_* | 935.24 | 1107.70 |
| Young's modulus | | E | 159.05 | 175.71 |
| Shear modulus | | G | 58.82 | 65.83 |
| Stacking fault energy | | SFE | 137.04 | 262.22 |

**References**

[1] Jiang, L., Wang, C., Fu, H., Shen, J., Zhang, Z., Xie, J., Discovery of aluminum alloys with ultra-strength and high-toughness via a property-oriented design strategy, 2022, J. Mater. Sci. Technol., 98, 33-43, <https://doi.org/10.1016/j.jmst.2021.05.011>.

[2] Hu, M., Tan, Q., Knibbe, R., et al., Prediction of Mechanical Properties of Wrought Aluminium Alloys Using Feature Engineering Assisted Machine Learning Approach, 2021, Metall. Mater. Trans. A, 52, 2873-2884, <https://doi.org/10.1007/s11661-021-06279-5>.

[3] Reed, R.C. 2006, The Superalloys: Fundamentals and Applications, Cambridge University Press (Cambridge), ISBN: 9780511541285.

[4] Dong, H., Chen, Y., Wu, D., Ma, H., Feng, Z., Su, R., Unlocking the potential of trace cobalt in Ni-based superalloys: A molecular dynamics study on dislocation behavior and high-temperature stability, 2025, Mater. Des., 253, 113905, <https://doi.org/10.1016/j.matdes.2025.113905>.

[5] Su, R., Hao, D., He, P., Wu, D., Wang, Q., Dong, H., Ma, H., Effect of Co on creep and stress rupture properties of nickel-based superalloys – A review, 2023, J. Alloys Compd., 967, 171744, <https://doi.org/10.1016/j.jallcom.2023.171744>.

[6] Pollock, T.M., Tin, S., Nickel-based superalloys for advanced turbine engines: chemistry, microstructure and properties, 2006, J. Propul. Power, 22, 361-374, <https://doi.org/10.2514/1.18239>.

[7] Yang, S., Yang, L., Wang, Y., Determining the fatigue parameters in total strain life equation of a material based on monotonic tensile mechanical properties, 2020, Eng. Fract. Mech., 226, 106866, <https://doi.org/10.1016/j.engfracmech.2019.106866>.

[8] Zhao, E., Zhou, Q., Qu, W., Wang, W., Fatigue Properties Estimation and Life Prediction for Steels under Axial, Torsional, and In-Phase Loading, 2020, Adv. Mater. Sci. Eng., 2020, 8186159, <https://doi.org/10.1155/2020/8186159>.

[9] Shannon, C.E., A mathematical theory of communication, 1948, The Bell system technical journal, 27, 379-423.

[10] Segev, N., Harel, M., Mannor, S., Crammer, K., El-Yaniv, R., Learn on Source, Refine on Target: A Model Transfer Learning Framework with Random Forests, 2017, IEEE Trans. Pattern Anal. Mach. Intell., 39, 1811-1824, <https://doi.org/10.1109/TPAMI.2016.2618118>.

[11] Quinlan, J.R., Induction of decision trees, 1986, Mach. Learn., 1, 81-106, <https://doi.org/10.1007/BF00116251>.

[12] Pedregosa, F., Varoquaux, G., Gramfort, A., et al., Scikit-learn: Machine learning in Python, 2011, J. Mach. Learn. Res., 12, 2825-2830, <http://scikit-learn.sourceforge.net>.

[13] Aler, R., Valls, J.M., Boström, H., Study of Hellinger Distance as a splitting metric for Random Forests in balanced and imbalanced classification datasets, 2020, Expert Syst. Appl., 149, 113264, <https://doi.org/10.1016/j.eswa.2020.113264>

[14] Gretton, A., Borgwardt, K.M., Rasch, M.J., Schölkopf, B., Smola, A., A kernel two-sample test, 2012, J. Mach. Learn. Res., 13, 723-773

[15] Long, M., Cao, Y., Wang, J., Jordan, M., Learning transferable features with deep adaptation networks, 2015, in: International Conference on Machine Learning, PMLR, 97-105.

[16] Breiman, L., Friedman, J., Olshen, R. A., & Stone, C. J. 2017, Classification and regression trees, Routledge (London), ISBN: 9781315139470.

[17] Quinlan, J.R. 2014, C4. 5: programs for machine learning, Elsevier (Amsterdam), ISBN: 1-55860-238-0.

[18] Shimodaira, H., Improving predictive inference under covariate shift by weighting the log-likelihood function, 2000, J. Stat. Plan. Infer., 90, 227-244, <https://doi.org/10.1016/S0378-3758(00)00115-4>.

[19] Maaten, L.V.D., Hinton, G., Visualizing data using t-SNE, 2008, J. Mach. Learn. Res., 9, 2579-2605.

[20] Ziletti, A., Kumar, D., Scheffler, M., Ghiringhelli, L.M., Insightful classification of crystal structures using deep learning, 2018, Nat. Commun, 9, 2775, <https://doi.org/10.1038/s41467-018-05169-6>.

[21] Tshitoyan, V., Dagdelen, J., Weston, L., Dunn, A., Rong, Z., Kononova, O., Persson, K.A., Ceder, G., Jain, A., Unsupervised word embeddings capture latent knowledge from materials science literature, 2019, Nature, 571, 95–98, <https://doi.org/10.1038/s41586-019-1335-8>.

[22] Reed, R.C. 2006, The Superalloys: Fundamentals and Applications, 1st ed., Cambridge University Press (Cambridge), ISBN: 9780521859042.

[23] Zhu, L., Yu, X., Li, W., Zhang, L., Zhang, N., Lv, Y., Zhao, L., et al., High-throughput investigation of Nb and Ta alloying effects on the microstructure and properties of a novel Ni-Co-based superalloy, 2023, Scr. Mater., 226, 115215, <https://doi.org/10.1016/j.scriptamat.2022.115215>.

[24] Jiang, L., Wang, C., Fu, H., Shen, J., Zhang, Z., Xie, J., Discovery of aluminum alloys with ultra-strength and high-toughness via a property-oriented design strategy, 2022, J. Mater. Sci. Technol., 98, 33-43, <https://doi.org/10.1016/j.jmst.2021.05.011>.

[25] Lookman, T., Balachandran, P.V., Xue, D., et al., Active learning in materials science with emphasis on adaptive sampling using uncertainties for targeted design, 2019, npj Comput. Mater., 5, 21, <https://doi.org/10.1038/s41524-019-0153-8>.

[26] Seko, A., Togo, A., Hayashi, H., Tsuda, K., Chaput, L., Tanaka, I., Prediction of low-thermal-conductivity compounds with first-principles anharmonic lattice-dynamics calculations and Bayesian optimization, 2015, Phys. Rev. Lett., 115, 205901, <https://doi.org/10.1103/PhysRevLett.115.205901>.

[27] Yu, X., Sun, S., Tian, Y., Sample selection for noisy partial label learning with interactive contrastive learning, 2025, Pattern Recognit., 166, 111681, https://doi.org/10.1016/j.patcog.2025.111681.

[28] Gupta, V., Choudhary, K., Tavazza, F., et al., Cross-property deep transfer learning framework for enhanced predictive analytics on small materials data, 2021, Nat. Commun., 12, 6595, <https://doi.org/10.1038/s41467-021-26921-5>.

[29] Ramprasad, R., Batra, R., Pilania, G., et al., Machine learning in materials informatics: recent applications and prospects, 2017, npj Comput. Mater., 3, 54, <https://doi.org/10.1038/s41524-017-0056-5>.

[30] Jiang, L., Zhang, Z., Hu, H., He, X., Fu, H., Xie, J., A rapid and effective method for alloy materials design via sample data transfer machine learning, 2023, npj Comput. Mater., 9, 26, <https://doi.org/10.1038/s41524-023-00979-9>.

[31] Lakshminarayanan, B., Pritzel, A., Blundell, C., Simple and scalable predictive uncertainty estimation using deep ensembles, 2017, Adv. Neural Inf. Process. Syst., 30, <https://doi.org/10.48550/arXiv.1612.01474>.

[32] Salmerón, R., García, C., García, J., Overcoming the inconsistences of the variance inflation factor: A redefined VIF and a test to detect statistical troubling multicollinearity, 2020, arXiv preprint arXiv:2005.02245.

[33] Imbens, G.W., Statistical Significance, p-Values, and the Reporting of Uncertainty, 2021, J. Econ. Perspect., 35, 157–74, <https://doi.org/10.1257/jep.35.3.157>.

[34] Tan, L., Yang, X.G., Shi, D.Q., Hao, W.Q., Fan, Y.S., Unified fatigue life modelling and uncertainty estimation of Ni-based superalloy family with a supervised machine learning approach, 2022, Eng. Fract. Mech., 275, 108813, <https://doi.org/10.1016/j.engfracmech.2022.108813>.

[35] Greenwell, B.M., Boehmke, B.C., McCarthy, A.J., A simple and effective model-based variable importance measure, 2018, arXiv:1805.04755, <https://arxiv.org/abs/1805.04755>.

[36] Weiss, K., Khoshgoftaar, T.M., Wang, D., A survey of transfer learning, 2016, J. Big Data, 3, 9, <https://doi.org/10.1186/s40537-016-0043-6>.

[37] Liu, B., Zheng, Z., Xiao, Y., Sun, P., Li, X., Zhao, S., Huang, Y., Peng, T., Self-paced method for transfer partial label learning, 2024, Inf. Sci., 679, 121043, <https://doi.org/10.1016/j.ins.2024.121043>.

[38] Kim, I.S., Choi, B.G., Jung, J.E., Do, J., Jo, C.Y., Effect of microstructural characteristics on the low cycle fatigue behaviors of cast Ni-base superalloys, 2015, Mater. Charact., 106, 375-381, https://doi.org/10.1016/j.matchar.2015.06.011.

[39] Sui, S., Tan, H., Chen, J., et al., The influence of Laves phases on the room temperature tensile properties of Inconel 718 fabricated by powder feeding laser additive manufacturing, 2019, Acta Mater., 164, 413-427, <https://doi.org/10.1016/j.actamat.2018.10.032>.

[40] Cao, L., Chen, Y., Sun, Y., et al., Regional high temperature fatigue crack growth behavior of a microstructure-gradient nickel-based superalloy, 2024, Mater. Sci. Eng. A, 890, 145871, <https://doi.org/10.1016/j.msea.2023.145871>.

[41] Wang, X.G., Liu, J.L., Liu, J.D., et al., Dependence of stacking faults in gamma matrix on low-cycle fatigue behavior of a Ni-based single-crystal superalloy at elevated temperature, 2018, Scripta Mater., 152, 94-97, <https://doi.org/10.1016/j.scriptamat.2018.04.020>.

1. 1These authors contribute equally to this work.

   ^*^Corresponding author:

   E-mail: wudayong_ysu@126.com (D.Y. Wu), ziyuanrao@sjtu.edu.cn (Z.Y. Rao), sxru2008@hebust.edu.cn (R. Su). [↑](#footnote-ref-1)
